# Teasing apart trauma: neural oscillations differentiate individual cases of mild traumatic brain injury from post-traumatic stress disorder even when symptoms overlap

**DOI:** 10.1038/s41398-021-01467-8

**Published:** 2021-06-04

**Authors:** Jing Zhang, Zahra Emami, Kristina Safar, Patrick McCunn, J. Don Richardson, Shawn G. Rhind, Leodante da Costa, Rakesh Jetly, Benjamin T. Dunkley

**Affiliations:** 1 Department of Diagnostic Imaging, Hospital for Sick Children, Toronto, ON Canada; 2 Neurosciences & Mental Health, SickKids Research Institute, Toronto, ON Canada; 3 St Joseph’s, London OSI, London, ON Canada; 4 MacDonald Franklin OSI Research Centre, London, ON Canada; 5 Defence Research and Development Canada, Toronto, Canada; 6 Sunnybrook Health Sciences Centre, Toronto, ON Canada; 7 Department of Surgery, University of Toronto, Toronto, ON Canada; 8 Canadian Forces Health Services HQ, Ottawa, Canada; 9 Department of Psychiatry, Faculty of Medicine, University of Ottawa, Ottawa, ON Canada; 10 Department of Psychiatry, Faculty of Medicine, Dalhousie University, Halifax, NS Canada; 11 Department of Medical Imaging, University of Toronto, Toronto, ON Canada

**Keywords:** Neuroscience, Biomarkers

## Abstract

Post-traumatic stress disorder (PTSD) and mild traumatic brain injury (mTBI) are highly prevalent and closely related disorders. Affected individuals often exhibit substantially overlapping symptomatology – a major challenge for differential diagnosis in both military and civilian contexts. According to our symptom assessment, the PTSD group exhibited comparable levels of concussion symptoms and severity to the mTBI group. An objective and reliable system to uncover the key neural signatures differentiating these disorders would be an important step towards translational and applied clinical use. Here we explore use of MEG (magnetoencephalography)-multivariate statistical learning analysis in identifying the neural features for differential PTSD/mTBI characterisation. Resting state MEG-derived regional neural activity and coherence (or *functional connectivity*) across seven canonical neural oscillation frequencies (delta to high gamma) were used. The selected features were consistent and largely confirmatory with previously established neurophysiological markers for the two disorders. For regional power from theta, alpha and high gamma bands, the amygdala, hippocampus and temporal areas were identified. In line with regional activity, additional connections within the occipital, parietal and temporal regions were selected across a number of frequency bands. This study is the first to employ MEG-derived neural features to reliably and differentially stratify the two disorders in a multi-group context. The features from alpha and beta bands exhibited the best classification performance, even in cases where distinction by concussion symptom profiles alone were extremely difficult. We demonstrate the potential of using ‘invisible’ neural indices of brain functioning to understand and differentiate these debilitating conditions.

## Introduction

Posttraumatic stress disorder (PTSD) and traumatic brain injury (TBI) are prevalent and commonly comorbid conditions in which clinical symptoms often overlap, creating major challenges in their diagnosis and treatment^1^. These complex disorders frequently co-occur in both civilian and military populations and share similar aetiology and symptomatology^1,2^ – both having their origins in trauma, one from psychological stress and the other from physical injury^3–5^. Indeed, the nexus between PTSD and mild TBI (mTBI) has become a major focus of clinical research interest in recent years^6–8^. Notably, PTSD and mTBI occur at considerably high rates among combat-exposed military members and Veterans returning from wars in the Middle East and together have been referred to as “signature injuries” of modern military conflicts^9–11^. Both disorders can cause serious functional impairments and impart significant disruption to daily life, regardless of economic development status^3–5^.

Given the lack of objective markers for PTSD and mTBI, as well as the overlapping symptoms, the disentanglement of these disorders can be challenging^12^. Even when these injuries occur independently and in the absence of the other, comparing across individuals can reveal self-reported symptom profiles that substantially overlap^1,13^, making a differential clinical diagnosis difficult. As with PTSD, persistent post-concussive symptoms (PPCS) of an mTBI are often non-specific and can mimic other psychiatric disorders^7^. Patients with PTSD suffer intrusive memories, hypervigilance and moral injury^14^. Heterogeneous mTBI symptoms include headaches, light sensitivity, tinnitus amongst others^15^. However, negative alterations in cognition and mood, behavioural impairment, sleep disturbances, avoidance and emotional lability are common in both conditions, as well as comorbid secondary anxiety and depression that parallel the main diagnosis^15^. Moreover, mTBI may also lead to emotional numbing, derealization, depersonalisation and amnesia, which are some of the dissociative symptoms more commonly identified with PTSD^16–18^. Symptoms of PTSD can mirror a number of other psychiatric disorders, including anxiety and depression^19^, and perhaps surprisingly, even without a history of head injury, PTSD patients often report symptoms mimicking an mTBI^14^. Other comorbid disorders alongside PTSD and mTBI also add to the already challenging differential diagnosis^20,21^. An accurate, fast, differential diagnosis carries important implications, as treatment regimens are markedly diverse, and it takes time to establish an effective routine; trajectories of remission and recovery diverge and mismanagement can prolong oftentimes debilitating functional impairment^19^. Therefore, the current study focuses on differential PTSD and mTBI classification versus several control groups. Further, this work also presents a functional brain imaging-informatics framework as a “first step” towards solving the various multiclass problems, and thus could be adopted in the future for scenarios including co-occurrence.

Even though brain structural abnormalities for the two disorders^22,23^ have been reported using magnetic resonance imaging (MRI), these studies report group-level effects, and such approaches do not provide enough information for an individual diagnosis in a clinical setting^24^. In addition, the lack of sensitive, validated assessment tools for brain plasticity and recovery complicates clinical trials studying potential treatments for persistent PTSD and mTBI symptoms^25^. Despite the lack of apparent anatomical indicators, ongoing symptomatology suggests persistent underlying neurophysiological dysfunction. Functional imaging technique such as fMRI, EEG, and MEG have shown promising results in representing the two disorder in binary case vs control settings^26–30^. Functional indices of neural activity are potentially powerful candidates for both understanding the pathophysiology of somatic, cognitive and behavioural complaints, and in providing reliable markers for developing objective diagnostic systems^31^, as well as effective treatment targets, such as rTMS^32^ and neurofeedback^33^. Neural activity and dynamics can be uniquely attributed and related to symptoms in both disorders^34^, and group differences are found when these conditions are compared^35^. However, individual identification and reliable stratification with neurophysiological features has not yet been achieved - objective, measurable and easy-to-use biomarker system would be a major advancement in supporting an accurate differential diagnosis, particularly one that can be acquired non-invasively, quickly, easily, is well-tolerated and does not cause undue stress to the patient^36^.

Multivariate learning approaches have shown to be effective in neuropsychiatric and neurodegenerative disease classification in a multiclass (i.e. multiple groups) context. Support vector machine (SVM) classifiers using EEG features can distinguish PTSD and major depressive disorder, with promising performance^37^. Moreover, using similar informatics approaches, fMRI has been shown to be effective in differentiating comorbid PTSD and mTBI^38^. A novel and yet unused approach would be neuroimaging- and multivariate statistics learning-driven feature selection and modelling of PTSD and mTBI. The current study advances research in this field aligning with the future research priorities and directions of military healthcare and the important treatment implications of accurately diagnosing these disorders^19,39^.

We applied our recently developed feature selection and modelling pipeline^29^, with modifications, to MEG neurophysiological resting state data that captures multiple macroscopic elements of neural functioning. MEG was used to measure two specific types of neural activity – the first, based on source-localised neural oscillatory power, provides a measure of regional, or *segregated* neural function generated by meso-scale circuits operating in discrete brain areas. The second type involves a type of functional connectivity measure, as an index of macro-scale *integrated* networks and circuits in the brain, based on amplitude envelope correlations, a method of ‘*communication-through-coherence*’^40^. These two rich and multifaceted data types allow us to test which form of neural activity offers the superior modelling configuration and classification performance, and which features maximally differentiate groups, while elucidating some of the distinct neurophysiological correlates of these disorders. The data were assessed across multiple frequency ranges, from the delta through high gamma bands, each known to play important roles in the dynamic repertoire of brain function. Taken together, this study offers an exciting proof-of-principle approach and tests the optimal paradigm conditions for disassociating PTSD and mTBI, using neural markers in the presence of overlapping symptom profiles.

## Materials and methods

### Participants

Four groups were included: PTSD, TC (trauma-exposed controls), mTBI and NTC (non-trauma controls). The PTSD and TC participants were active military personnel at the time of recruitment, all from the Canadian Armed Forces (CAF). The mTBI and NTC participants were from civilian settings.

For the PTSD and TC groups, we recruited 24 male soldiers with PTSD (mean age ± SD = 33.05 ± 5.26), and 27 male peers exposed to similar operational stress, deployment roles and traumatic conditions but who did not developed PTSD (mean age ± SD = 37.4 ± 6.8). Further details for the TC and PTSD groups can be found in Dunkley et al.^26^. All PTSD and TC participants were recruited through the Canadian Armed Forces and Operational and Trauma Stress Support Centres (OTSSC). The TC participants were matched with the PTSD group on military rank & experience, education level and handedness.

For the mTBI and NTC groups, 27 male civilian adults with mTBI (mean age ± SD = 29.6 ± 6.7) and 23 typical male civilian adults (mean age ± SD = 28.0 ± 5.6) were recruited. The NTC group were matched with the mTBI group on age, sex and handedness. All mTBI patients were recruited through Sunnybrook Health Science Centre (Toronto, Ontario, Canada), Canada’s largest head injury trauma centre. Further details for the NTC and mTBI groups can be found in Zhang et al.^30^. All experimental procedures were approved by the Research Ethics Board at the Hospital for Sick Children, Sunnybrook Hospital and OTSSC in accordance with the Helsinki Declaration on Research Ethics. All participants provided written informed consent.

### Diagnoses and inclusion criteria

This information can be viewed in Supplementary Methods.

### Magnetoencephalography

A detailed description of the MEG methods can be found in the Supplementary Methods, including MEG acquisition and signal processing. ‘Virtual sensor’ time series were modelled at node locations and regional power spectrum density (PSD, “regional power”) was calculated using Welch’s method on each 10 s epoch of time series data. The final regional power spectrum was obtained by averaging over the 10 s epochs. For band-limited analyses, time series were filtered into seven frequency ranges: delta (1–3 Hz), theta (4–7 Hz), alpha (8–14 Hz), beta (15–30 Hz), low gamma one (30–55 Hz), low gamma two (65–80 Hz) and high gamma (80–150 Hz)*.* Functional connectivity was calculated using the amplitude envelope correlation (AEC), a robust, repeatable and reliable measure when compared to other definitions of functional connectivity^41^; further, it recapitulates spontaneous resting brain networks derived from more commonly user fMRI BOLD resting state network analysis^42^, facilitating comparison with studies using those measures.

### Multivariate statistics learning and feature selection analysis

**Fig. 1:**
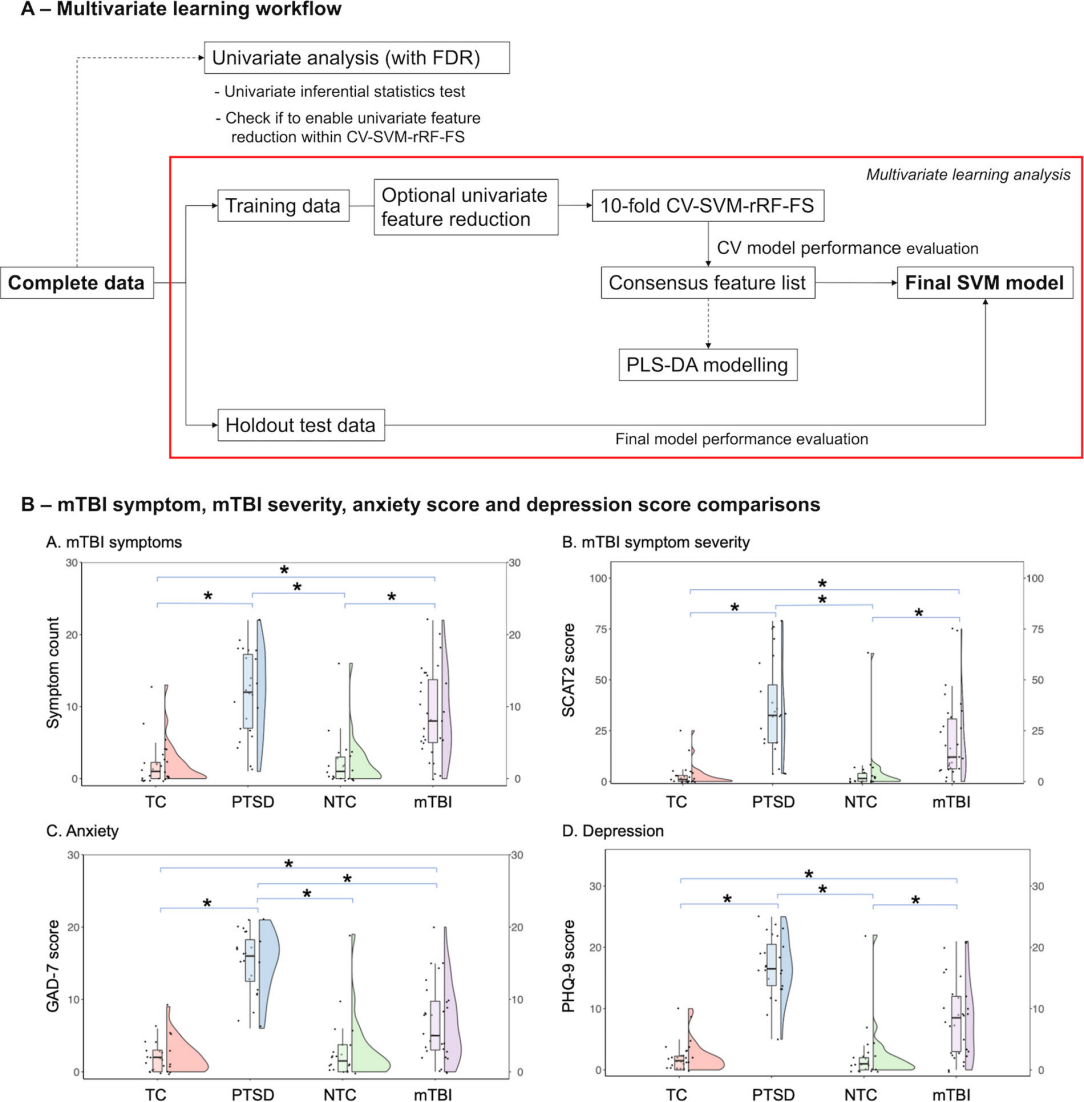
Overall downstream informatics workflow and symptom analysis. **A** A flowchart showing overall downstream informatics analysis workflow. **B** Concussion, anxiety and depression symptom profiles overlap in PTSD and mTBI. Rain cloud plots showing mTBI symptom number, mTBI severity, anxiety and depression score for the mTBI, PTSD, TC and NTC groups. Raw data (dots), boxplot and probability distribution are plotted. No statistical difference was observed for PTSD and mTBI in mTBI symptoms. PTSD participants (without head injury) reported significantly higher mTBI symptom severity than the mTBI participants. Although PTSD participants exhibited significantly higher anxiety and depression scores than the mTBI participants, both PTSD and mTBI groups scored significantly higher than both controls groups in the two scales. *=statistically significant (*p*â€‰<â€‰0.05) differences based on the ANOVA with Tukey post-hoc test. FDR false discovery rate, CV-SVM-rRF-FS support vector machine and recursive random forest feature selection with cross validation, PLS-DA partial least squares discriminate analysis, NTC non-trauma control, TC trauma-exposed control, SCAT2 Sports Concussion Assessment Tool 2, GAD-7 Generalised Anxiety Disorder 7, PHQ-9 Patient Health Questionnaire.

Based on Zhang et al.^29^, an overview of the informatics workflow is displayed in Fig. 1A. The analysis was conducted separately on the two MEG measurements.

#### Data resampling and univariate analysis

All participants and all features are considered the “complete data”. For classification modelling, subset data featuring 85% of participants was used as the “training data”, with the rest (15%) used as the “holdout test data”. The training data was used to derive multiclass classification models, whose final performance was determined by the holdout test data. The training and holdout test data split was achieved by stratified random data resampling. Details on the univariate analysis can be found in Supplementary Methods.

#### Univariate analysis

The detailed description of the univariate analysis method can be found in the Supplementary Methods. Here we describe the implementation. As shown in Fig. 1A, the present study utilised univariate analysis as (i) a standalone analysis and (ii) as part of the ML process. When used independently, the univariate analysis was applied to the complete data, and in two ways: (a) the analysis was carried out on the complete data, evaluating whether group differences could be represented by univariate reduced features; and (b) the univariate analysis was conducted to help determine the optimal feature selection setting for ML. i.e. one step (CV-SVM-rRF-FS, or support vector machine and recursive random forest feature selection with cross validation) vs two-step (univariate feature reduction + CV-SVM-rRF-FS). The two-step process was designed to reduce the computational burden during ML. In such a case, the univariate statistics process was carried out again but only on the training data. As such, the holdout test set was not part of this univariate feature reduction, or any subsequent feature selection/training steps, thereby appropriately avoiding information leakage. This means the final selected features were only reached by the training data.

#### Feature selection with support vector machine and cross validation

The analysis was conducted through SVM modelling, validation and performance assessment steps^29^. Nested 10-fold cross validation (CV) was used. For feature selection, the core algorithm was a recursive random forest feature selection (rRF-FS) procedure^43^. As described in Zhang et al.^29^, the rRF-FS-driven features selection was included in each CV iterations (i.e. “CV-SVM-rRF-FS”). Upon feature selection, PLS-DA (partial least squares discriminant analysis) was carried out as an independent classifier to verify the modelling generalisability of the selected features. Ultimately, a final SVM model was trained on all the training data with the consensus features and optimised kernel type.

For model evaluation, firstly, a sample label permutation test was used^44^. A permutation *p*-value was also calculated^44^. Models with a permutation *p*-value < 0.05 were considered significant. Next, the final SVM models were assessed using the holdout test data. The holdout data was excluded from any training steps. Per-participant percentage accuracy was used for both CV and final SVM model evaluation. Moreover, ROC-AUC (receiver operating characteristic curve-area under the curve) was determined for both models to assess sensitivity and specificity across classification thresholds, i.e. versatility. These tests assessed the models’ capability to accurately classify data into either the participant group of interest or the remaining three groups. Additional details can be viewed in Supplementary Methods.

### Additional statistical analysis

Statistical analysis for mTBI symptoms & severity (Sports Concussion Assessment Tool 2; SCAT2)^45^, anxiety (Generalised Anxiety Disorder 7; GAD-7)^46^ and depression (Patient Health Questionnaire 9; PHQ-9)^47^, as well as model performance comparisons can be found in Supplementary Methods.

## Results

The key results are described here, and additional results can be found in Supplementary Results.

### PTSD and mTBI overlap in their symptom profiles and screeners fail to distinguish individuals

There was no significant difference between the PTSD and mTBI groups for mTBI symptom number, with substantial overlap in their distributions (Fig. 1B). Interestingly, compared to the mTBI group, the PTSD group reported a slightly *higher* (albeit non-significant) mean number of symptoms consistent with mTBI, despite having not experiencing a head injury. Even some of the typical civilian control participants reported symptoms of head injury – this emphasizes the limitation of relying solely on symptom questionnaires and the clinical difficulty in making a differential and categorical diagnosis^48^. For mTBI severity, the PTSD group showed *higher*, but comparable (non-significant) scores compared to the mTBI group (Fig. 1B). Furthermore, mean anxiety and depression scores were higher in PTSD compared to mTBI (Fig. 1B), but their distributions still overlapped, meaning that for any given individual, it would be virtually impossible to determine if they belong to one group or another based on these data alone. As expected, both the PTSD and mTBI groups exhibited (mostly) significantly higher mean anxiety and depression scores when compared to the control groups (Fig. 1B).

### Univariate statistics fails to tease apart group differences in either power or connectivity

**Fig. 2:**
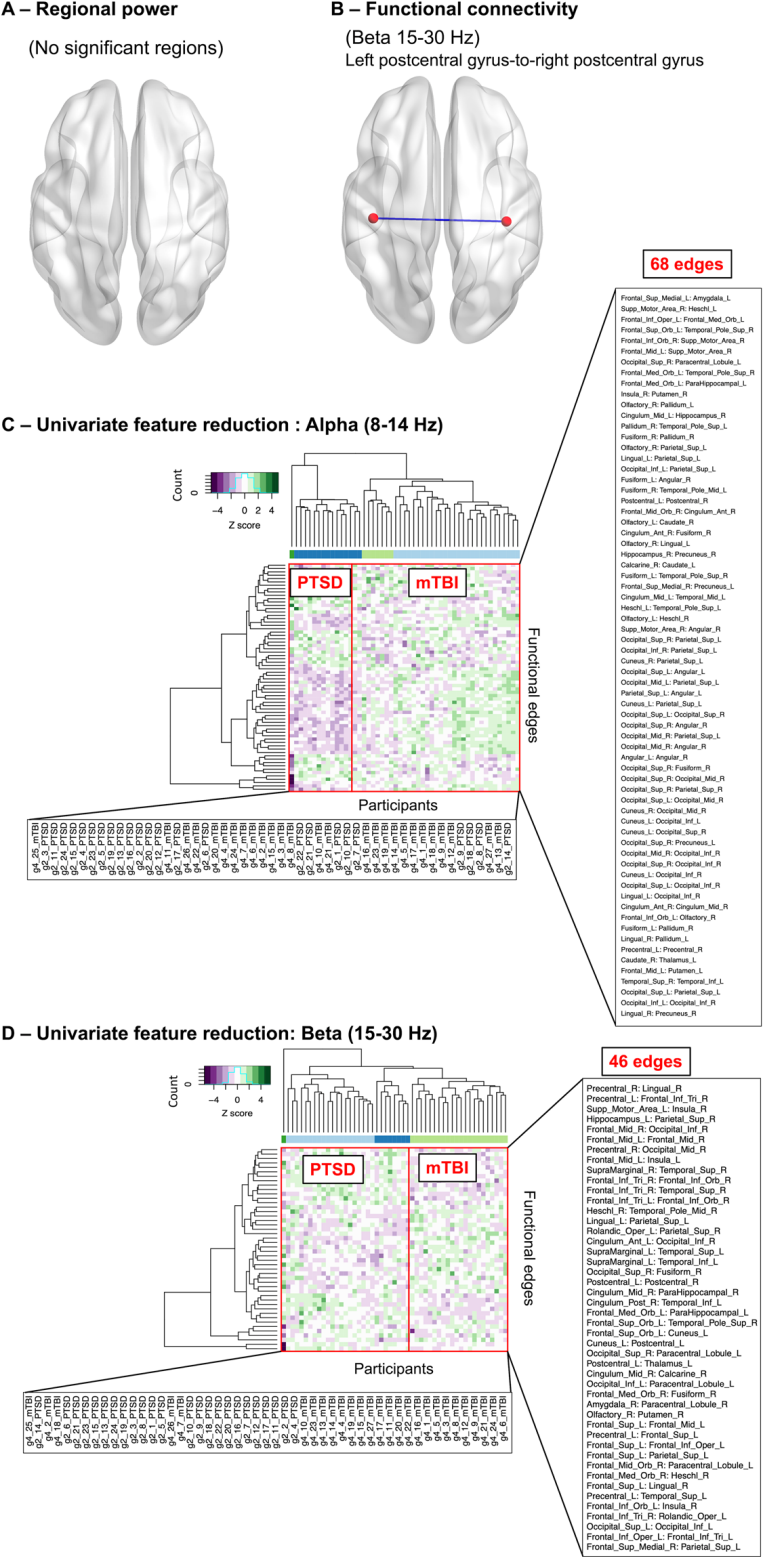
Univariate statistical analysis and univariate feature reduction hierarchical clustering analysis. **A**, **B** Univariate analyses only reveal group differences in connectivity, and no regional change. **A** Regional power did not show any significant effects (correct for multiple comparisons), whereas **B** functional connectivity, for the beta band, shows only a single significant connection connecting the left postcentral gyrus-to-right postcentral gyrus. **C**, **D** Unsupervised hierarchical clustering results show univariate reduced data led to better group separation with the functional connectivity data. The figure shows the (**C**) alpha and (**D**) beta bands as examples, with the PTSD vs mTBI contrast. The dendrograms show the clustering for the participants (horizontal) and the univariate reduced features (vertical) based on the Z scores for AEC. The colour bar below the horizontal indicates the major (top three levels) participant clusters. The number of univariate reduced features are marked. Both **C** and **D** showed that, although with some exceptions, the univariate feature reduction helped reduce the functional connectivity data into features that separated the participants into PTSD and mTBI groups.

Clustering analyses on the complete MEG data space does not cluster according to the four participant groups, for either of the neural measures (full feature count: regional power: 90 brain areas, functional connectivity: 4005 edges). The statistical significance was determined by thresholding FDR-corrected *p*-values (alpha = 0.05). Only the functional connectivity data was found to contain statistically significant results, i.e. a single connection between the left postcentral gyrus-to-right postcentral gyrus (Fig. 2A, B). The complete results can be viewed in Supplementary Tables S1 and S2. Supplementary Figs. S1 and S2 contain overall and univariate statistic-reduced power and connectivity profile distribution heatmaps, respectively.

### Feature selection identifies the most relevant neural markers for modelling

Univariate analysis (with raw *p*-value thresholding) was conducted on the complete data to test if the initial univariate feature reduction was needed during feature selection. Only the functional connectivity showed group separation upon univariate reduction (Fig. 2C, D, Supplementary Figs. S3 and S4), suggesting a tangible benefit from the initial univariate reduction step during feature selection for this data type.

**Fig. 3:**
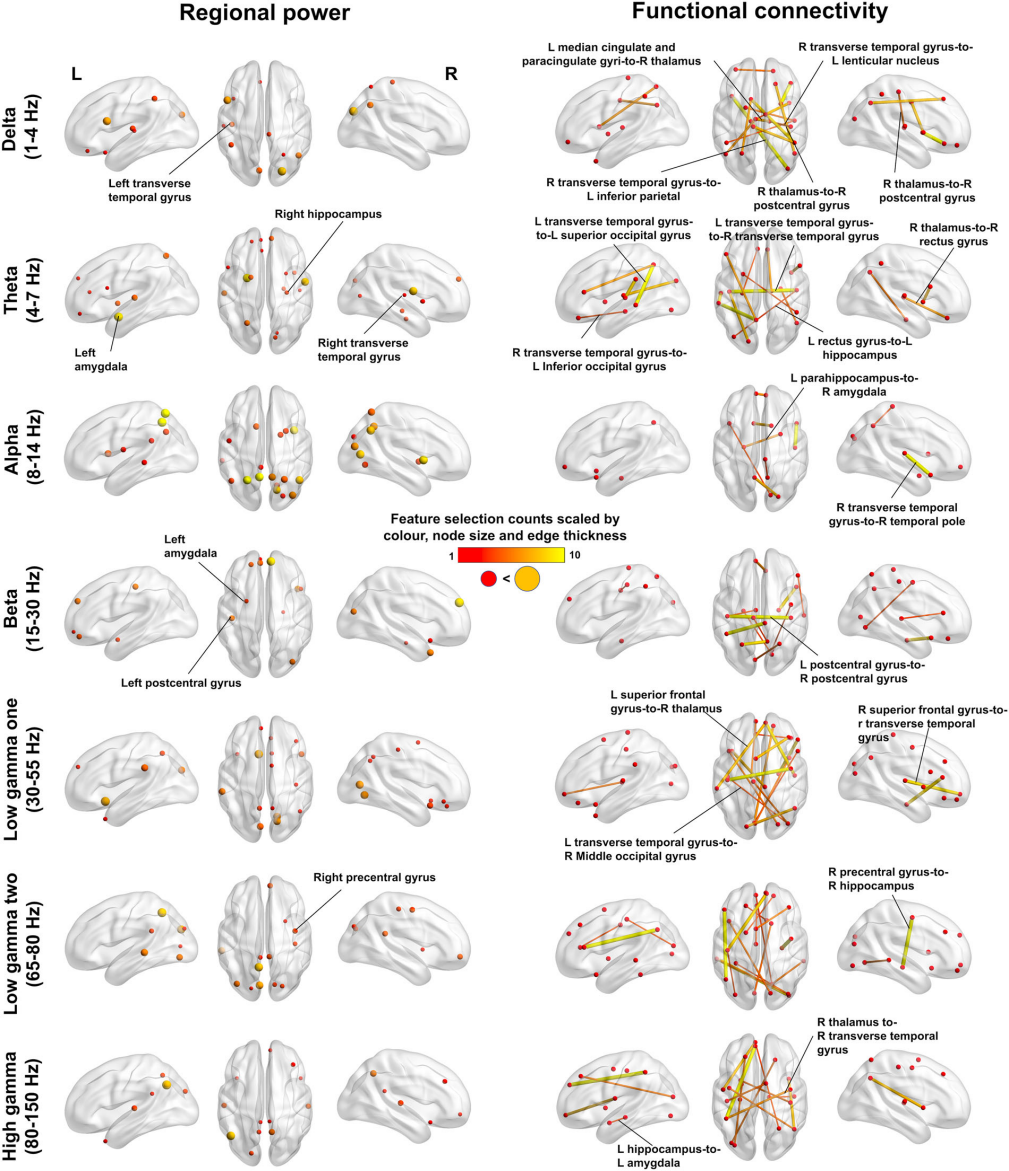
Functional connectivity outperforms in feature selection count compared to regional functioning. Regional power and functional connectivity maps with only the CV-SVM-rRF-FS selected features for all seven frequency bands, with the most biologically relevant features marked. Thalamus, amygdala, hippocampus and superior temporal gyrus (i.e. transverse temporal gyrus) are marked for regional power figures; functional edges containing these regions are also marked for the functional connectivity figures. As identified by both data types, the right precentral gyrus was marked for the regional power and functional connectivity figures at the high gamma band. For the beta band, the statistically significant edge left postcentral gyrus-to-right postcentral gyrus (marked) was also identified by feature selection. CV-SVM-rRF-FS support vector machine and recursive random forest feature selection with cross validation.

Figure 3 and Supplementary Table S3 display the final consensus features selected by CV-SVM-rRF-FS for both neural feature sets, at each canonical frequency. For example, regional power in the theta, alpha and high gamma bands residing in the amygdala, hippocampus and temporal areas were identified as key features (Supplementary Table S3A and Fig. 3). Additionally, delta activity in the left transverse temporal gyrus was also identified as a distinguishing feature, as well as beta activity in the left amygdala. Figure 3 and Supplementary Table S3B include the selected feature lists found for functional connectivity. Largely consistent with the regional data, connections involving the thalamus, hippocampus, amygdala and temporal areas were selected (Fig. 3). The detailed feature selection and PLS-DA results are included in the Supplementary Results and Supplementary Figs. S5 and S6.

### Multiclass classification modelling of neural functioning shows promising performance

**Fig. 4:**
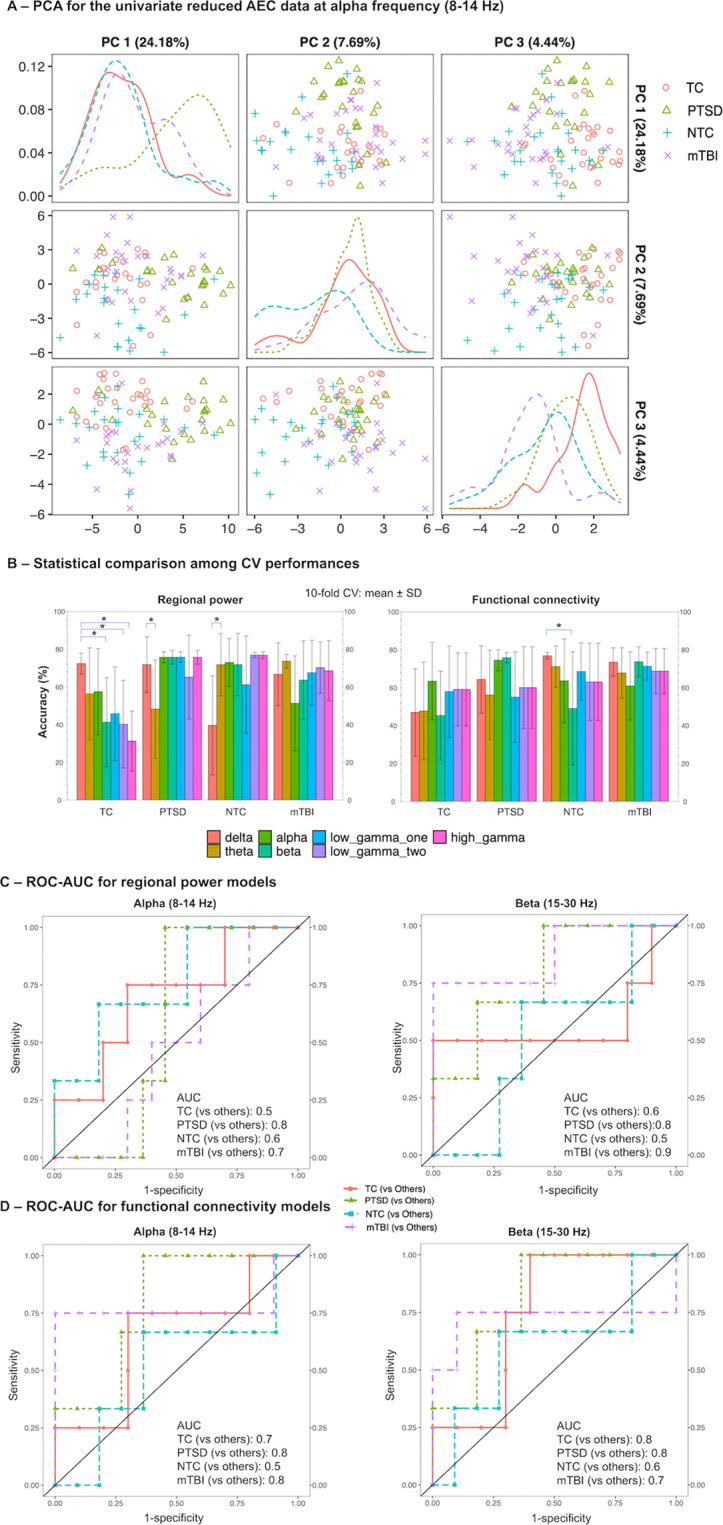
Machine learning feature selection clustering and classification modelling results. **A** PCA on univariate reduced data starts to separate the groups. PCA on the complete functional connectivity data with only univariate reduced features for the alpha band. Three principal components (PC1-3) were plotted. The diagonal shows density distribution of the PCA scores for the four participant groups. The plot shows four participant groups start to separate with univariate reduced data. **B** SVM CV model classification accuracy reached comparable values for both feature types. Bar graphs showing per group classification accuracies for the CV models comparing all seven frequency bands, regional power and functional connectivity. *For each participant group*, *=statistically significant (*p*â€‰<â€‰0.05) differences based on the ANOVA with Tukey post-hoc test. **C** Alpha and beta power show robust model versatility. ROC-AUC analysis results for the final regional power model at the alpha and beta frequency bands with the holdout test data. The plot shows that models reached good versatility for both disorders (AUC: 0.7–0.9). **D** Alpha and beta connectivities in the brain are also versatile in differentiating overlapping disorders. ROC-AUC analysis results for the final functional connectivity models for alpha and beta frequency with the holdout test data. The plot shows that models reached good versatility for both disorders (AUC: 0.7~0.8). PCA principal component analysis, PC principal component, ROC receiver operating characteristic, AUC area under the curve, SD standard deviation, NTC non-trauma control, TC trauma-exposed control.

PCA results were shown in Fig. 4A and Supplementary Fig. S4. CV models were compared across frequencies using ANOVA with Tukey post-hoc test (Fig. 4B). For CV models derived from regional power data, the theta band was outperformed by other frequency bands when it comes to PTSD classification accuracy, whereas all frequency bands performed comparably for mTBI (Fig. 4B). For functional connectivity, no statistically significant differences were found in the classification accuracy for both disorders (Fig. 4B). Table 1A and Supplementary Table S4 contain the per group classification accuracy (%, mean ± SD) and AUC results, respectively. Additional ML results can be viewed in Supplementary Figs. S7–S9.

**Table 1:** A. SVM modelling CV per participant group accuracy (%, mean ± SD); B. SVM modelling final model per participant group accuracy (%) on holdout test data.

A. SVM modelling CV per participant group accuracy (%, mean ± SD)				
Frequency	TC	PTSD	NTC	mTBI
*Regional power*				
Delta	72.42 ± 5.54	71.89 ± 14.78	39.67 ± 26.23	66.75 ± 16.67
Theta	56.39 ± 24.35	48.33 ± 26.02	71.83 ± 16.58	73.78 ± 3.63
Alpha	57.53 ± 22.82	75.78 ± 2.92	73.03 ± 12.69	51.33 ± 25.19
Beta	41.33 ± 23.62	75.78 ± 3.64	71.89 ± 16.56	63.67 ± 20.82
Low gamma one	45.83 ± 24.88	75.83 ± 2.71	61.28 ± 25.75	67.61 ± 17.16
Low gamma two	40.03 ± 23.21	65.28 ± 22.23	76.94 ± 1.34	70.28 ± 13.67
High gamma	31.33 ± 15.94	75.78 ± 3.64	76.89 ± 1.76	68.67 ± 15.94
*AEC*				
Delta	47.03 ± 23.02	64.39 ± 17.75	76.89 ± 1.75	73.53 ± 7.50
Theta	47.75 ± 25.72	56.19 ± 23.55	71.19 ± 10.87	67.81 ± 13.27
Alpha	63.53 ± 20.37	74.53 ± 5.57	63.67 ± 22.1	60.94 ± 17.97
Beta	45.39 ± 23.5	75.83 ± 2.71	48.11 ± 29.86	73.72 ± 7.93
Low gamma one	58.00 ± 24.03	55.06 ± 23.77	68.56 ± 15.17	71.33 ± 7.34
Low gamma two	51.96 ± 18.11	70.53 ± 15.05	69.53 ± 12.92	57.42 ± 26.29
High gamma	59.17 ± 19.28	60.14 ± 21.53	63.11 ± 20.38	68.81 ± 11.89

B. SVM modelling final model per participant group accuracy (%) on holdout test data				
*Regional power*				
Delta	50.00	71.43	78.57	85.14
Theta	64.29	71.42	78.57	57.14
Alpha	50.00	85.71	50.00	85.71
Beta	64.29	71.43	71.43	92.86
Low gamma one	64.29	78.57	42.85	71.43
Low gamma two	57.14	50.00	57.14	64.86
High gamma	42.86	71.43	57.14	57.14
*AEC*				
Delta	71.43	57.14	64.29	50.00
Theta	78.57	85.71	71.43	78.57
Alpha	57.14	71.43	57.14	85.71
Beta	78.57	78.57	42.86	85.71
Low gamma one	28.57	78.57	64.29	57.14
Low gamma two	57.14	71.43	71.43	57.14

Using the consensus features, the final multiclass classification SVM models were generated for all frequency bands and data types. These models were ultimately evaluated with the holdout test data (Table 1B).

For the regional power models, Table 1B demonstrates that all but the low gamma two (65–80 Hz) band exhibited over 70% accuracy for PTSD classification, with the alpha model showing the best performance (>85%). For mTBI classification, the top performing frequency bands were delta, alpha and beta bands (~85–90% accuracy), while the low gamma one band exceeded 70% accuracy (Table 1B). More importantly, the alpha and beta bands performed well for both disorders. In terms of ROC-AUC analysis (Fig. 4C and Supplementary Fig. S10), the alpha and beta bands also achieved good AUC values (~0.7–0.9).

All functional connectivity models exhibited over 70% accuracy for classifying the PTSD group, whereas the same level of performance was reached by the theta, alpha and beta oscillatory models for mTBI (Table 1B). When using the functional connectivity data, the alpha and beta models were the best models for differential PTSD and mTBI classification, even in the presence of overlapping symptom profiles that mimic each other (Fig. 4D and Supplementary Fig. S11).

## Discussion

### Summary

Differentiating a traumatic psychological stress injury from a physical ‘mild’ traumatic brain carries important clinical implications – their treatment regimens differ, as do their long-term outcomes. However, dissociating them is often difficult as their symptom profiles often overlap - as shown here - where soldiers with traumatic stress injuries report symptoms of a concussion (Fig. 1B), *without having suffered a head injury*. These results attest to the potential unreliability of basing a diagnosis on self-reported symptom screeners. Additionally, both PTSD and mTBI groups exhibited overall higher anxiety and depression scores than the control groups, showing that both disorders carry significant neuropsychiatric comorbidity, again highlighting the difficulty in making differential diagnoses from each other, and commonly co-occurring disorders. An accurate diagnosis of either disorder, amongst others, requires a comprehensive decision-making process that includes multiple types of information, including clinical interview and medical history.

We applied a feature selection and modelling pipeline (based on Zhang et al.^29^) to MEG regional neural oscillatory power and functional connectivity/communication data for differential classification of PTSD-mTBI in the presence of symptom overlap. The major findings were: (a) univariate statistics (e.g. conventional tests) alone are insufficient to produce reliable features for accurate differential classification; (b) the feature selection workflow identifies the most relevant features for classification in a multiclass(e.g. multiple group) setting, which have known neurobiological significance in the two disorders, including areas such as the amygdalae, thalamus, and hippocampi, and/or functional connections involving these regions, at frequencies of neural dynamics that index neural dysregulation, and are known to be pathophysiological in these disorders; (c) through the holdout test set assessment (analogous to a “real world” setting), models derived from both feature types showed promising classification accuracy at the alpha and beta frequencies, even when symptom profiles do not differentiate (e.g. reporting symptoms of a concussion when none has occurred, in the case of those with PTSD).

### Univariate statistics of neural functioning are insufficient for teasing apart traumatic injuries

The unsupervised clustering and univariate statistical analyses assessed the data variance and distribution properties, which are critical for confirming data consistency and, in turn, reliable naïve subject classification once the optimal classification models were generated.

First, the unsupervised hierarchical clustering and PCA results showed no clear participant separation when using the complete data space for both feature types. Next, statistical analysis revealed feature type-specific results. With no statistically significant regions identified in any frequency bands, the univariate analysis results showed that the variance of the region activity was insufficient to differentiate the four participant groups. Although group differences in MEG regional power were identified for PTSD and mTBI in a binary setting (“case vs control” studies)^49–51^, where mid-to-slow wave frequencies and bilateral postcentral areas were implicated as differentiating factors (from healthy controls), our analysis showed that this feature type failed to produce reliable separation in a multi-patient group context. This is likely due to the added data variance in the presence of additional patient/control groups. Moreover, the elevated data variance might also stem from heterogeneity of these disorders. For example, a recent study subtyped PTSD using multi-domain data^52^ and another study identified a PTSD subtype with verbal memory impairment, with unique ventral attention network connectivity established via fMRI^53^. For mTBI, a recent study proposed a need for a better subtype representation for the symptom-rating scales^54^, which was backed by a proposal of at least six sport-related concussion subtypes^55^.

For the functional connectome data, however, perhaps unexpectedly, the bilateral postcentral gyrus connection emerged as statistically significant across the four participant groups. Nevertheless, previous reports have implicated these areas in both disorders in binary “case vs control” studies. For example, a task-based fMRI study discovered dysregulated postcentral gyrus inhibition functions for the PTSD group, potentially related to the impaired execution of the stop response upon task for the PTSD patients^56^. For the mTBI patients, the dysregulated postcentral gyrus was linked to the cognitive functions, as shown in an attentional task-based MEG study^51^. Leveraging the MEG functional connectivity data type, our result demonstrated the effectiveness of our univariate statistics in identifying well-established connectivity signatures for the disorders, especially in a multiclass setting. However, with only two frequency bands showing significant effects, it is possible that additional information critical for differentiating traumatic injuries were yet to be discovered in the data.

Overall, univariate statistics applied to regional and global neural activity indices appears insufficient for categorically differentiating individuals with PTSD (with concomitant head injury symptoms) from mTBI. With added data variance from both multiple patient groups and potential presence of subtypes for both disorders, it is not a surprise that conventional univariate statistics failed to identify MEG-derived neural signatures, and that a more powerful multivariate and multiclass machine learning approach was warranted for differential classification.

### Multivariate learning-based feature selection identifies the most important neural features for teasing apart trauma

Due to the insufficient group separation based on univariate statistics, we expanded on the multivariate approach^35^, with a multivariate learning-based feature selection framework for modelling that can begin to reliably differentiate these insidious “invisible injuries”. It is worth noting that our feature selection process identifies the most important features based solely on the input data without requiring a priori knowledge input of the specific brain circuits that might be affected in these disorders. As such, without needing to specify individual areas and connections, our workflow presents a powerful generalisable and intuitive solution for feature selection and the subsequent classification modelling.

For regional activity, our feature selection procedure identified the most relevant brain regions for classification modelling. The selected brain regions align with previous studies showing abnormalities in the disorders, including the hippocampus, amygdala, and temporal gyri^57^. For the functional connectome data, the final feature list included connections linking the hippocampus, thalamus, amygdala, and temporal areas, all of which are involved in the core symptom profiles of PTSD and mTBI^35,58–61^. For example, the hippocampus is particularly susceptible to physical injury (e.g. brain trauma^62^), chronic stress^63^, and some of the core symptoms of PTSD, including traumatic re-experiencing and intrusive episodic memory, and the subsequent “knock-on” cognitive sequalae such as memory deficits and cognitive dysfunction^64^. Hippocampal circuits exhibit dysregulated neural activity, in correlational and casual studies, linked to TBI related memory deficits^65–69^. The thalamus is a central “relay station” for coordinating information between sensory, motor and myriad brain regions, supporting various brain functions^70^, and reciprocal thalamocortical connections are vulnerable to injury and stress^59,69^. Thalamic dysfunction is linked to the symptoms of both PTSD and mTBI. The amygdala serves emotion processing, with hyperexcitability in this region directly linked emotional dysfunction, maladaptive threat response and hypervigilance in PTSD^64^. Additionally, temporal areas are involved in the presentation and pathogenesis of PTSD, due to its role in perceptual functioning and emotional-memory linkages^71,72^ – likewise, these areas are known to be to be associated with similar pathology in mTBI^73^ – yet, crucially, neurophysiological features in our data from these same areas, but at specific frequency bands, as identified by this study, can separate the two. Therefore, the important point here is that data-driven neurophysiological modelling identifies the dysrhythmic neural features in key, overlapping brain areas involved in the disorders, and ultimately, dysregulated neural oscillations are able to differentiate the two. Our study recapitulates these previous reports by demonstrating the important role these areas play in classifying these disease states and extends them by revealing the frequency-specific neural markers that can distinguish imitable but distinct pathophysiology.

While effective in the context of SVM modelling, the PLS-DA modelling and its permutation test suggested that, for regional activity, the selected features were only suited for SVM modelling, whereas in functional connectivity we may see optimal model performance with classifiers beyond SVM. Although similar results were reported along with neurophysiological dysfunction in mTBI in a binary study^30^, here we show that the same was true for other frequency ranges, and in the presence of a traumatic stress injury group with self-reported symptoms of an mTBI, as well as against two control groups (importantly, including one with exposure to traumatic stress, but having not developed PTSD).

Our multivariate learning method extracts crucial information beyond simple univariate inferential statistics for dissociating PTSD and mTBI in individual cases, even when both report symptoms consistent with a head injury. Importantly, most of the identified neural features are known to be involved in the pathophysiology of PTSD and mTBI – this is critical for building a framework that can reliably separate the two and inform treatment options with significant clinical implications, especially when it comes to designing long-term recovery programs. It is known that a minor but significant portion (~20%) of people exposed to trauma go on to develop PTSD^74^, and that roughly the same proportion of mTBI patients would continue to experience persistent post-concussive symptoms^13^. Our method could reliably identify those neural features that may well be utilised as part of symptom monitoring tool. In cases of comorbid PTSD and mTBI, failure to accurately diagnose one disorder can prevent recovery from the other^13^. As such, our findings may not only help such diagnosis, but also direct the most appropriate intervention for the PSTD+mTBI+ patients.

### Optimal classification performance is achieved separately for regional activity and interregional functional connectivity with selected features

Overall, the classification performance exhibited by the SVM models showed comparable results. We used the holdout test data to evaluate the final SVM models built with all training data. Here we identified models with optimal classification performance for both disorders.

For regional activity, the alpha and beta activities exhibited the best performance for differential classification (PTSD accuracy: 71~85%, mTBI accuracy: 85~92%). For functional connectivity, the theta, alpha and beta frequencies exhibited the best performances for both disorders (PTSD accuracy: 71~85%, mTBI accuracy: 78~85%). We also assessed the classification versatility using AUC, both the regional power and functional connectivity models at the alpha and beta models exhibited high AUC values (PTSD: ~0.8, mTBI: 0.7~0.9). With both the high classification accuracies (71%~92%) and versatility (AUC: 0.8~0.9), these models were considered the best overall models in differential PTSD/mTBI classification.

While several studies have identified the importance of “pathological” slower frequency neural oscillations (i.e. delta to theta, 1–8 Hz range) in mTBI^27,51,75^, the current results suggest faster neural oscillations (i.e. alpha to beta, 8–30 Hz) perform well in separating out one disorder from the other, even with the presence of multiple control groups, with one of those exposed to traumatic stress. In fact, alpha and beta activity is to be atypical in PTSD^76^ and mTBI^77^, as well as in individuals diagnosed with both conditions^78^. Alpha oscillations are thought to reflect regional ‘gating-through-inhibition’^79^ and beta oscillations are reliable markers of cortical inhibition^80^, suggesting these disorders can be characterised oscillatory dynamics that index pathological disinhibition and/or dysregulated excitation (or a combination of both) – this has certainly been reported in both mTBI^81–83^ and PTSD^84,85^ in human and animal studies. Mechanistically, the role of beta oscillations varies by region but is important in somatosensory processing and cortical coupling^86^, with a relevant feature bihemispheric post-central gyrus features selected here (Fig. 3). Beta activity in frontal cortices is implicated in memory and executive functioning^87^, consistent with the most selected feature in terms of regional power (Fig. 3).

These results demonstrate that the selected features hold the promising potential of PTSD/mTBI diagnosis and hold important significant clinical implications. For example, these models can potentially be used to detect the development of PTSD for patients recovering from concussion, allowing appropriate treatment planning^13,88^.

### Limitations and conclusions

As a follow-up to our previous standalone PTSD and mTBI reports^29,30^, the current study combines the cohorts featured in those studies. Therefore, one major limitation is the male only cohorts. Given the prevalence of both the diseases in female (especially in the case of PTSD)^89–91^, it is our major future direction to explore differential PTSD/mTBI diagnosis in a sex-balanced or female-only fashion. Indeed, the present study established a MEG- and multivariate feature selection-based framework for these upcoming studies. Additionally, our models can be further refined with the addition of more case data.

This study demonstrated the viability of combining measures of neural activity with a feature selection system for understanding the neurobiology of, and differentially identifying, cases of PTSD and mTBI cases, even with concurrent symptoms overlap. Overall, our multiclass feature selection objectively identifies the most relevant features and the best models for differential PTSD/mTBI classification, without the need of any a priori knowledge of the specific brain circuits involved. Therefore, differential diagnosis of PTSD from mTBI based on the selected features appears highly realistic and beneficial; and being able to accurately classify individuals who are PTSD+mTBI+ from those who are either PTSD−mTBI+ or mTBI−PTSD+ would be the critical next step. Additionally, multiclass classification with data integrated across frequencies, rather just within (i.e. band-limited oscillations), has now been shown to be a powerful method in a binary classification context for mTBI^92^. Moreover, the application of the multivariate statistical learning algorithms promises to better classify heterogeneous patient populations presenting with unique symptoms, as well as predict individual responses to treatment, in order to facilitate a personalised therapeutic approach to brain disorders^93^. Collectively, these findings reveal the promising potential of combining ‘invisible’ neurophysiological indices of brain function with machine learning to address the significant health challenges posed by these debilitating conditions.

## Supplementary information

Supplmentary methods and results

Supplementary figure and table legends

Supplementary figures S1-S11

Table S1

Table S2. Complete univariate analysis results for AEC.

Table S3

Table S4

## Data Availability

Software available via github: https://github.com/jzhangc/git_meg_ml_app. Project code can be requested via corresponding author.
