# Supplementary material for: Teasing apart trauma: neural oscillations differentiate individual cases of mild traumatic brain injury from post-traumatic stress disorder even when symptoms overlap
Source: Transl Psychiatry. 2021 Jun 4;11:345. doi: 10.1038/s41398-021-01467-8 (PMC8178364; doi:10.1038/s41398-021-01467-8)
Supplement: Supplementary file 1 — Supplmentary methods and results [file 41398_2021_1467_MOESM1_ESM.docx]

**Supplementary methods**

BrainNet Viewer, as well as R packages ggplot2, gplots (https://CRAN.R-project.org/package=gplots) and RBioplot, were used for data and results visualization ^1–3^.

***Diagnoses and inclusion criteria***

The inclusion criteria applied to all four participant groups: English-speaking and capable of understanding instructions and giving informed consent; no contra-indications to MRI and MEG scanning.

PTSD was diagnosed by a psychiatrist or psychologist specialized in trauma-related mental health injuries using a comprehensive, semi-structured interview based on the diagnostic criterion of the DSM-IV-TR. Additionally, the CAF-standardized psychometric test was also used during the process. The PTSD participants were subject to comprehensive initial inclusion criteria, including the presence of PTSD symptoms for 1-4 years before the study and regular mental health follow-up. The participants were also subject to the Defense and Veteran’s Brain Injury Centre (DVBIC)’s three-item screening tool as a screener to confirm no history of TBI. Extensive exclusion criteria included any history of seizures and other neurological disorders, as well as active substance abuse. Since all PTSD participants were considered naturalistic samples, they were undergoing treatment with one or more evidence-based psychotropic medications, such as selective reuptake inhibitors (SSRIs), serotonin-norepinephrine reuptake inhibitors (SNRIs) and Prazosin.

For the mTBI group, the inclusion criteria included a positive mTBI diagnosis made by a clinician specialising in head trauma. MRI images were read by a neurosurgeon specialising in head trauma to confirm negative MR. MEG imaging and symptom assessment was conducted within three months (≤90 days) post-injury. Exclusion criteria featured any conditions that could also interfere with MRI screening, medical device implantation, seizures or other neurological disorders, active substance abuse, as well as certain ongoing medications known to modulate neurophysiological signatures. mTBI patients with PTSD diagnosis were also excluded.

***Magnetoencephalography***

5-minute eyes open resting state MEG scans (600 Hz) with participants in the supine position were collected on a CTF system with 151 channels at the Hospital for Sick Children (Toronto, ON, Canada). The data were analyzed using the FieldTrip Matlab toolbox ^4^. Data were then co-registered with each individual anatomical T1 MRI using fiducial markers on the MRI. A linearly constrained minimum variance (LCMV) vector beamformer ^5^ was used to recover time series from the 90 brain regions defined by the Automated Anatomical Labelling atlas (AAL) centroid ^6^, resulting in one-dimensional activity time series for each node. Z-score transformation was used for each node time series.

The signal was bandpass filtered offline with a high-pass filter of 1Hz and a low-pass filter of 150 Hz, with a 60 Hz notch filter. Artifacts were removed using independent component analysis (ICA) within Fieldtrip (‘fastica’). Continuous data were epoched into thirty 10 second epochs, with the maximum number of epochs selected using the following 2 conditions: 1. For each epoch, the head position deviated less than 8 mm from the recoding median; 2. Epochs were excluded for those with SQUID resets or exceeding a threshold of ±2 pT after ICA component rejection.

Lead fields were determined from the subject single-shell head model for a unit current dipole in three dimensions at each node. For each node, the beamformer weights were calculated for the activity via projecting the sensor weights along the axis with the highest singular value decomposition (SVD) variance.

The band filtered data were submitted to symmetric orthogonalization to attenuate inflated connectivity/coupling due to beamformer leakage ^7,8^. The filtered and orthogonalized time series data for each node were Hilbert transformed to derive instantaneous estimates of the amplitude envelope. The amplitude envelop was then down sampled to 1 Hz by averaging over 1s intervals and Pearson correlations run between all node pairs to index functional connectivity.

***Univariate analysis***

Univariate analysis was based on the multiclass (i.e. multiple groups) linear modelling with empirical Bayesian moderation, as described previously ^9^. Due to the multiclass nature of the current study, the overall statistics were determined by the moderated F-statistics. Features were filtered based on the p-values calculated from the moderated F-statistics. The reduced features were then used as the basis to determine the effectiveness of capturing group differences, via unsupervised clustering analysis. Moreover, two-group contrasts were constructed and assessed. Specifically, four comparisons were extracted from the overall statistical analysis: PTSD vs TC, mTBI vs NTC, PTSD vs mTBI, and TC vs NTC. For each contrast, features passing the p-value threshold were identified.

P-value thresholding was conducted on both the raw and FDR (false positive rate)-corrected p-values. Firstly, thresholding on raw p-value was for ML univariate feature reduction check. Since any false positive features were ultimately addressed by rRF-FS, raw p-value thresholding ensured to preserve maximum data variance whilst reducing the data size. Due to the different data properties presented by regional power and AEC, the p-value threshold values were adjusted dynamically for this stage. Secondly, thresholding on FDR-corrected p values (alpha=0.05) was also carried out to assess group difference. Since no further feature reduction was conducted for this purpose, false positive features were addressed via FDR. For the current study, only the features passing the threshold with the FDR-corrected p-value were considered statistically significant.

Hierarchical clustering was used on both the complete data and univariate feature reduced data (from two-group comparisons) to assess the overall data property and the group differences as represented by the univariate reduced features, respectively. Additionally, both complete and univariate feature reduced data were subject to principal component analysis (PCA), which examined group difference and data complexity.

***Support vector machine with cross validation feature selection***

Due to the multiclass nature, both SVM and PLS-DA modelling followed the “One vs All” approach. Data scaling and standardization were only conducted using the training data information during both the CV and final modelling stages. Although the number of participants per group showed small levels of variance, class weights were still applied to during modelling to mitigate the slightly imbalanced data.

For nested CV, the training data was segmented into 10 data folds with the same stratified resampling method. With the training data segmented, the training data were grouped into 10 “CV training and CV test data” combinations, with each combination containing 9 data fold as the CV training data and the renaming fold as the CV test data. Each CV-SVM-rRF-FS iteration generated a list of selected features and a CV SVM model. The selected feature lists were then combined to generate a consensus feature list through a simple voting step, where features selected at least two times were retained.

For each frequency, a brain net AAL map was generated as a visual representation of the ML selected features. The CV SVM models were then assessed for classification performances. The selected features were also verified via PLS-DA for their modelling generalizability. A final SVM model was generated with the final consensus feature list.

For permutation tests, the PLS-DA models were tested with 999 permutation iteration, whereas 99 iterations were used for SVM models due to computational resources restrictions.

Additionally, SVM classifiers were generated with mTBI symptom and severity scores, with radial kernel. No feature selection was conducted as these models were single feature models. 10-fold CV was also used to optimize parameters. The models were assessed with 15% holdout test data.

The R implementation of the popular SVM library libsvm was used for the core SVM modelling steps (e1071 package, https://CRAN.R-project.org/package=e1071). The R package RBioFS was used for PLS-DA modelling ^10^.

***Additional statistical analysis***

Kruskal-Wallis ANOVA (analysis of variance) with Tukey post-hoc test was used to assess the mTBI symptoms, severity (Sports Concussion Assessment Tool 2, or SCAT2)^11^, anxiety (Generalized Anxiety Disorder 7, or GAD-7) ^12^ and depression (Patient Health Questionnaire, or PHQ-9) ^13^ between the PTSD and mTBI groups, along with the control groups. One-Way ANOVA test was used to compare per group classification accuracy and AUC values across seven frequency bands and both types of neural functioning (regional activity and functional connectivity). Changes with a *p*-value less than 0.05 were considered statistically significant. ANOVA and Tukey tests were conducted using the R package PMCMR (<https://CRAN.R-project.org/package=PMCMR>) and R native functions. Additionally, due to the nature of these compassions (i.e. four comparisons of varying feature types), FDR correction was not used.

**Supplementary results**

***Contrasting group differences using a univariate statistical analysis***

Score plots were used to present PCA results (**Fig. S4**). It is noted that volcano distribution was used when not enough features were identified for clustering (**Fig. S3D**).

***Machine learning feature selection identifies the most relevant features for modelling***

*Univariate reduction*

For the univariate reduction, we tested alpha values of 0.05 and 0.01 for both feature types.

For the regional power data, alpha=0.01 showed too few features, therefore alpha of 0.05 were used for the analysis. For the AEC data, however, when set to 0.05, there were still a substantial number of features that drastically slowed down the analysis, with no apparent ML performance gain when comparing to thresholding at 0.01. So, the ML analysis was run with the univariate reduced features (alpha=0.01), instead of the full 4005 features.

With the univariate reduction (alpha=0.05) for the regional power data, for all seven frequency bands and based on the moderated F-statistics, less than 15 regions were identified across the four participant groups (**Table. S2**). Deriving from these cross-group results, it is not a surprise that the two-group comparisons identified similar number of brain regions that passing the p-value threshold for all seven frequency bands. More importantly, as shown in **Fig. S2 and S4A**, both the hierarchical clustering and PCA results on only the univariate reduced brain regions failed to generate participant clusters according to the participant groups of interest. These results suggested that univariate feature reduction might exclude crucial information for ML classification modelling. Therefore, the ML was conducted with the full 90 features for the regional power data.

For the AEC univariate reduction, the cross-group statistics exhibited 48, 54, 80, 70, 43, 46 and 26 functional edges passing the p-value threshold for the delta, theta, alpha, beta, low gamma one, low gamma two and high gamma frequencies, respectively, down from the total number of 4005 edges (**Table S3**,). The moderated F-statistics also led to similar numbers of functional edges passing the p-value threshold for the two-group contrasts. The univariate reduced features led to two major clusters clearly corresponding to the two participant groups of interest shown by hierarchical clustering and PCA, for all seven frequency bands tested. **Fig. 2C and 2D** is an example showing the clustering results for the “PTSD vs mTBI” comparison for the alpha and low gamma one bands. Moreover, PCA results on the identified functional edges for the same frequency band exhibited group separation over three principal components (**Fig. 4A**). The remaining clustering results can be found in **Fig. S3** and **Fig. S4B**.

As such, with only the training data, ML analysis started with the full 90 brain regions for the regional power data (i.e. one-step feature selection), whereas functional connectivity data underwent ML analysis with the initial univariate reduction during each nested CV (cross validation) iterations (i.e. two-step feature selection).

*Feature selection*

The delta band AEC identified connections like the left median cingulate and paracingulate gyri-to-right thalamus, right thalamus-to-right postcentral gyrus, right thalamus-to-right postcentral gyrus, right transverse temporal gyrus-to-left inferior parietal lobule as well as right transverse temporal gyrus-to-left lenticular nucleus (pallidum). Theta band AEC features included left parahippocampus-to-right amygdala, and right transverse temporal gyrus-to-right superior temporal pole. For the alpha band AEC, the left postcentral gyrus-to-right postcentral gyrus connection was selected, which was the statistically significant feature identified by univariate analysis. In the low gamma one band (30-55 Hz) functional connections involving the left superior frontal gyrus-to-right thalamus, right superior frontal gyrus-to-right transverse temporal gyrus, and the left transverse temporal gyrus-to-right middle occipital gyrus were selected, and for low gamma two (65-80 Hz), the right precentral gyrus-to-right hippocampus. High gamma band AEC identified the functional edges of the right thalamus to-right transverse temporal gyrus and the left hippocampus-to-left amygdala.

For both feature types, a PLS-DA model were generated using the corresponding consensus feature list in order to test the model generalizability of the selected features. In general, the PLS-DA modelling failed to generate significant models for multiclass classification with the reginal power data (**Fig. S5**). For the AEC PLS-DA models, however, the permutation tests suggested all PLS-DA models were significant in classification. The complete PLS-DA model evaluation results for the AEC data can be viewed in **Fig. S6**.

***Machine learning multiclass classification modelling reaches optimal performance***

Upon initial testing, for the regional power data, the linear kernel was used for all the frequency bands tested. Regarding the AEC data, the radial kernel was used for the low gamma one band, with the rest utilizing the linear kernel. Regarding ROC-AUC analysis, no statistically significant differences were observed across the seven frequency bands for both data types for the CV models (**Fig. S7**). For the final models, the permutation test results showed that all final SVM models (i.e. all seven frequency bands, and both data types) were significant (permutation p<0.05). The complete SVM permutation test results can be viewed in **Figs. S8** and **S9**.

In terms of the regional power data, all but two (theta and low gamma two) CV models reached or surpassed 70% (mean value) accuracy in classifying the PTSD group (**Table 1A**). For mTBI classification, all but the alpha model reached 60% (mean±SD) accuracy (**Table 1A**). Similarly, the AUC values also reached at around 0.7 (mean±SD) for classifying PTSD for all the frequency bands (**Table S4A**). Moreover, the CV model performances either reached or surpassed AUC of 0.6 (mean value) for mTBI classification as well.

For the functional connectivity data, as shown in **Table 1A**, the CV models exhibiting the highest classification accuracy for PTSD were derived from the alpha and beta bands (mean accuracy around 75%). For mTBI classification, the highest CV performance was shown at the beta frequency (73.72±7.93%), which was closely followed by the low gamma one, alpha and theta bands. **Table S4B** includes the AUC values for the CV models at each frequency band. For PTSD classification, all frequency band exhibited an AUC value approaching or surpassing 0.7 (mean). The mTBI classification exhibited comparative results as the PTSD performance.

**Supplementary references**

1. Xia, M., Wang, J. & He, Y. BrainNet Viewer: A network visualization tool for human brain connectomics. *PLoS One* **8**, e68910 (2013).

2. Wickham, H. *ggplot2: Elegant graphics for data analysis*. (Springer-Verlag New York, 2016).

3. Zhang, J. & Storey, K. B. RBioplot: an easy-to-use R pipeline for automated statistical analysis and data visualization in molecular biology and biochemistry. *PeerJ* **4**, e2436 (2016).

4. Oostenveld, R., Fries, P., Maris, E. & Schoffelen, J. M. FieldTrip: Open source software for advanced analysis of MEG, EEG, and invasive electrophysiological data. *Comput. Intell. Neurosci.* **2011**, (2011).

5. Van Veen, B. D., van Drongelen, W., Yuchtman, M. & Suzuki, a. Localization of brain electrical activity via linearly constrained minimum variance spatial filtering. *IEEE Trans. Biomed. Eng.* **44**, 867–880 (1997).

6. Tzourio-Mazoyer, N. *et al.* Automated anatomical labeling of activations in SPM using a macroscopic anatomical parcellation of the MNI MRI single-subject brain. *Neuroimage* **15**, 273–289 (2002).

7. Colclough, G. L. *et al.* How reliable are MEG resting-state connectivity metrics? *Neuroimage* **138**, 284–293 (2016).

8. Colclough, G. L., Brookes, M. J., Smith, S. M. & Woolrich, M. W. A symmetric multivariate leakage correction for MEG connectomes. *Neuroimage* **117**, 439–448 (2015).

9. Ritchie, M. E. *et al.* limma powers differential expression analyses for RNA-sequencing and microarray studies. *Nucleic Acids Res.* **43**, e47 (2015).

10. Zhang, J., Hadj-Moussa, H. & Storey, K. B. Current progress of high-throughput microRNA differential expression analysis and random forest gene selection for model and non-model systems: an R implementation. *J. Integr. Bioinform.* **13**, 306 (2016).

11. SCAT2. *Br. J. Sports Med.* **43**, i85 LP-i88 (2009).

12. Spitzer, R. L., Kroenke, K., Williams, J. B. W. & Löwe, B. A brief measure for assessing generalized anxiety disorder: the GAD-7. *Arch. Intern. Med.* **166**, 1092–1097 (2006).

13. Kroenke, K., Spitzer, R. L. & Williams, J. B. The PHQ-9: validity of a brief depression severity measure. *J. Gen. Intern. Med.* **16**, 606–613 (2001).
