# Supplementary material for: Teasing apart trauma: neural oscillations differentiate individual cases of mild traumatic brain injury from post-traumatic stress disorder even when symptoms overlap
Source: Transl Psychiatry. 2021 Jun 4;11:345. doi: 10.1038/s41398-021-01467-8 (PMC8178364; doi:10.1038/s41398-021-01467-8)
Supplement: Supplementary file 2 — Supplementary figure and table legends [file 41398_2021_1467_MOESM2_ESM.docx]

**Supplementary figure legends**

Figure S1. Unsupervised hierarchical clustering heatmaps on (A) the complete regional power data, (B) the complete functional connectivity data.

Figure S2. Unsupervised hierarchical clustering heatmaps on the univariate feature reduced regional power data.

Figure S3. Unsupervised hierarchical clustering heatmaps on the univariate feature reduced functional connectivity data.

Figure S4. PCA score plot for the complete and univariate feature reduced data. (A) regional power, (B) functional connectivity.

Figure S5. PLS-DA permutation results for the regional power models.

Figure S6. PLS-DA permutation results for the AEC models.

Figure S7. Bar graphs showing AUC for CV models comparing all seven frequency bands. Bars±error bars are mean±SD. Different letters or letter combinations represent statistically significant performance differences. (A) regional power, (B) AEC.

Figure S8. Permutation test results for final SVM models for the regional power data.

Figure S9. Permutation test results for final SVM models for the functional connectivity data.

Figure S10. ROC-AUC results for final SVM models for the regional power data.

Figure S11. ROC-AUC results for final SVM models for the functional connectivity data.

**Supplementary table legends**

**Table S1.** Complete univariate analysis results for regional power.

**Table S2.** Complete univariate analysis results for AEC.

**Table S3**. Consensus feature list from machine learning feature selection.

**Table S4.** SVM modelling CV AUC.
