# Supplementary material for: Teasing apart trauma: neural oscillations differentiate individual cases of mild traumatic brain injury from post-traumatic stress disorder even when symptoms overlap
Source: Transl Psychiatry. 2021 Jun 4;11:345. doi: 10.1038/s41398-021-01467-8 (PMC8178364; doi:10.1038/s41398-021-01467-8)

Fig. S1A – full hierarchical: power

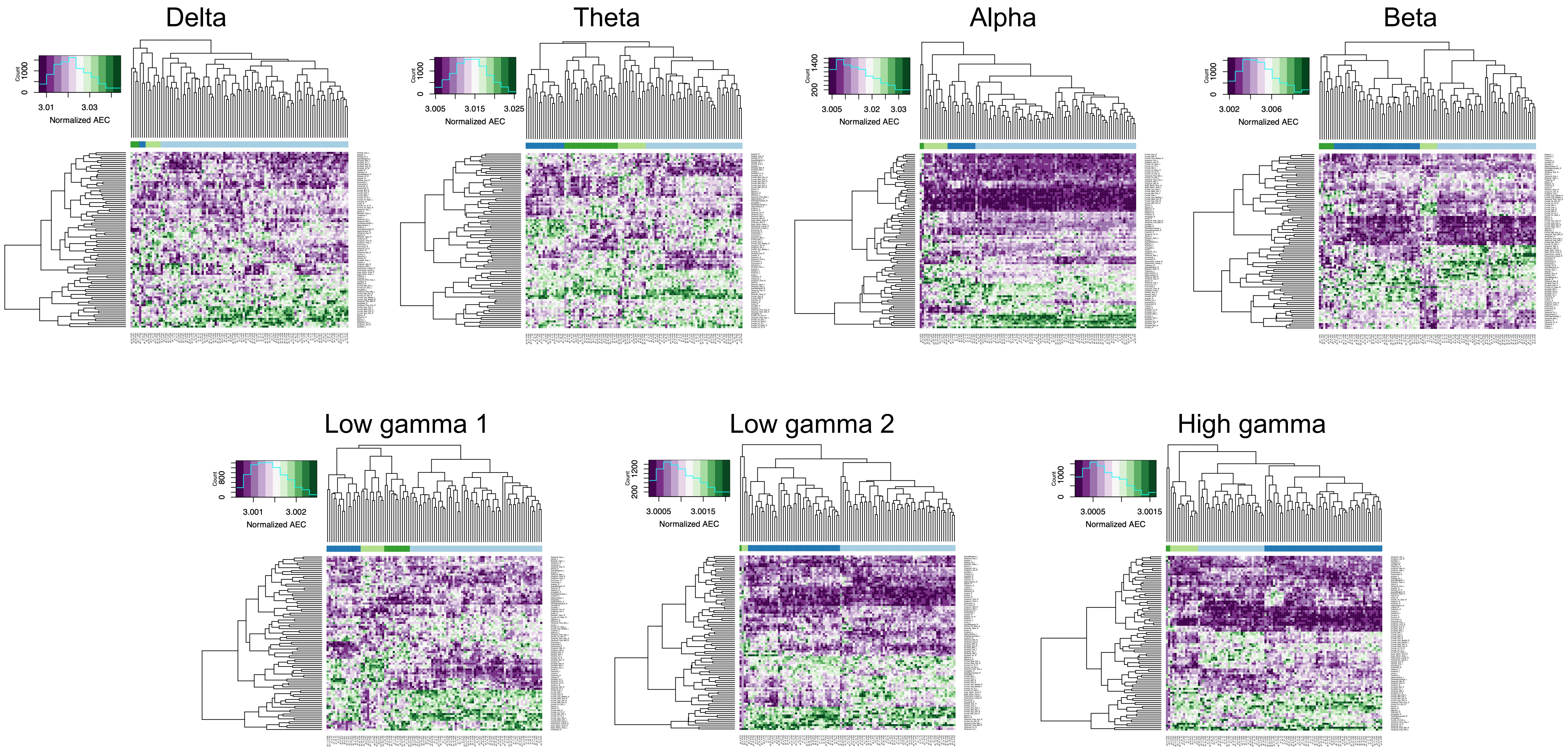

Fig. S1B – full hierarchical: AEC

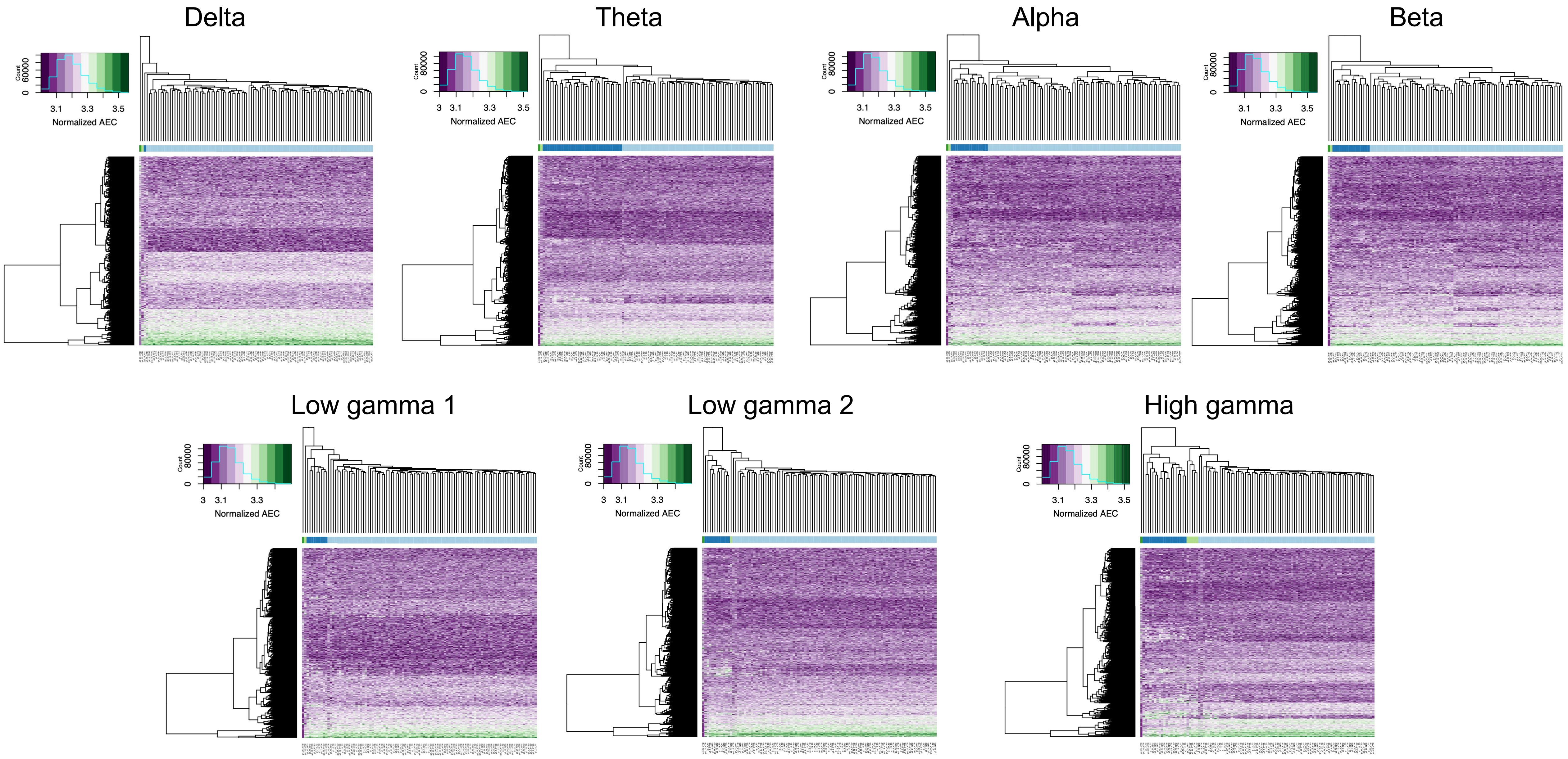

Fig. S2A hierarchical significant results: power – Delta

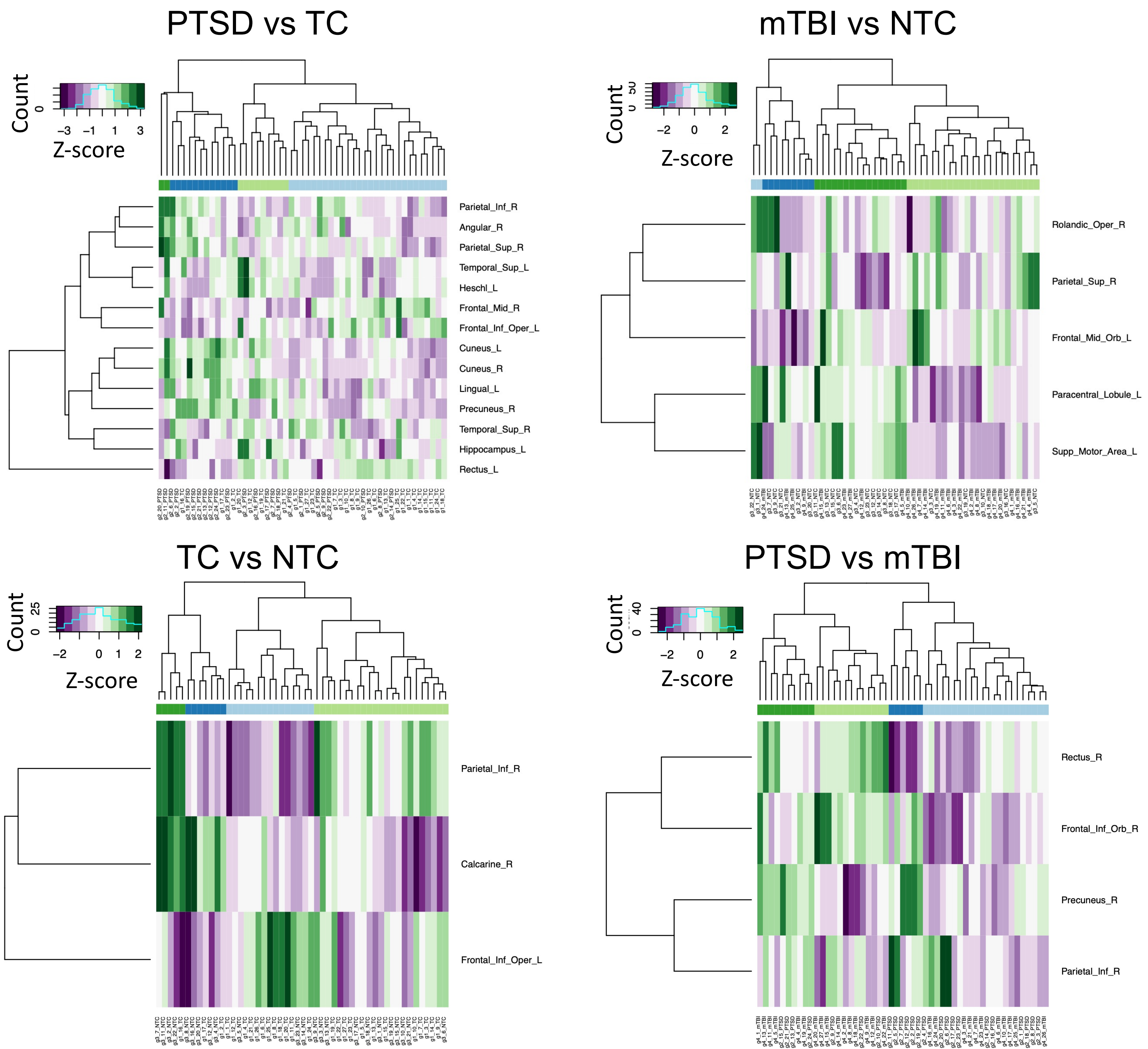

Fig. S2B hierarchical significant results: power – Theta

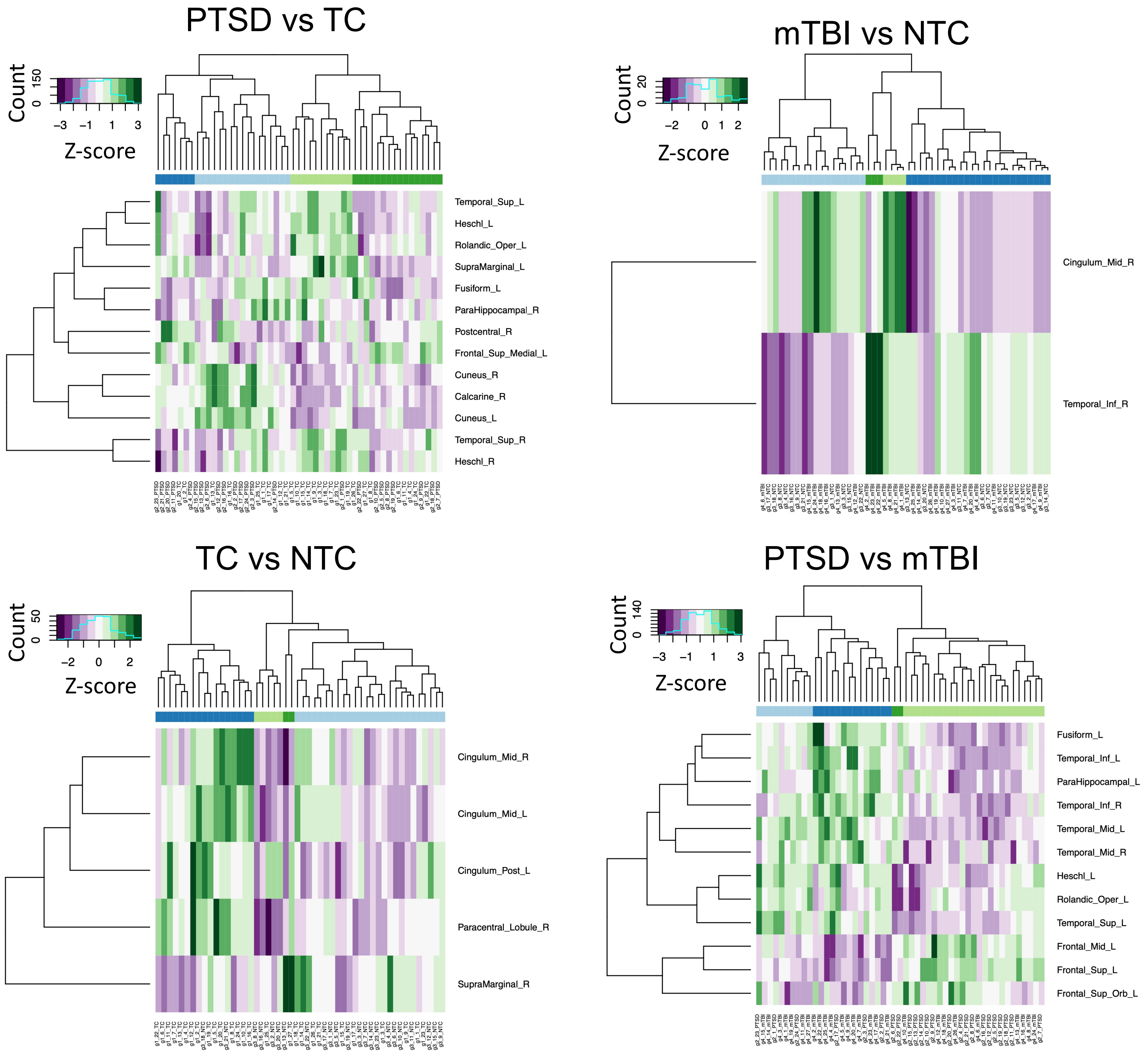

Fig. S2C hierarchical significant results: power – Alpha

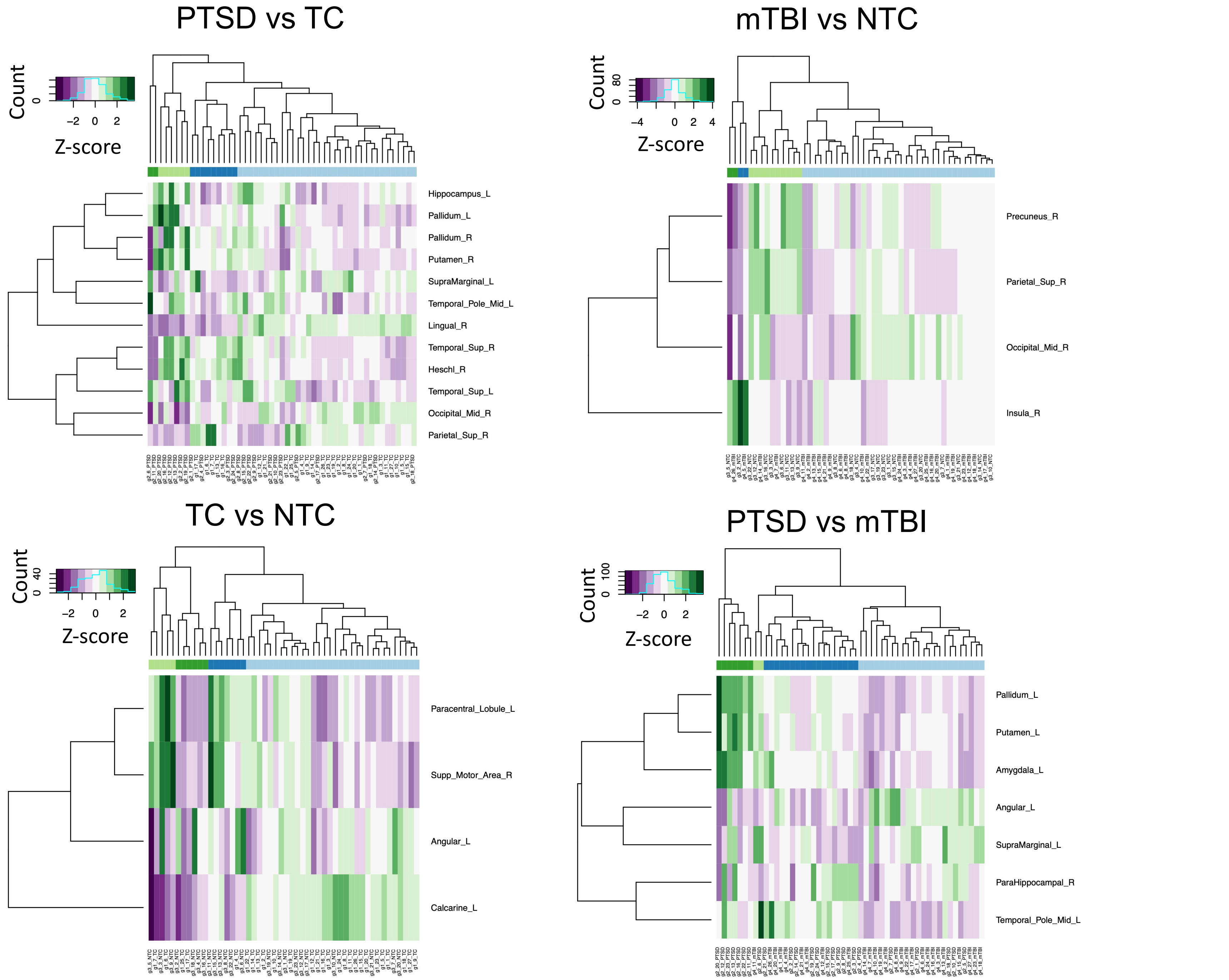

Fig. S2D hierarchical significant results: power – Beta

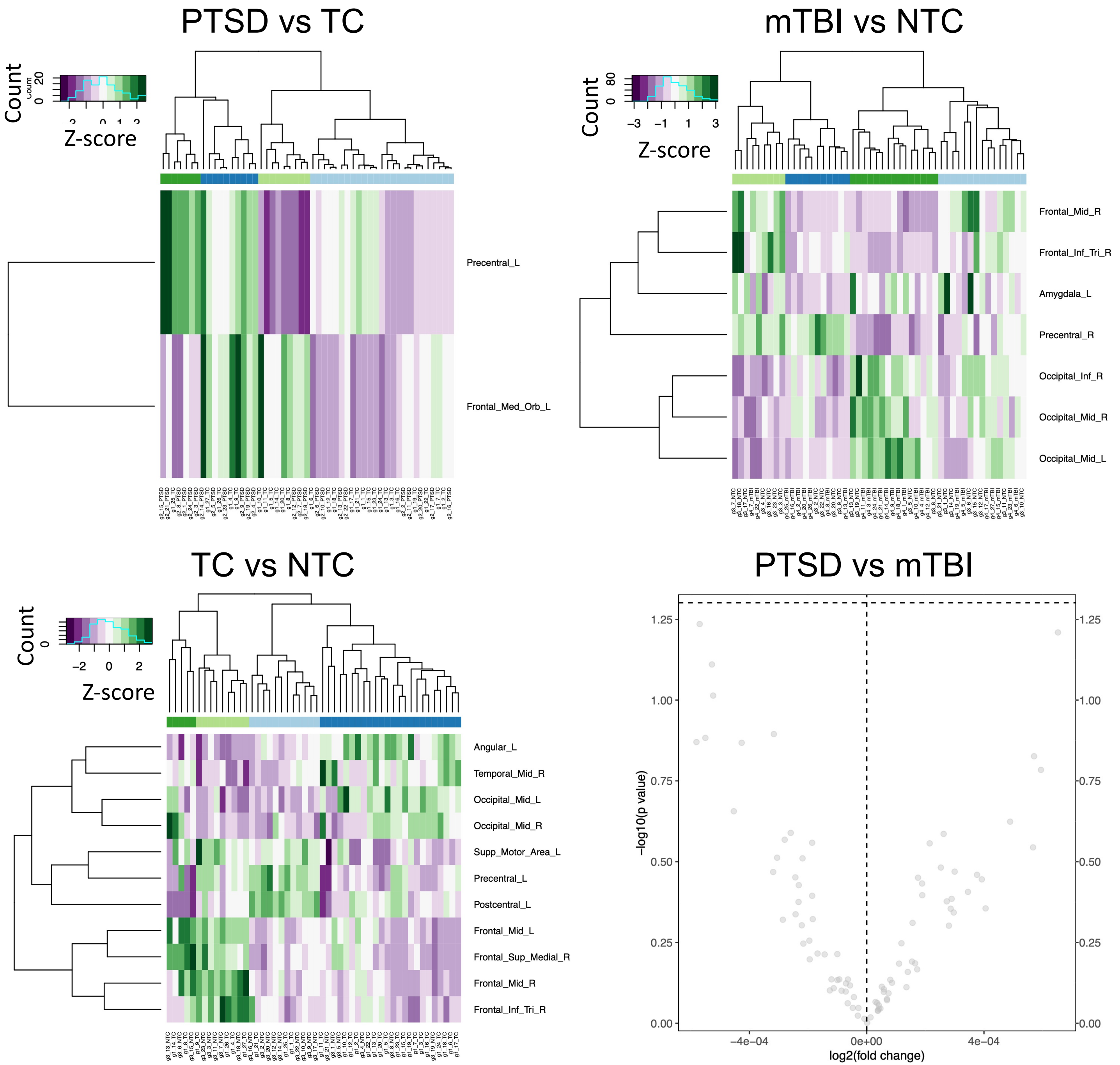

Fig. S2E hierarchical significant results: power – Low gamma one

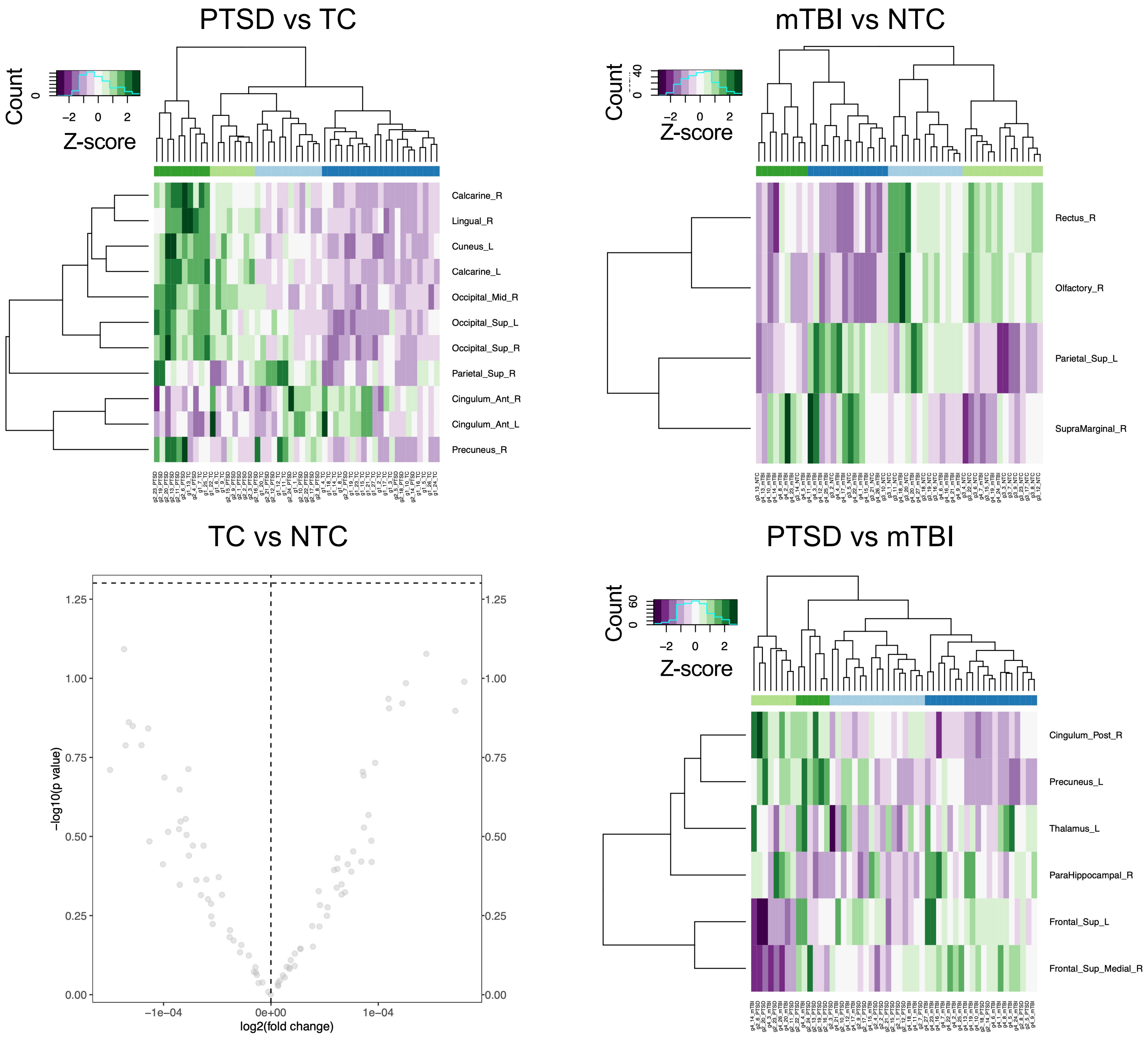

Fig. S2F hierarchical significant results: power – Low gamma two

PTSD vs TC

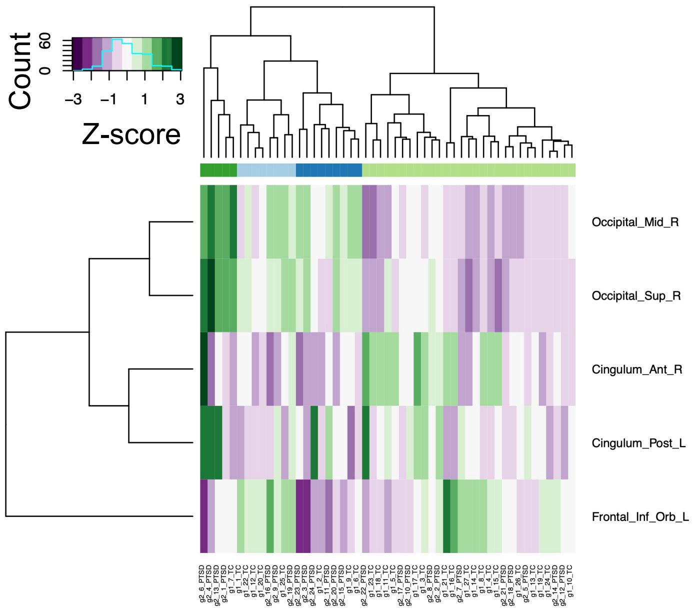

mTBI vs NTC

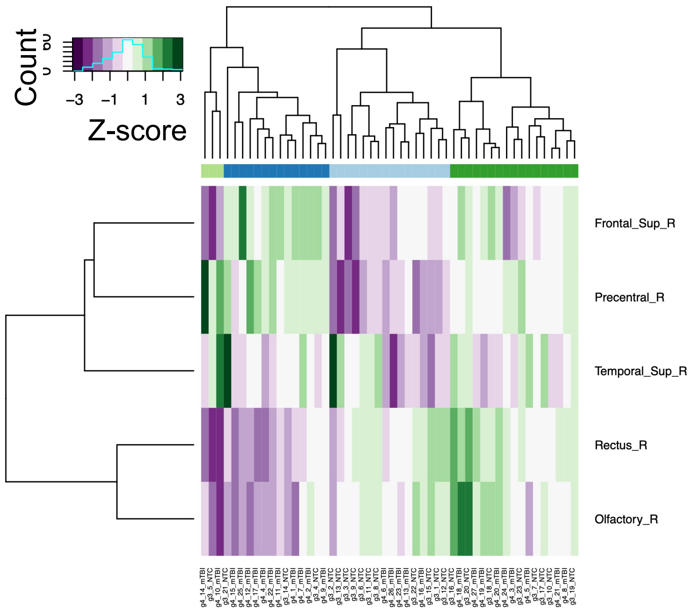

TC vs NTC

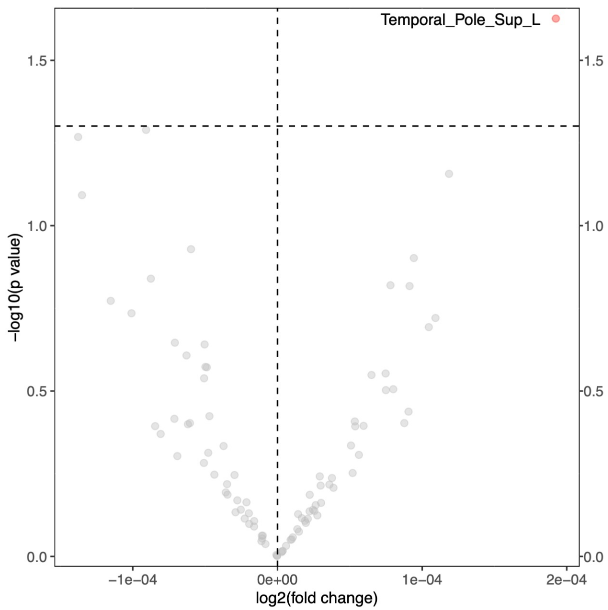

PTSD vs mTBI

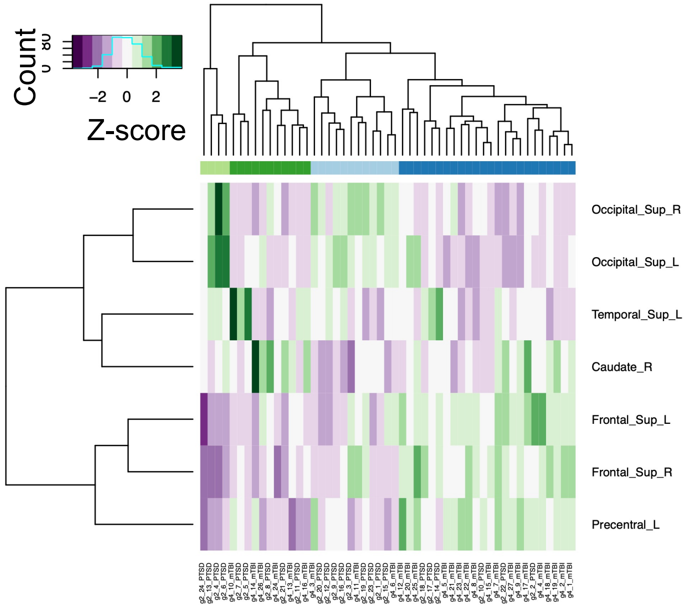

Fig. S2G hierarchical significant results: power – High gamma

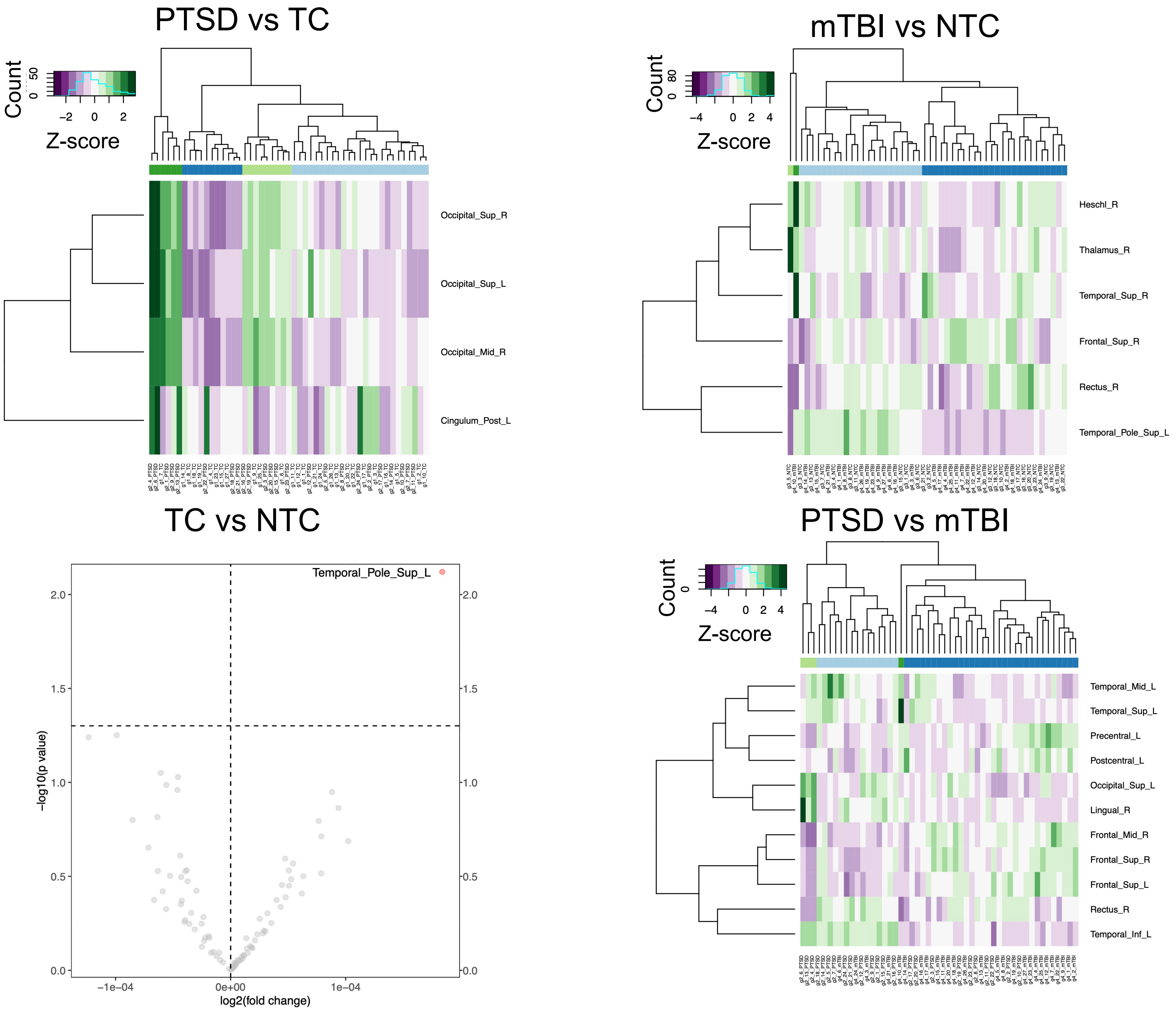

Fig. S3A hierarchical significant results: AEC – Delta

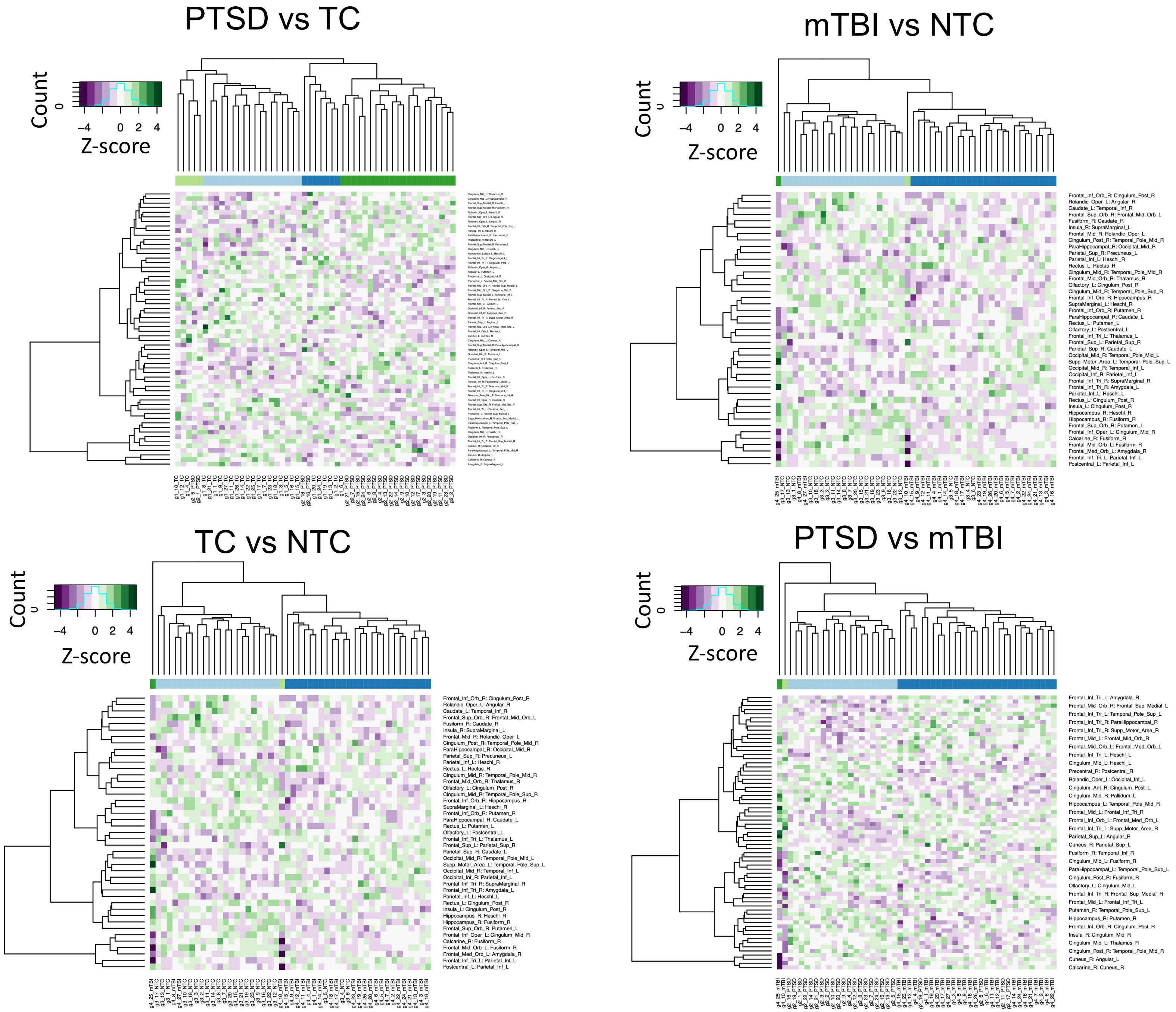

Fig. S3B hierarchical significant results: AEC – Theta

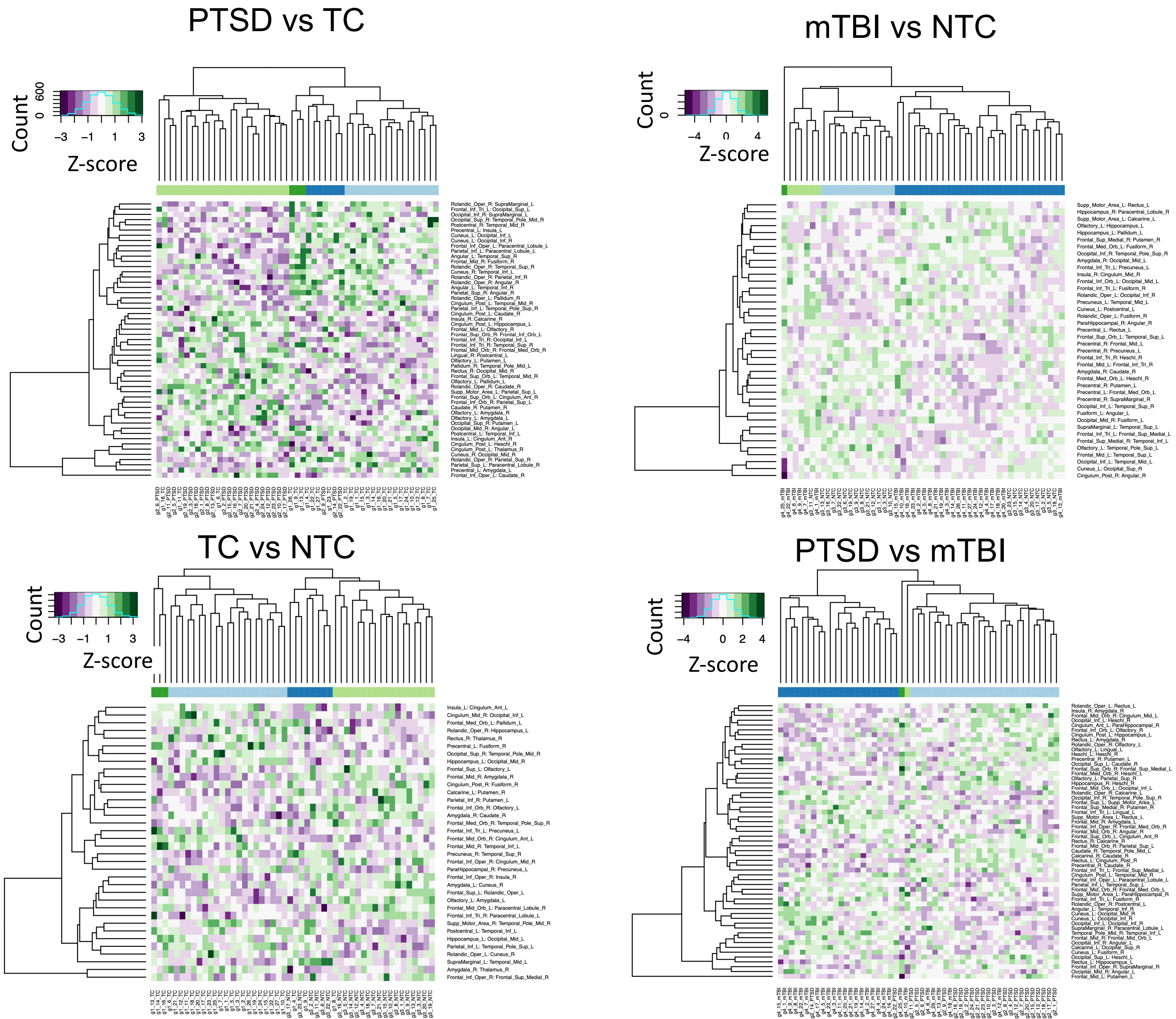

Fig. S3B hierarchical significant results: AEC – Alpha

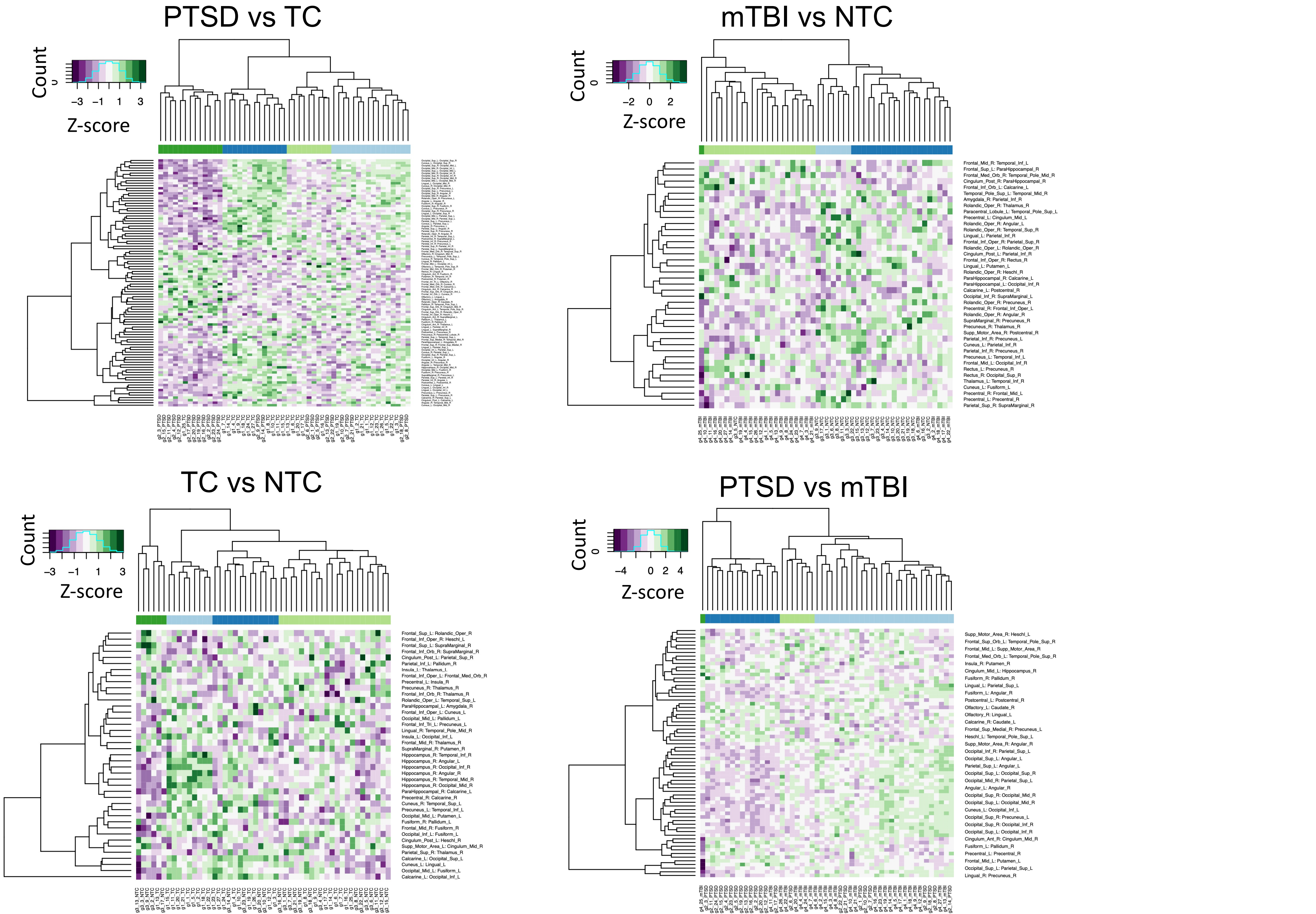

Fig. S3C hierarchical significant results: AEC – Beta

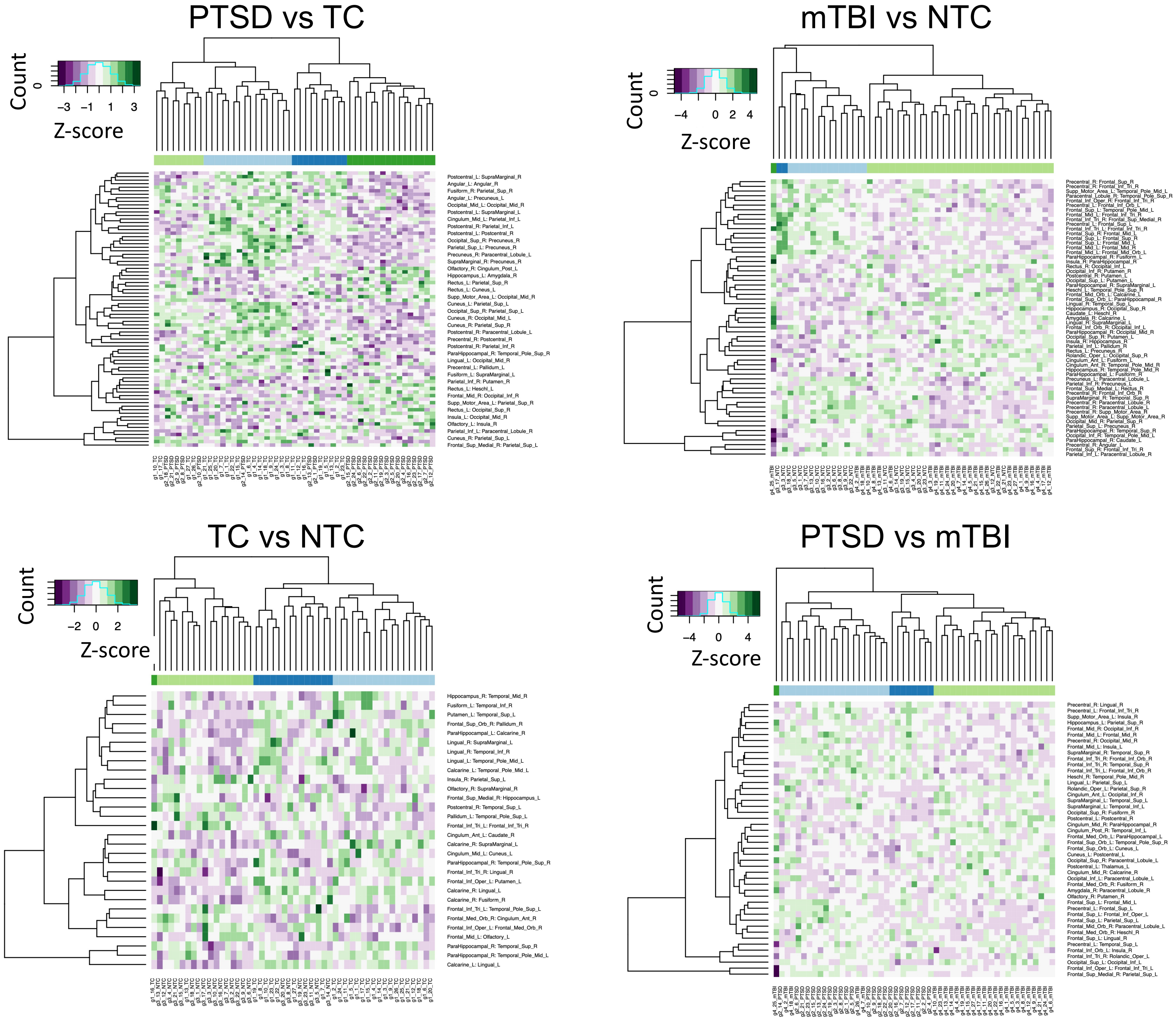

Fig. S3D hierarchical significant results: AEC – Low gamma one

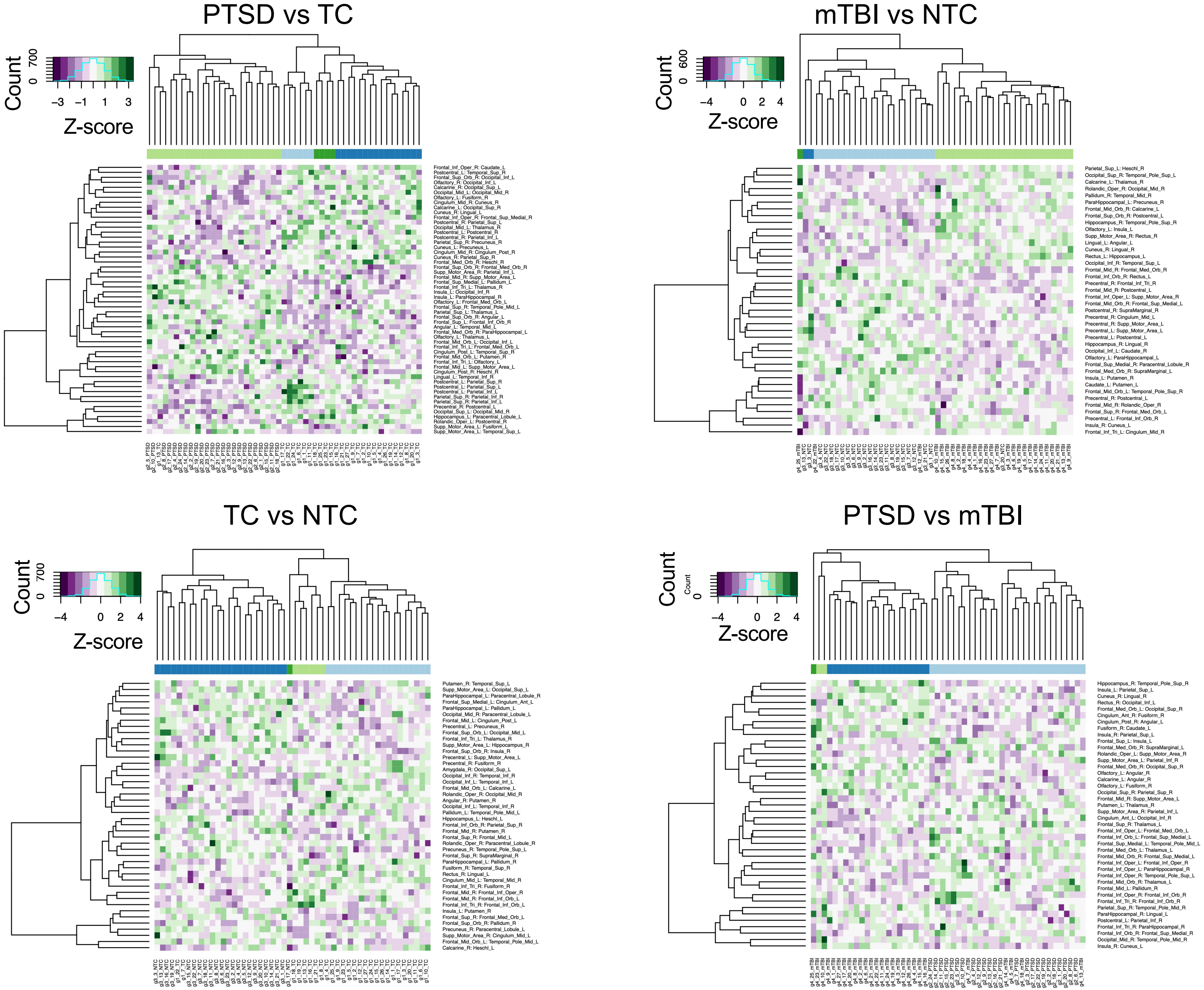

Fig. S3E hierarchical significant results: AEC – Low gamma two

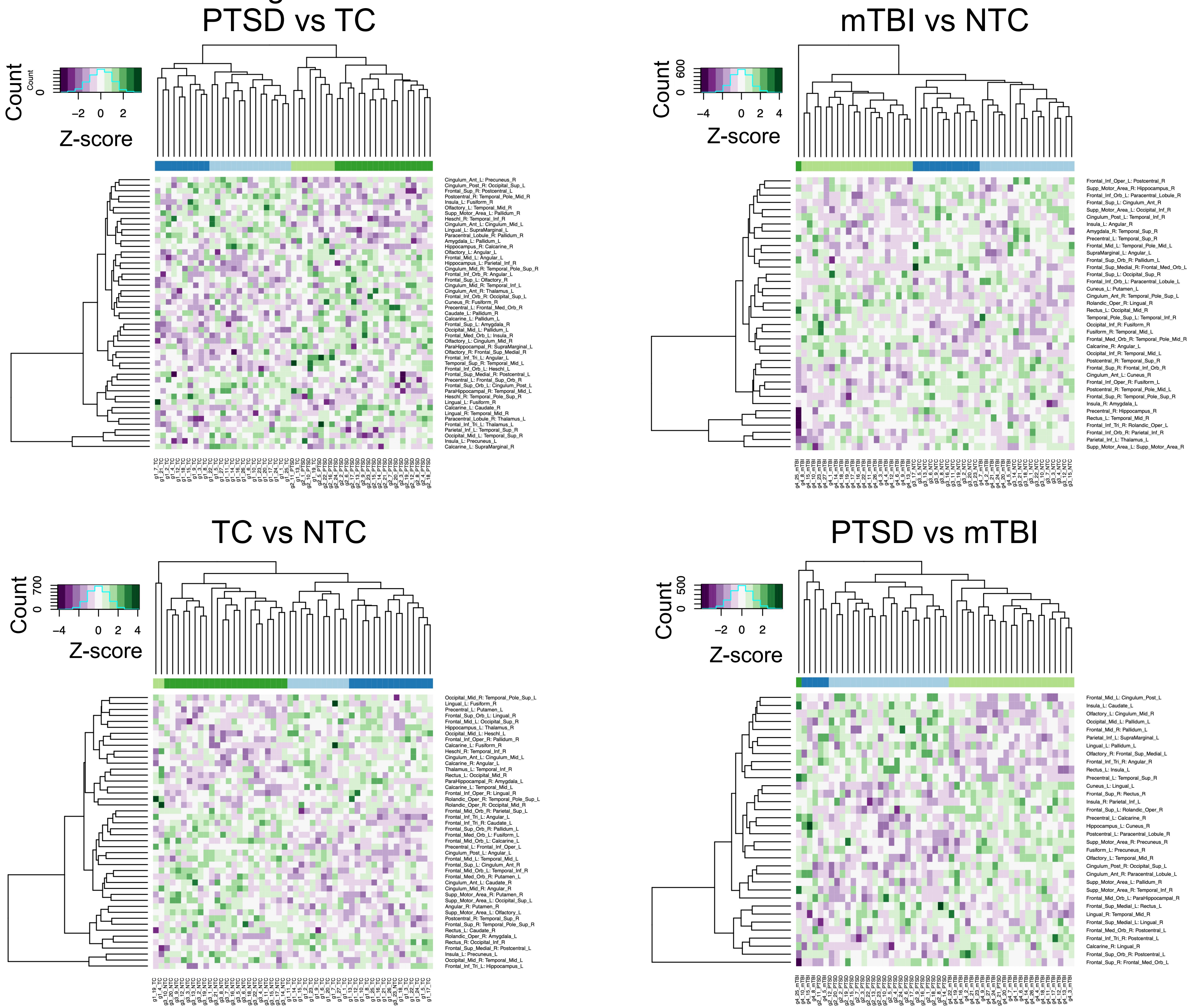

Fig. S3F hierarchical significant results: AEC – High gamma

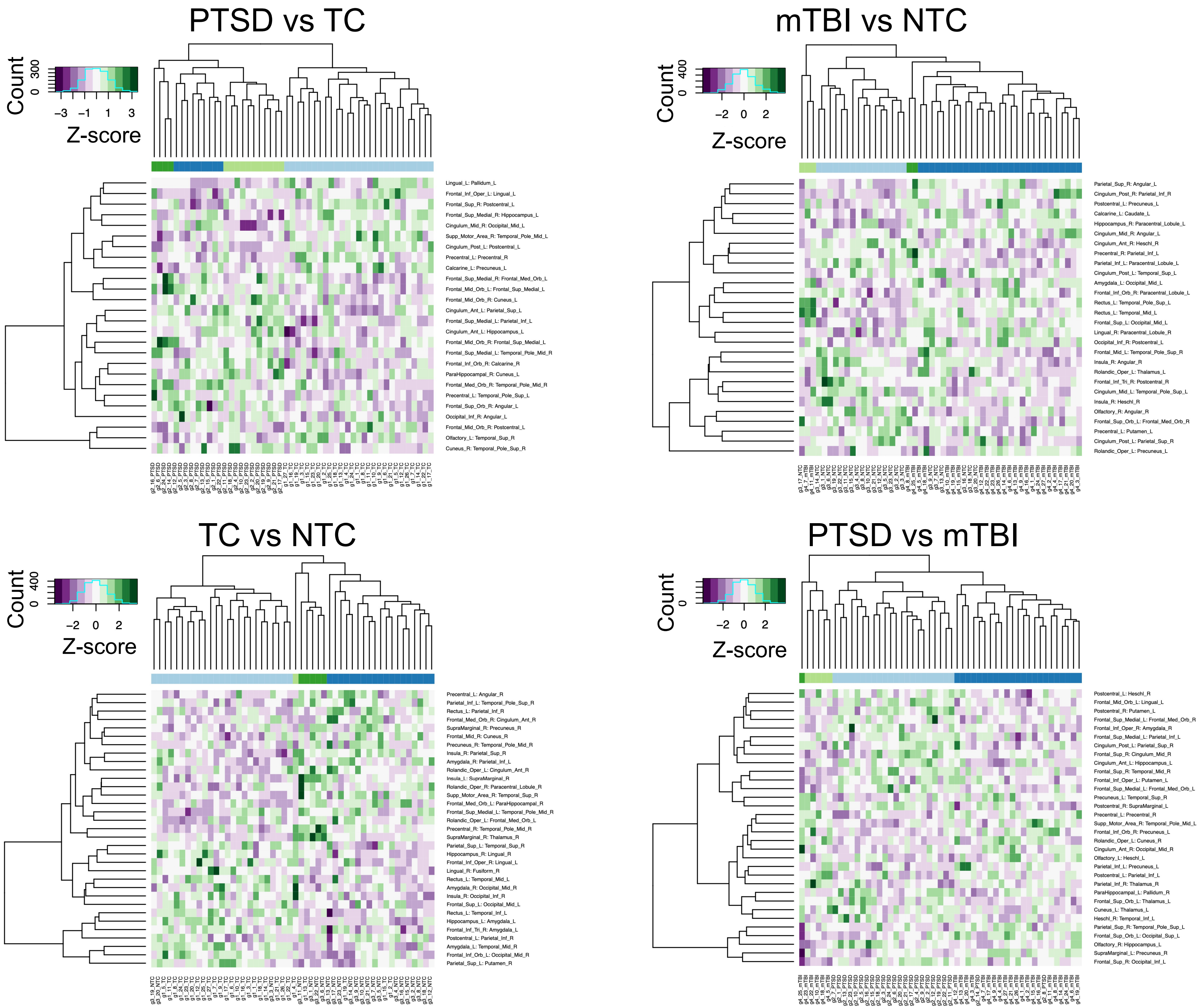

Fig. S4A. Power: PCA score plot

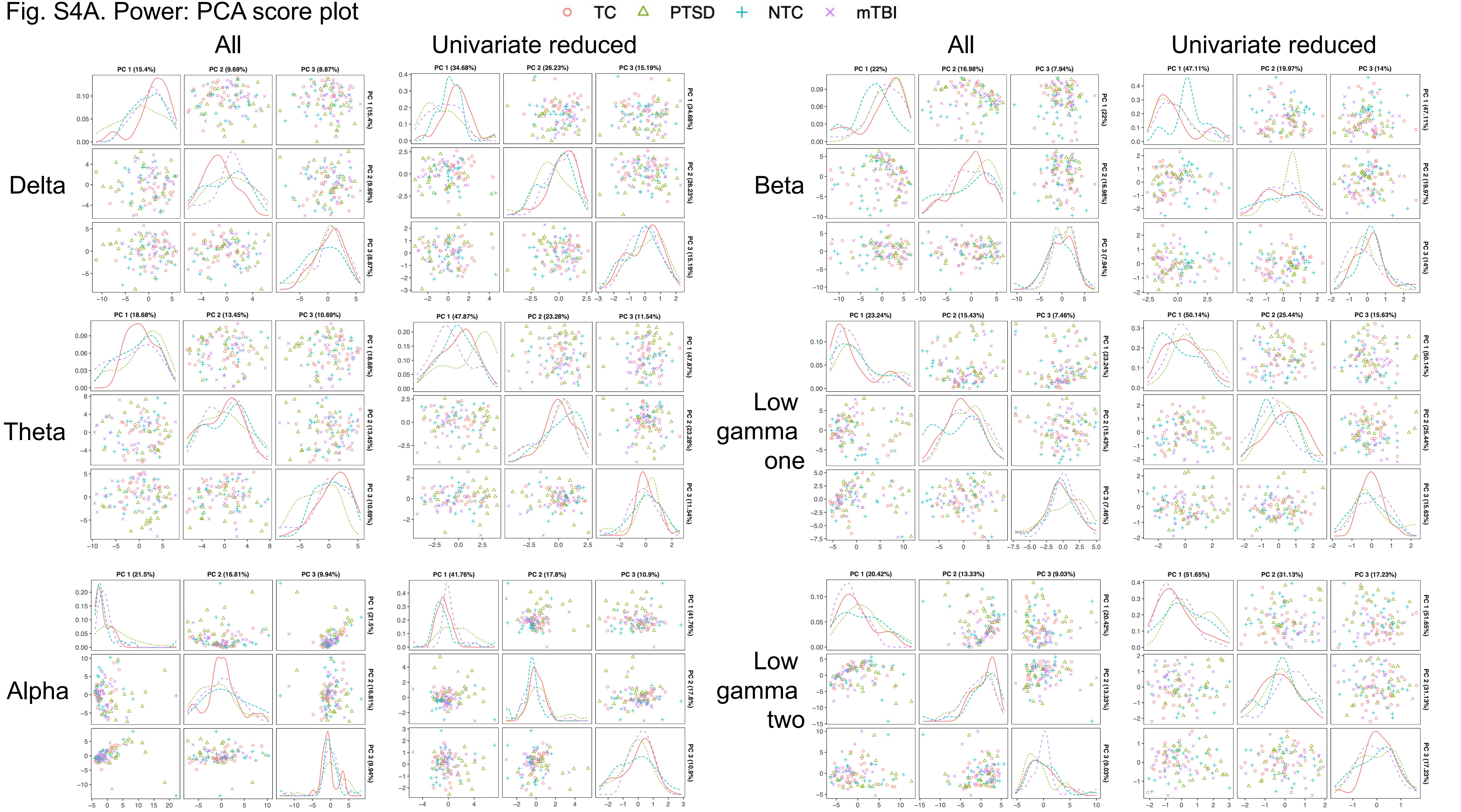

Fig. S4A. Power: PCA score plot

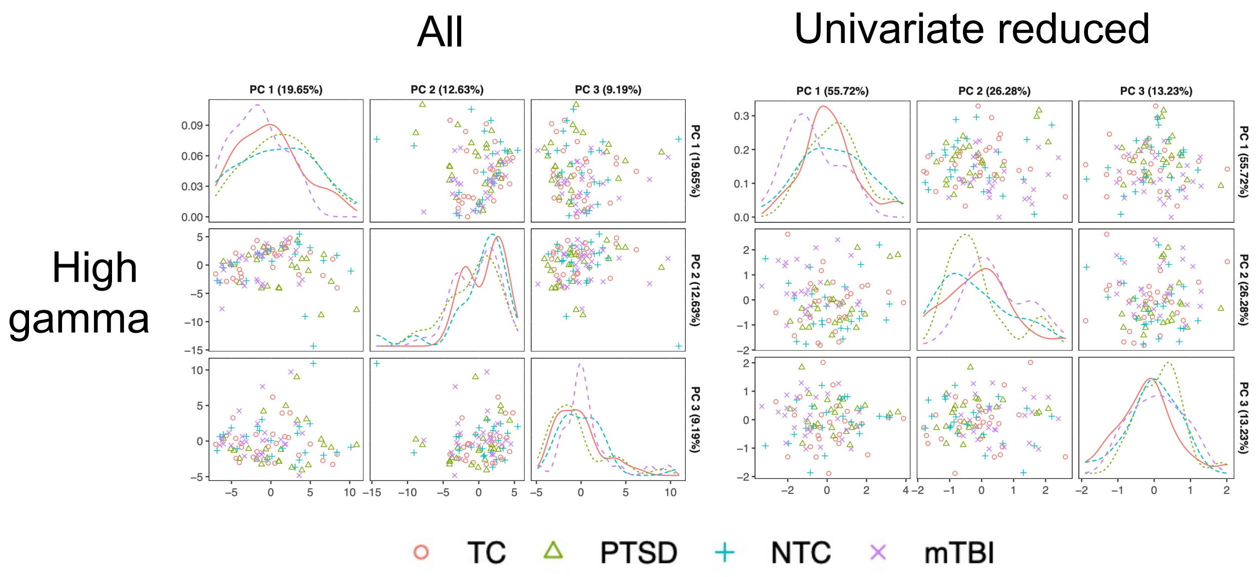

Fig S4B. AEC: PCA score plot

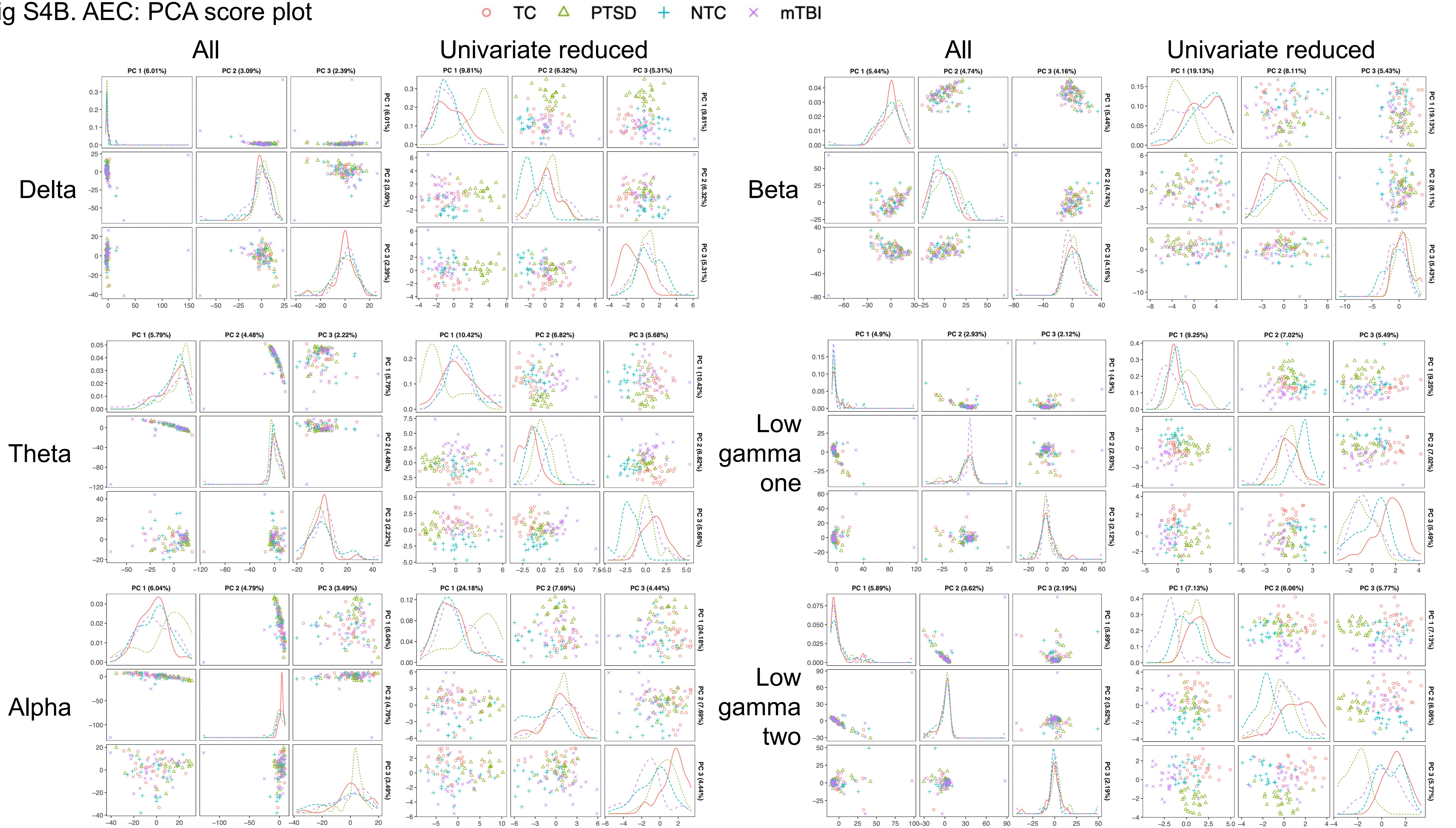

Fig. S4B. AEC: PCA score plot

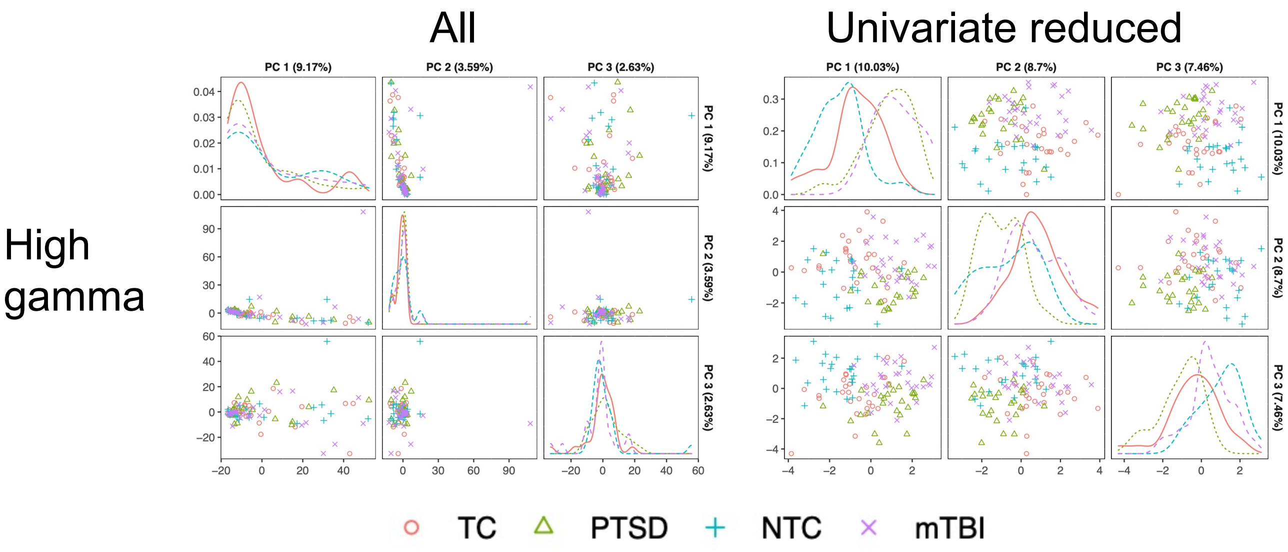

Fig. S5. Power PLS-DA permutation test

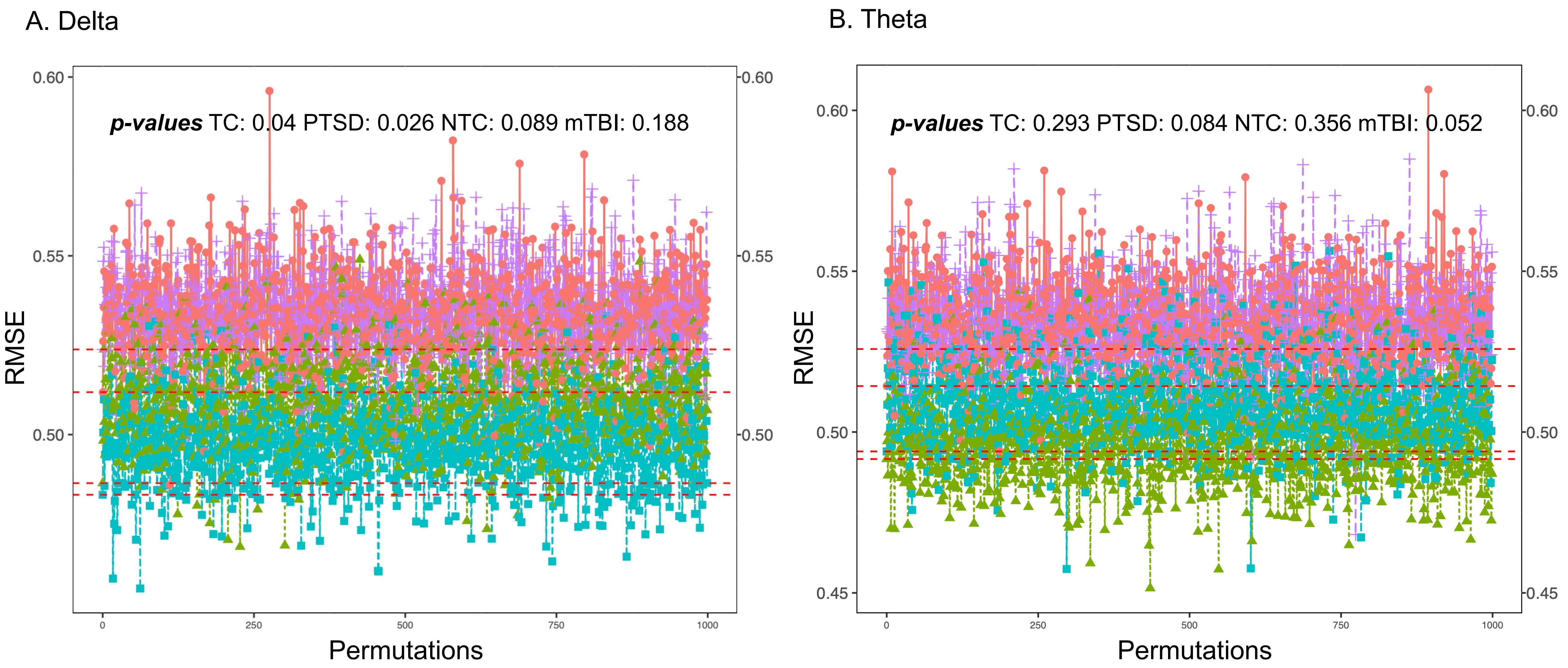

Fig. S5. Power PLS-DA permutation test

C. Alpha

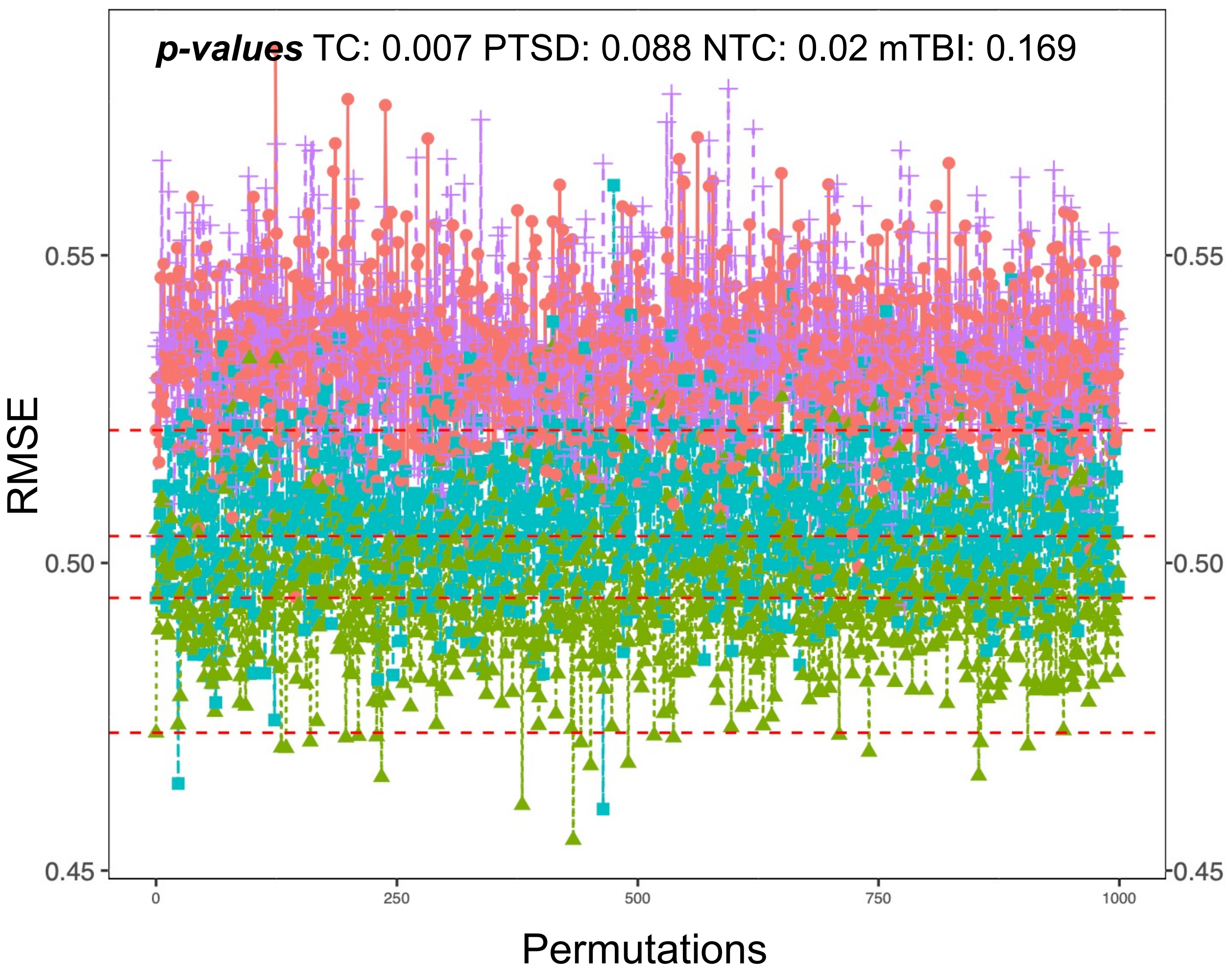

D. Beta

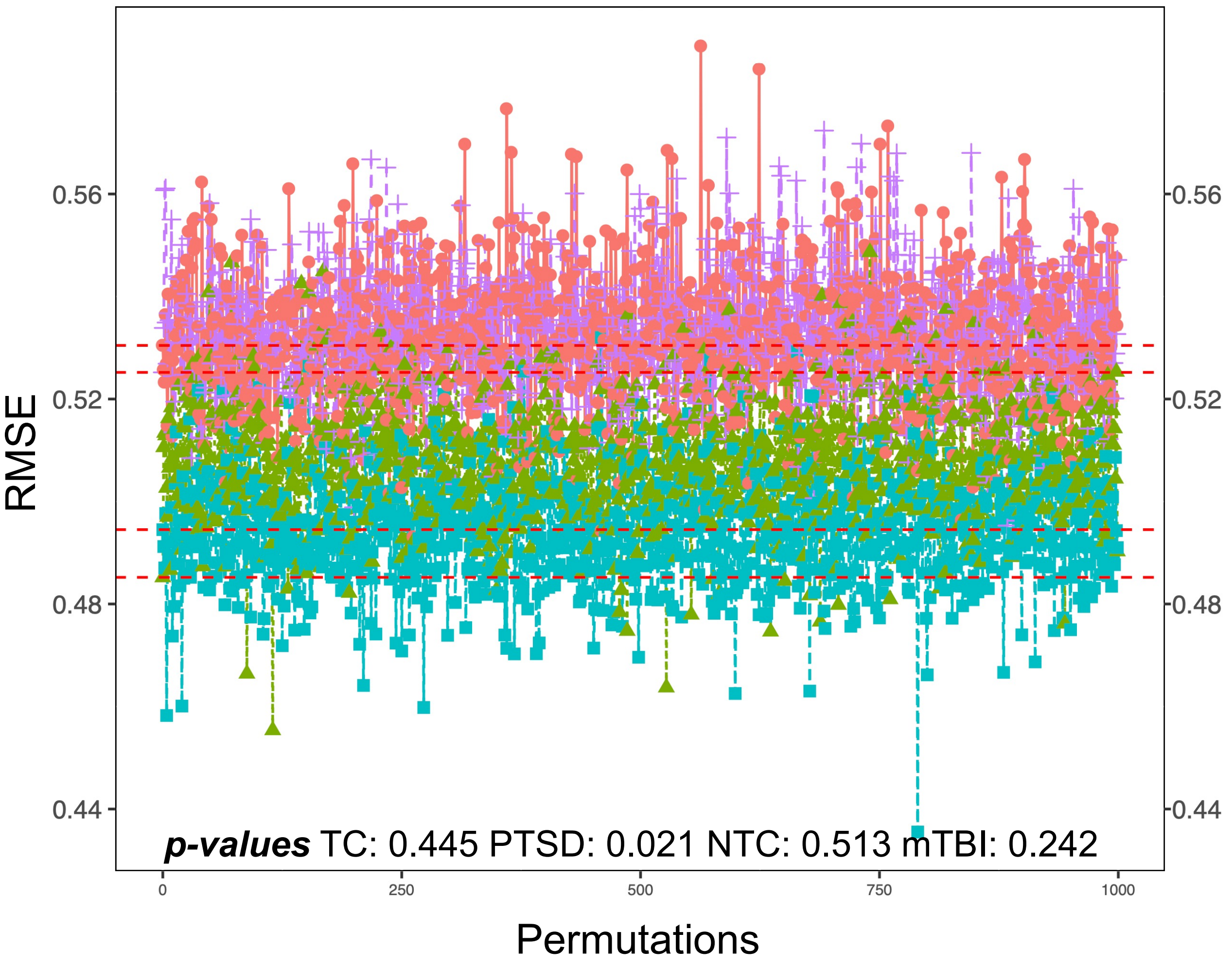

Fig. S5. Power PLS-DA permutation test

E. Low gamma one

F. Low gamma two

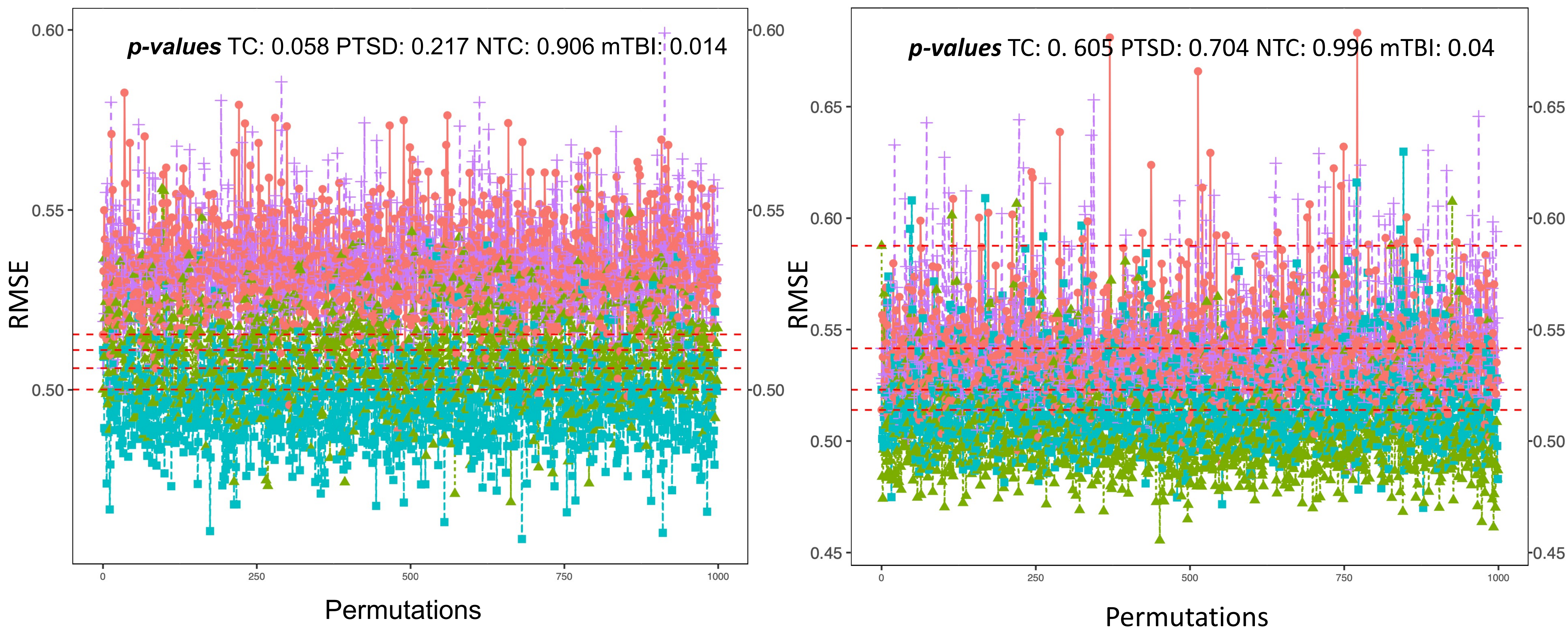

Fig. S5. Power PLS-DA permutation test

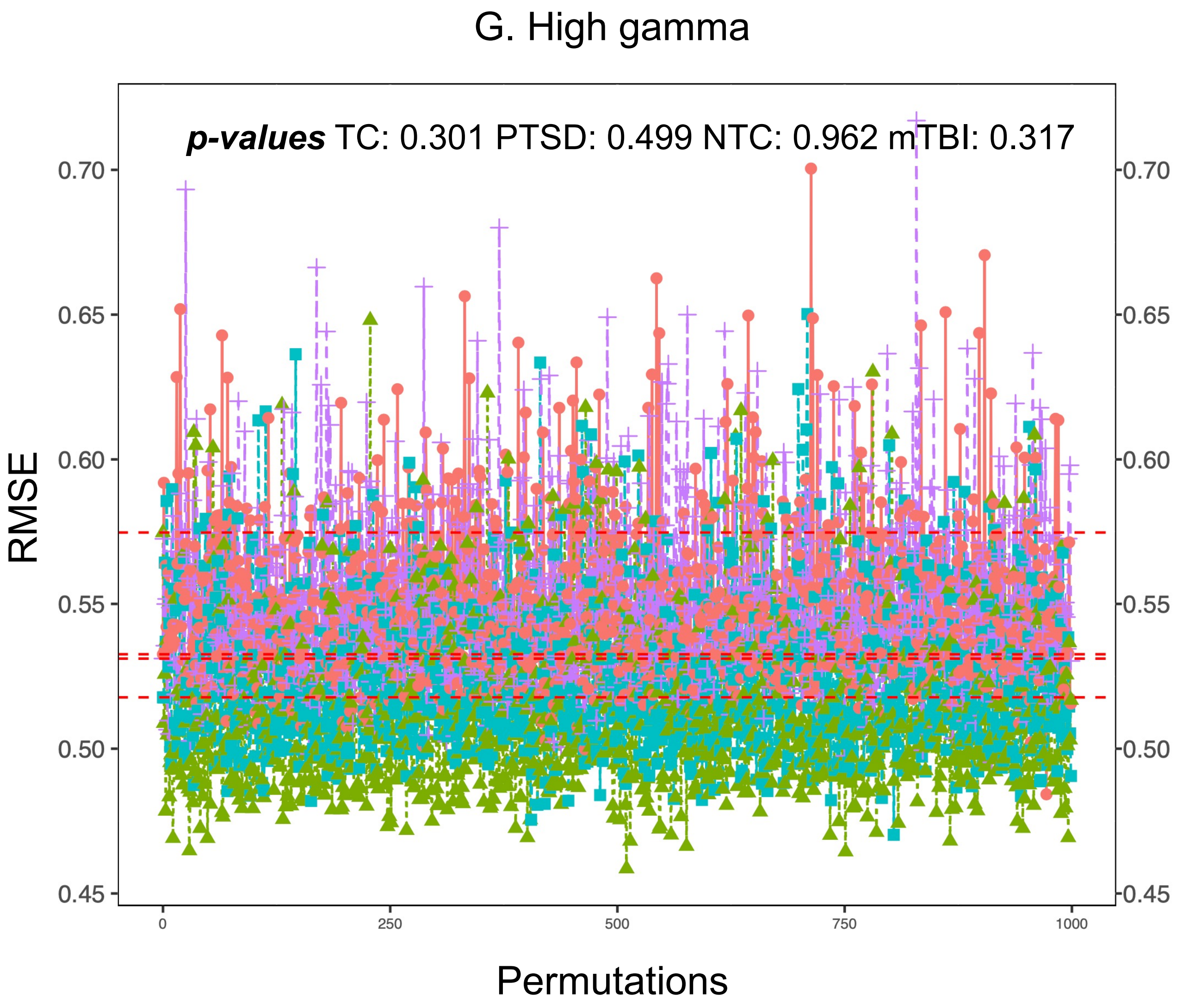

Fig. S6. AEC PLS-DA permutation test

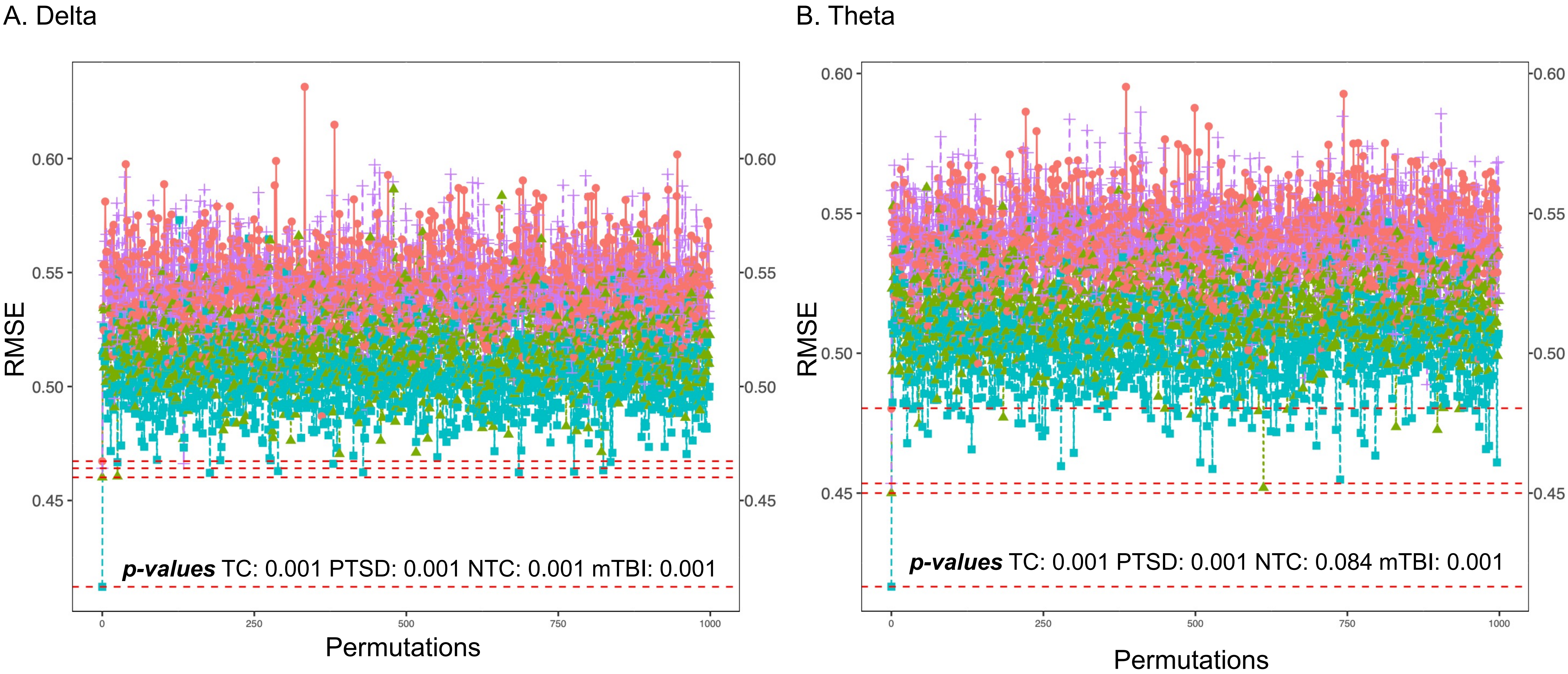

Fig. S6. AEC PLS-DA permutation test

C. Alpha

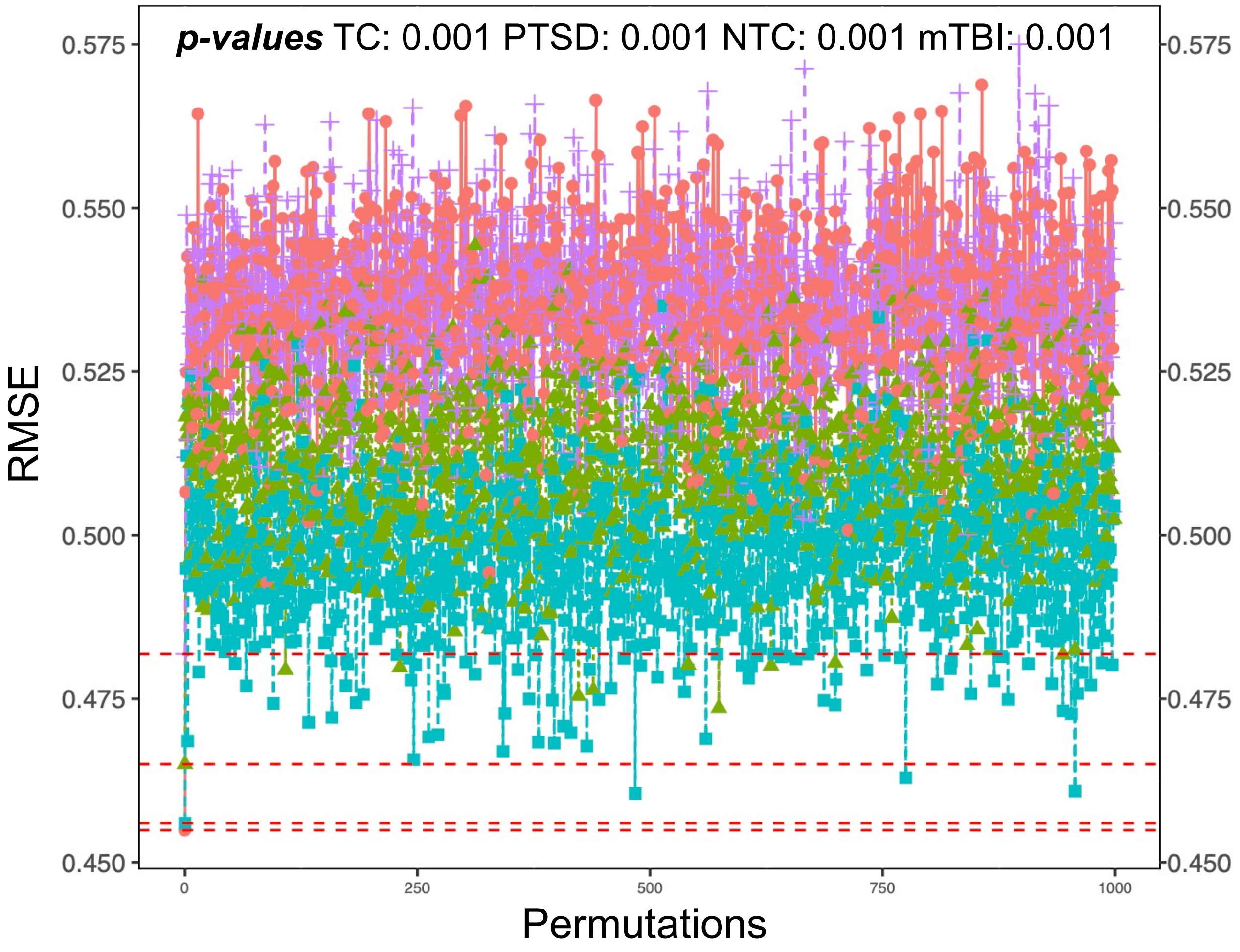

D. Beta

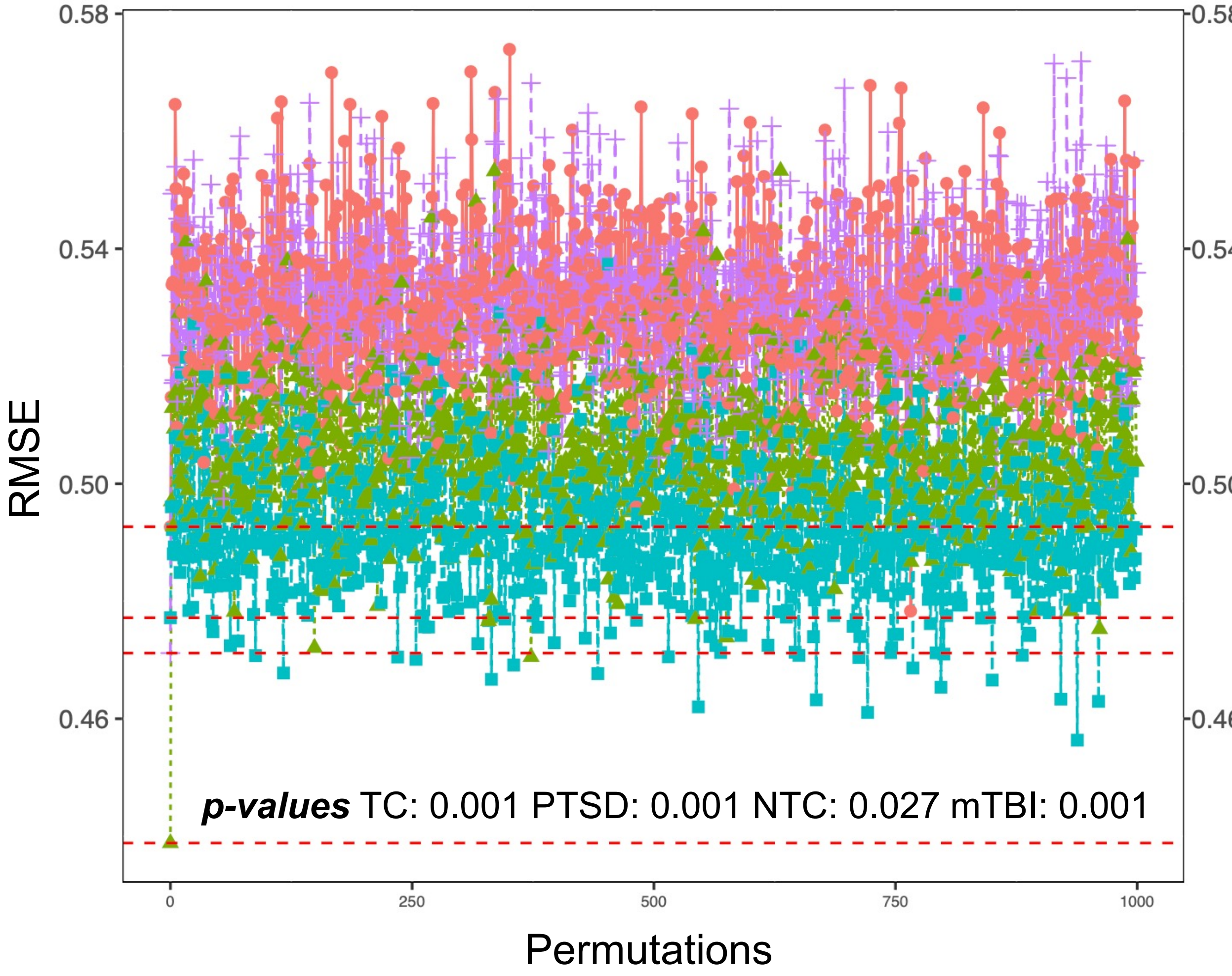

Fig. S6. AEC PLS-DA permutation test

E. Low gamma one

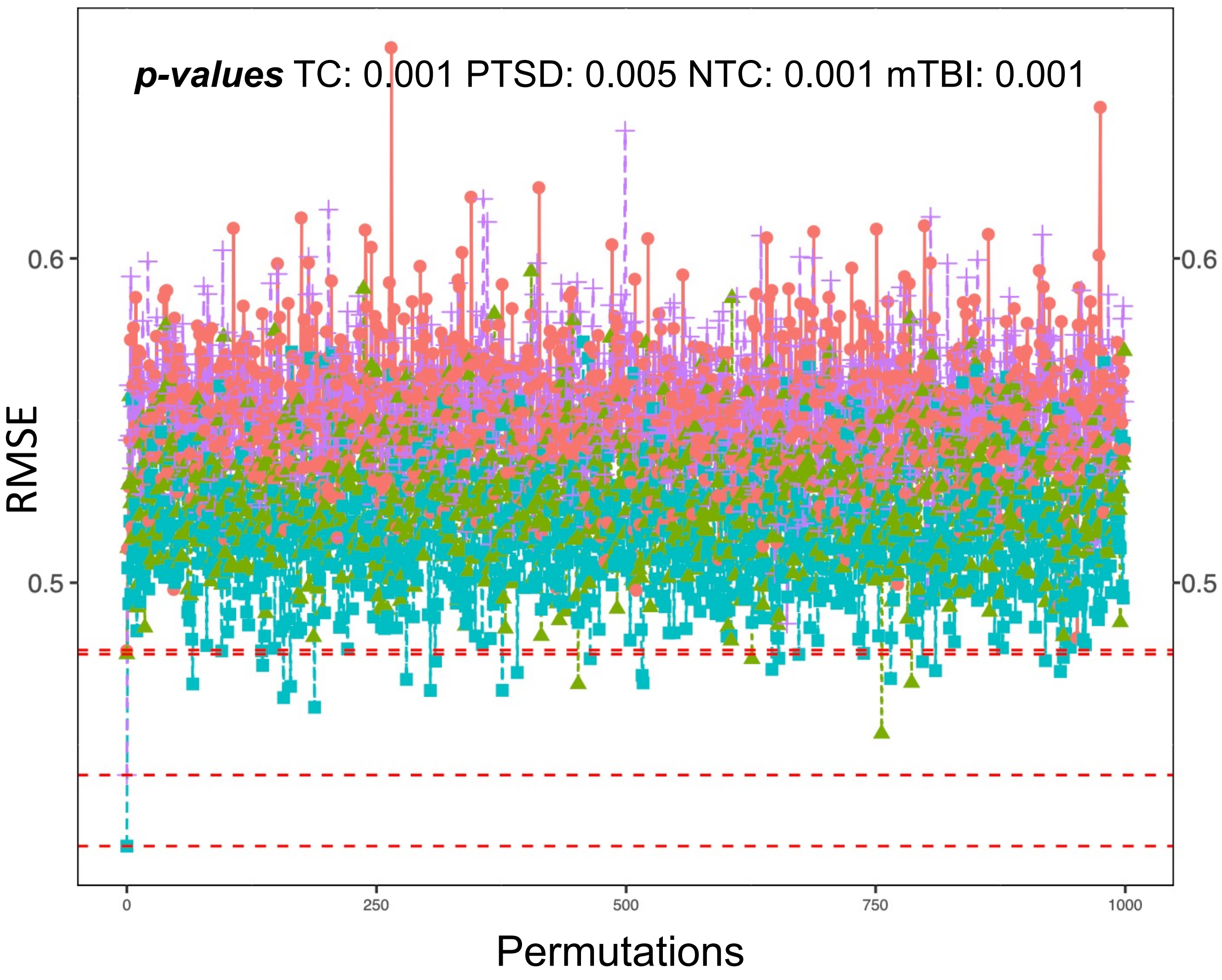

F. Low gamma two

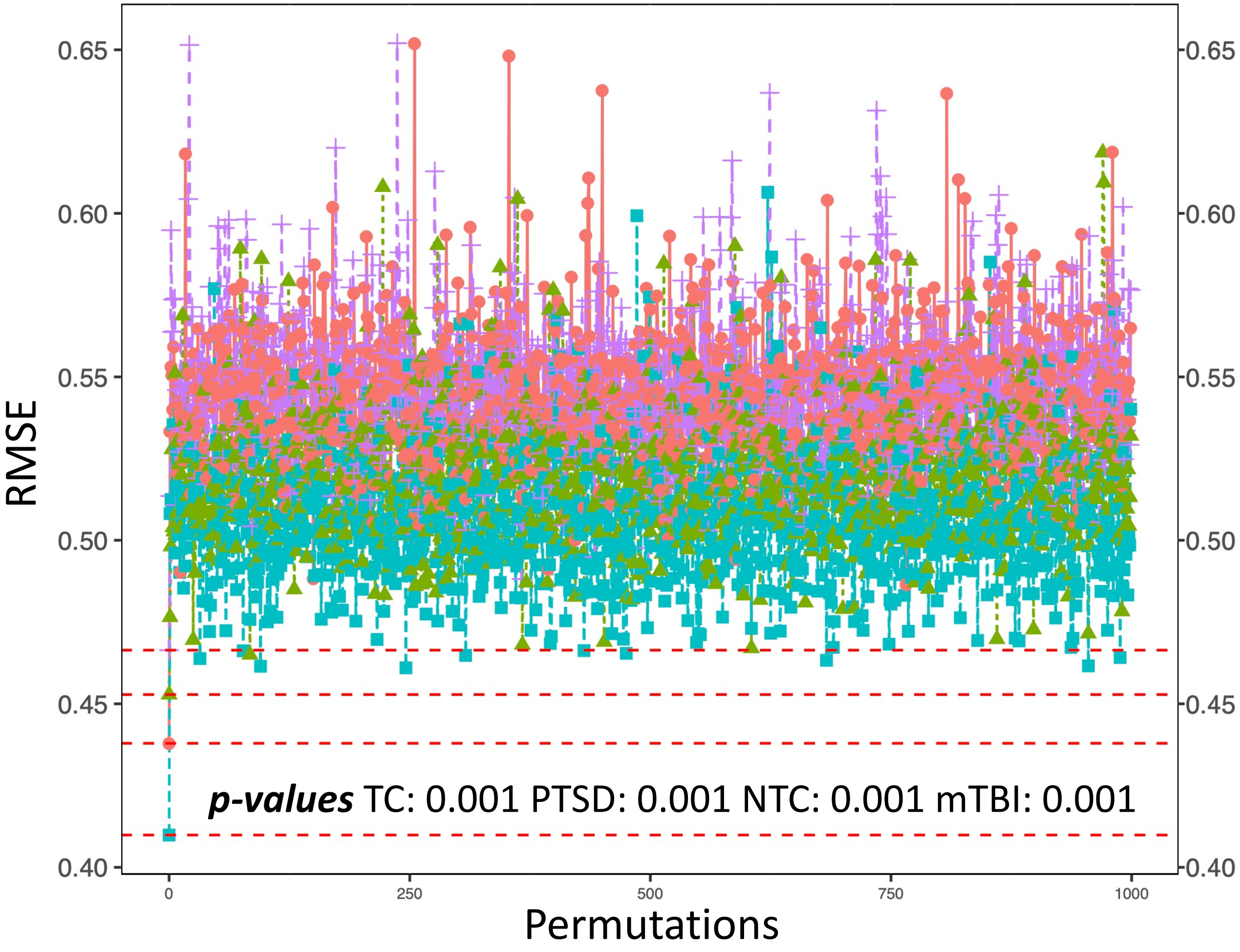

Fig. S6. AEC PLS-DA permutation test

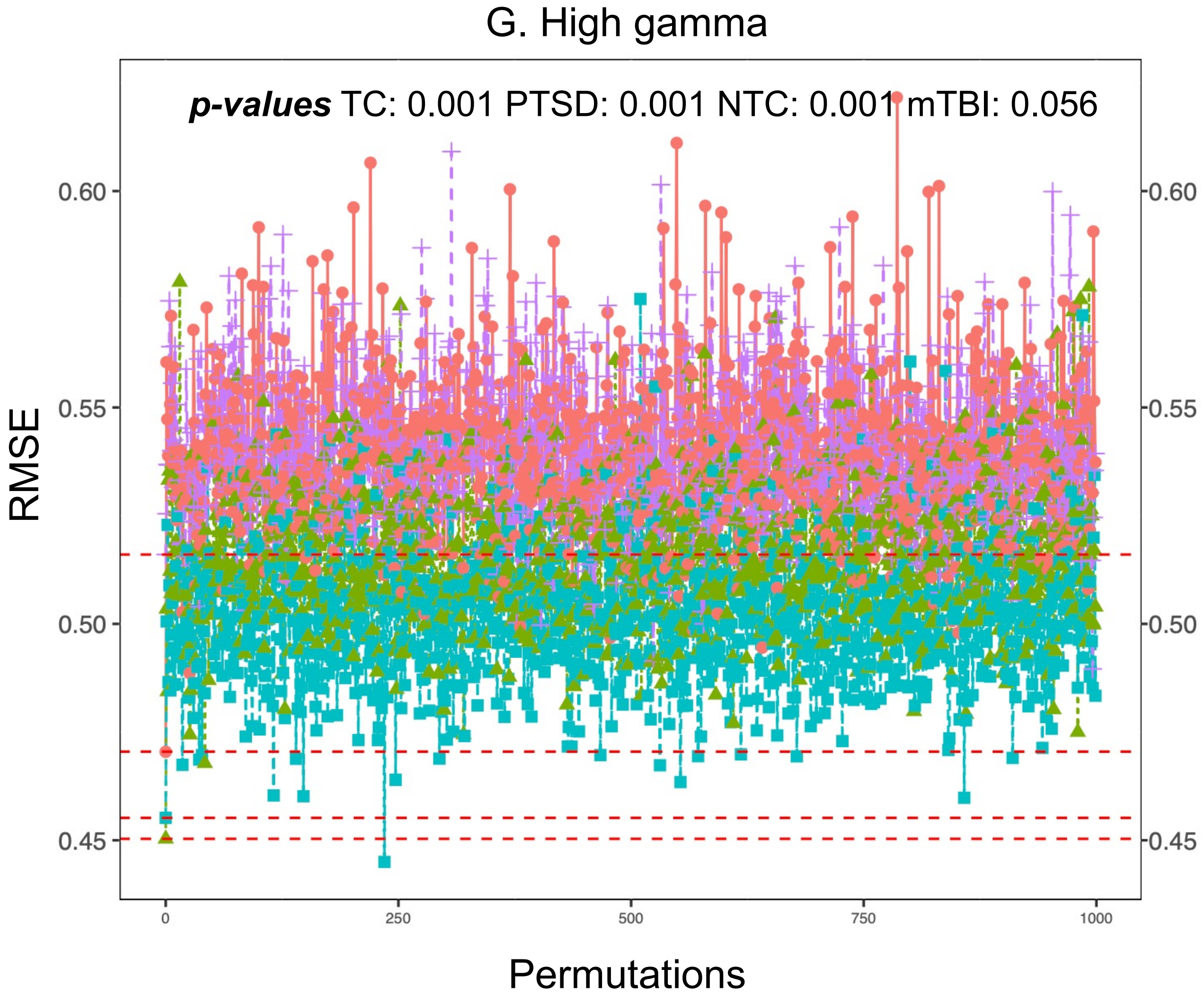

Fig. S7A – Power: cross validation AUC

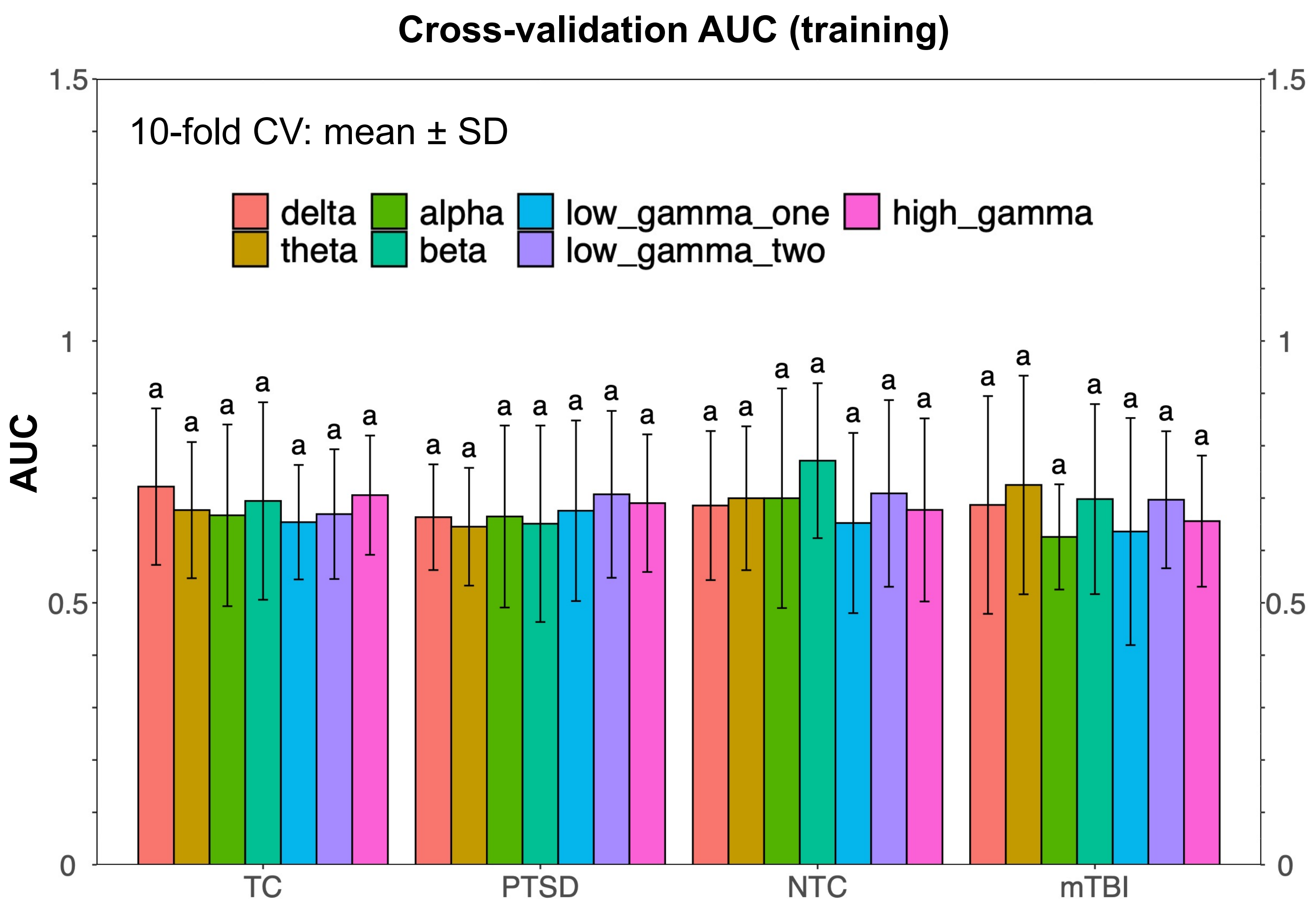

Fig. S7B – AEC: cross validation AUC

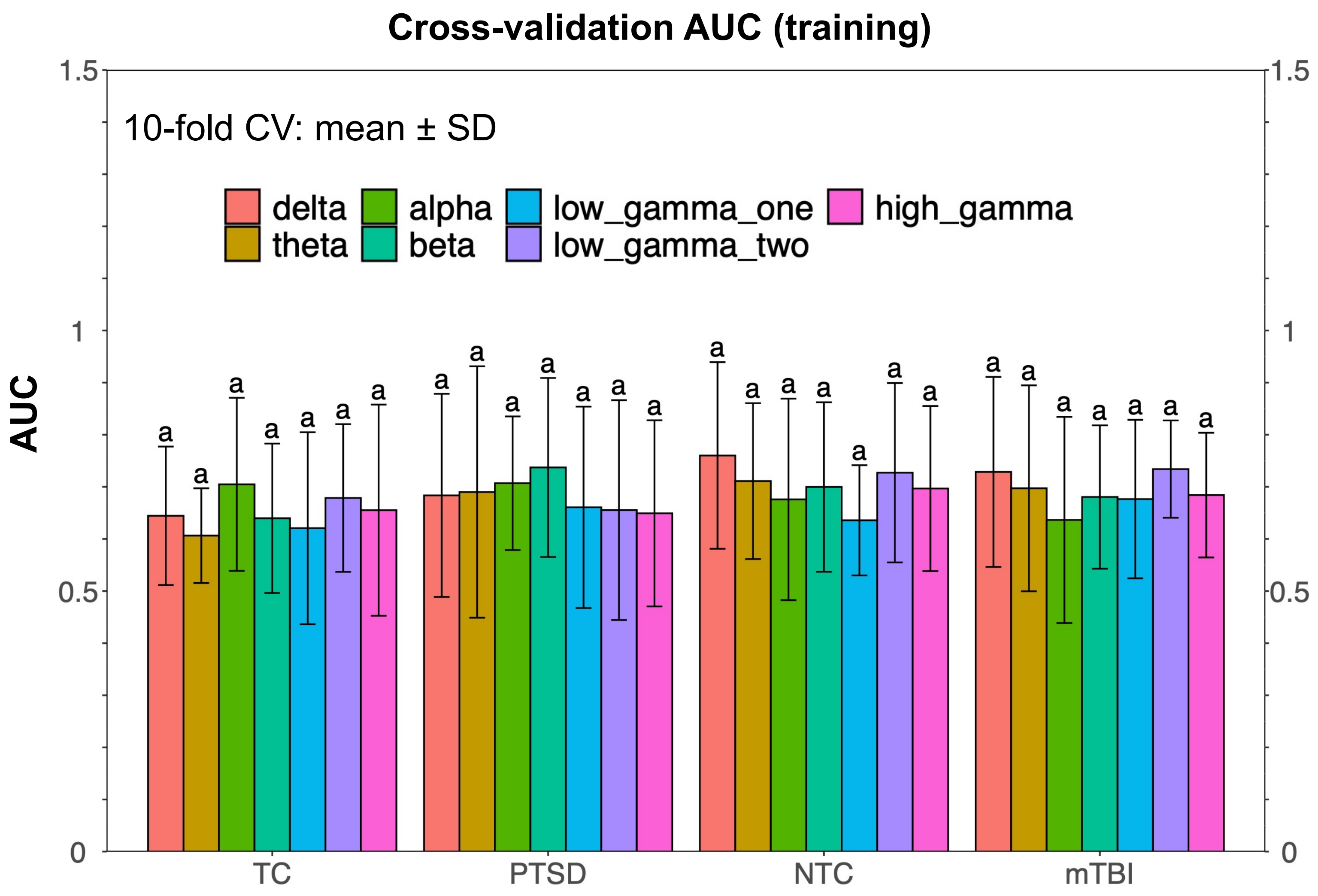

Fig. S8 – power: SVM permutation

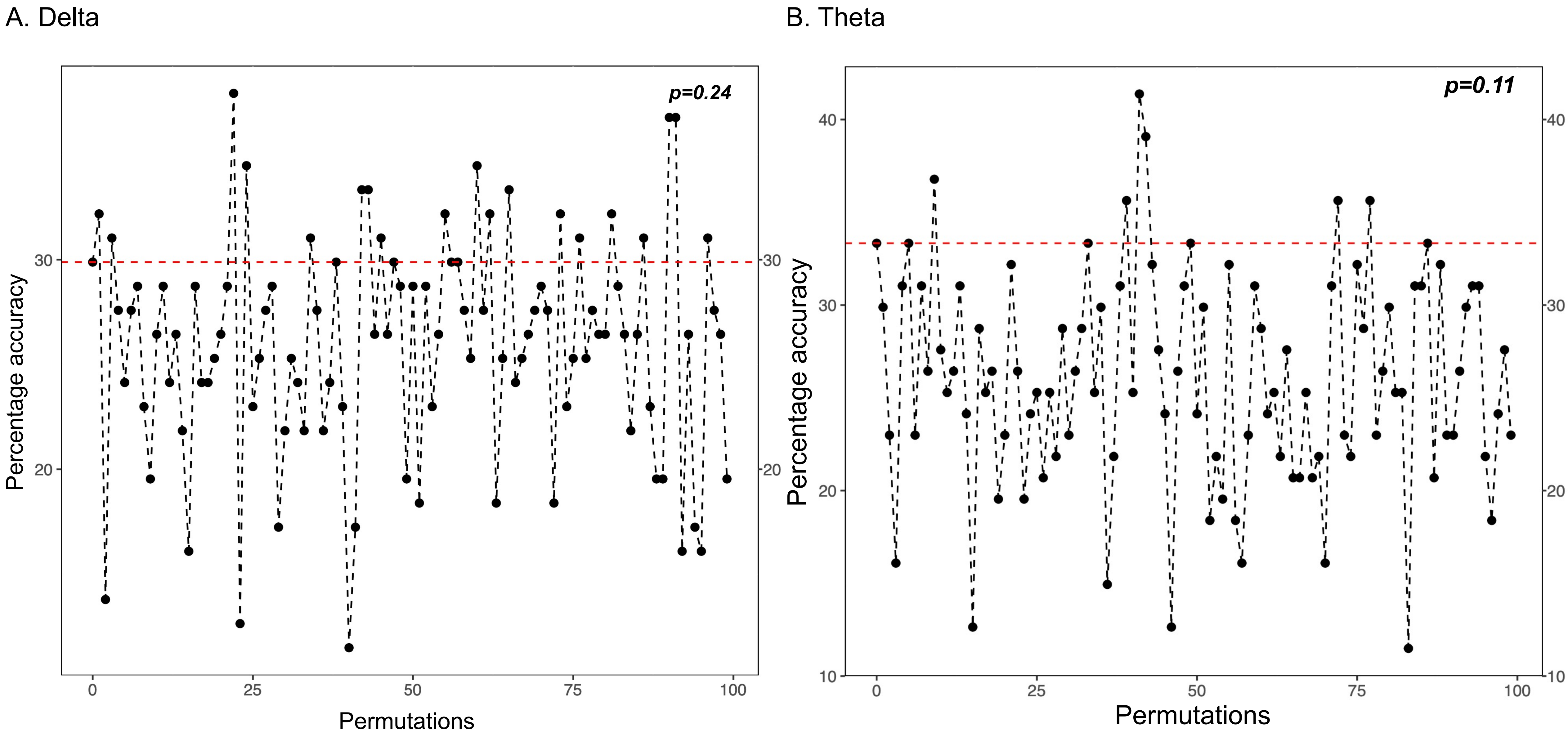

Fig. S8 – power: SVM permutation

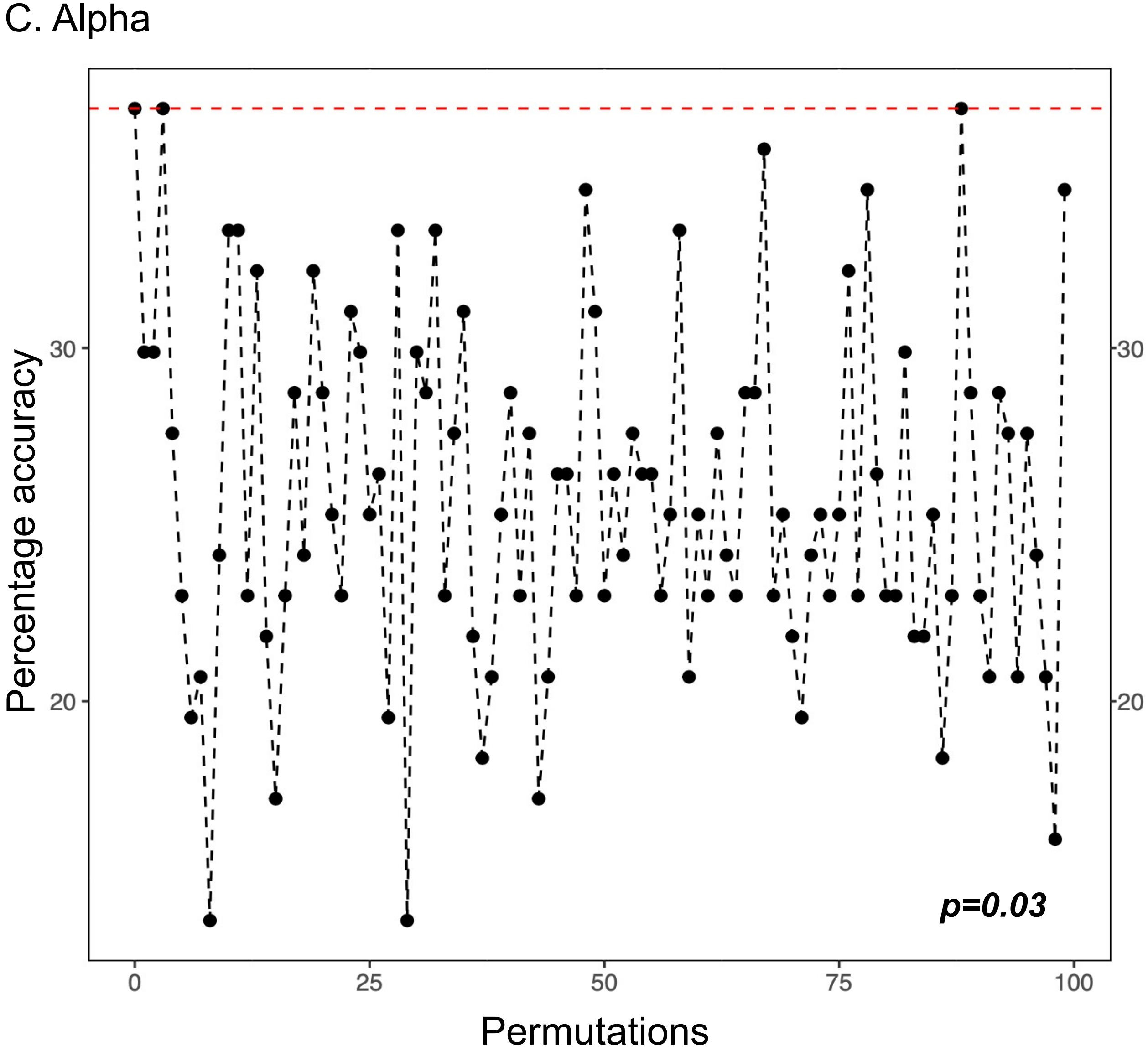

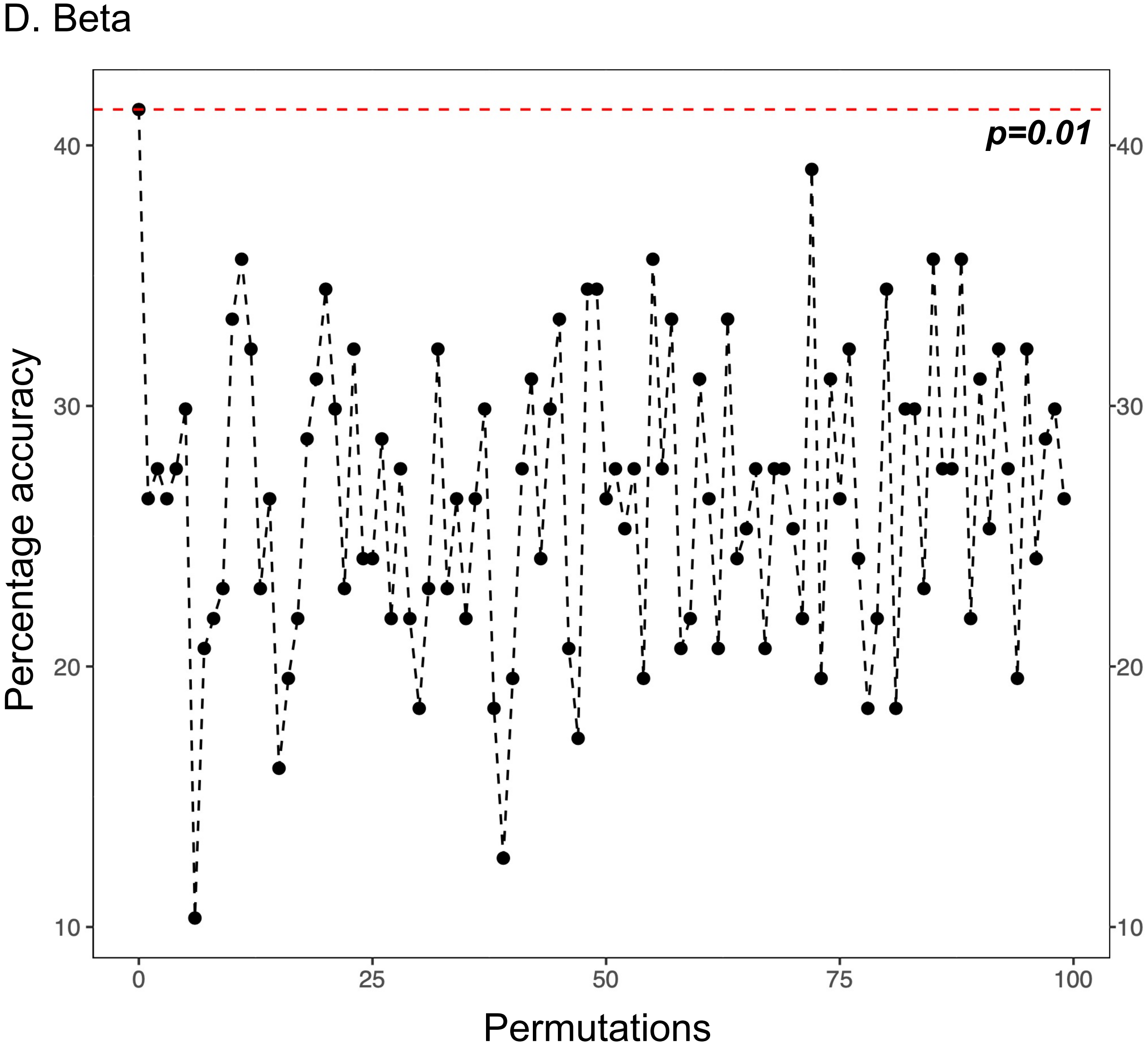

Fig. S8 – power: SVM permutation

E. Low gamma one

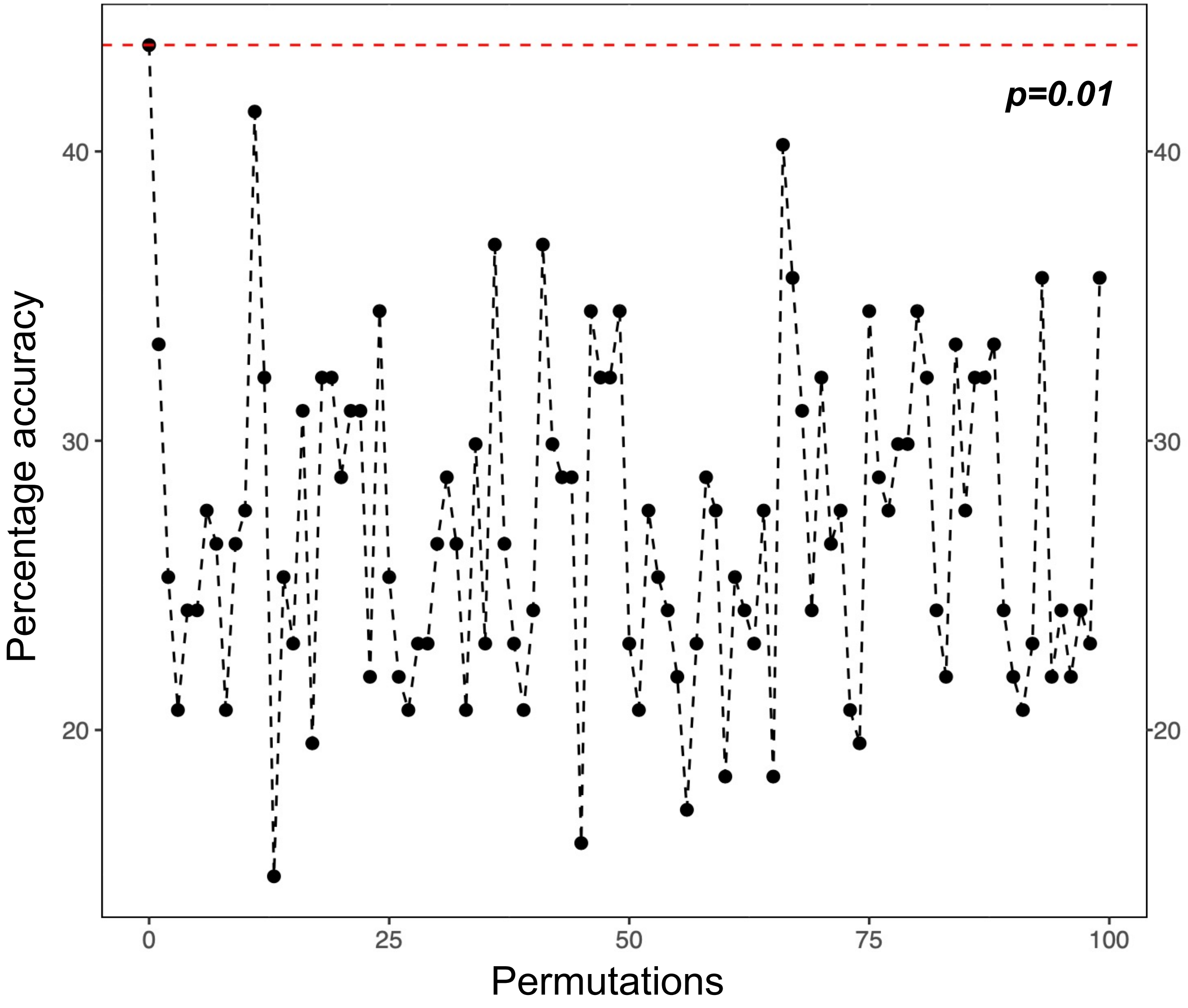

F. Low gamma two

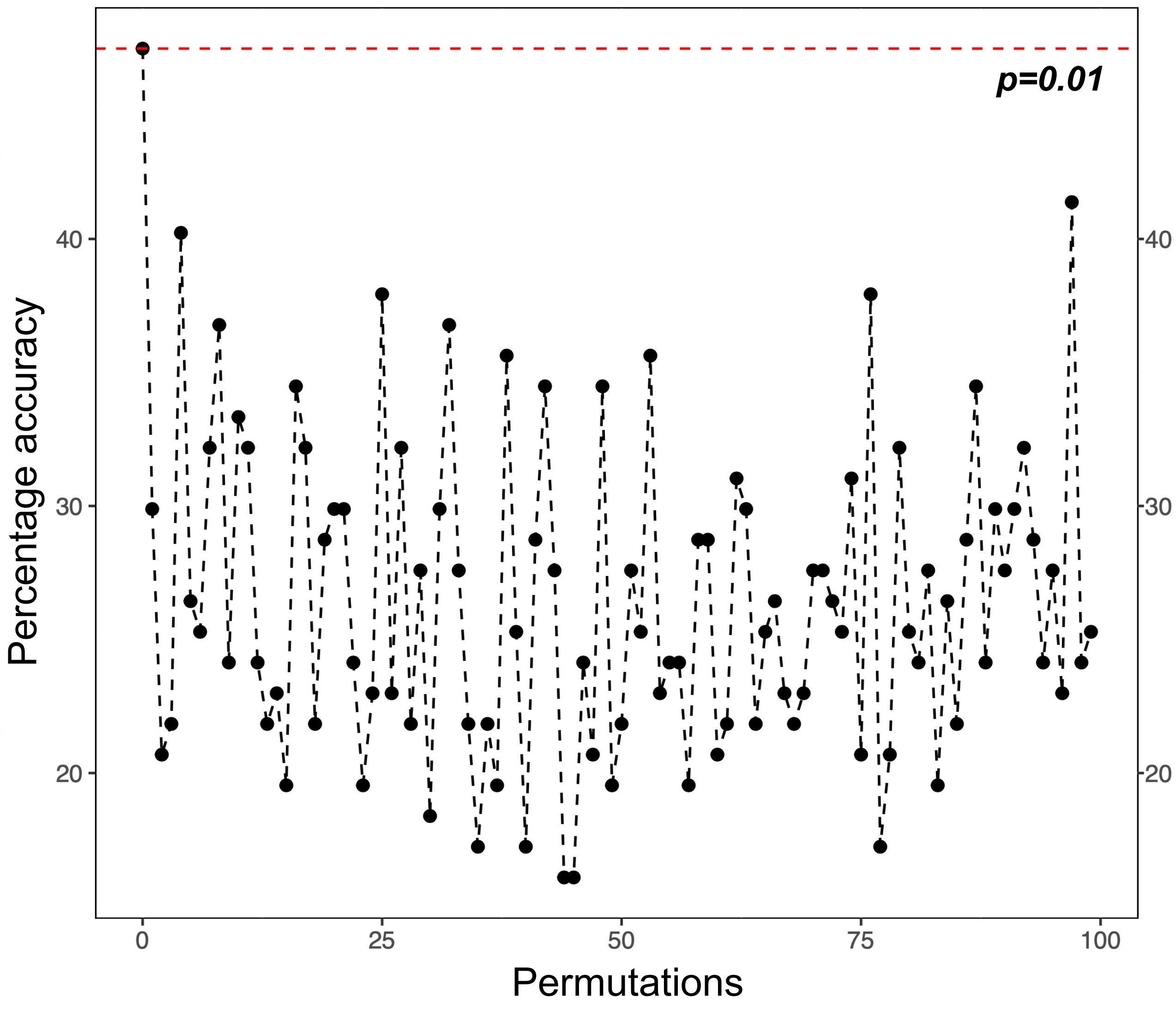

Fig. S8 – power: SVM permutation

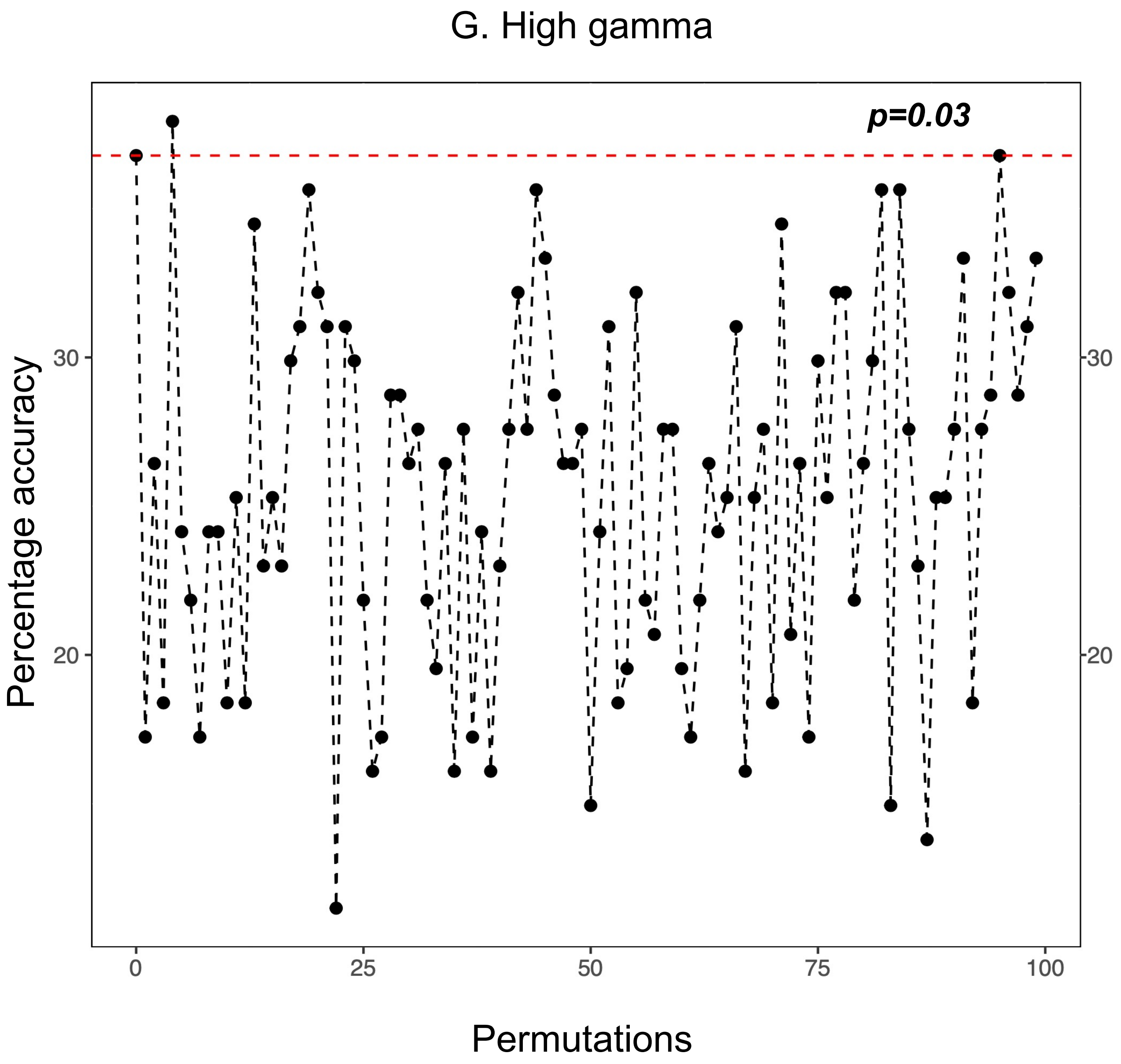

Fig. S9 – AEC: SVM permutation

A. Delta

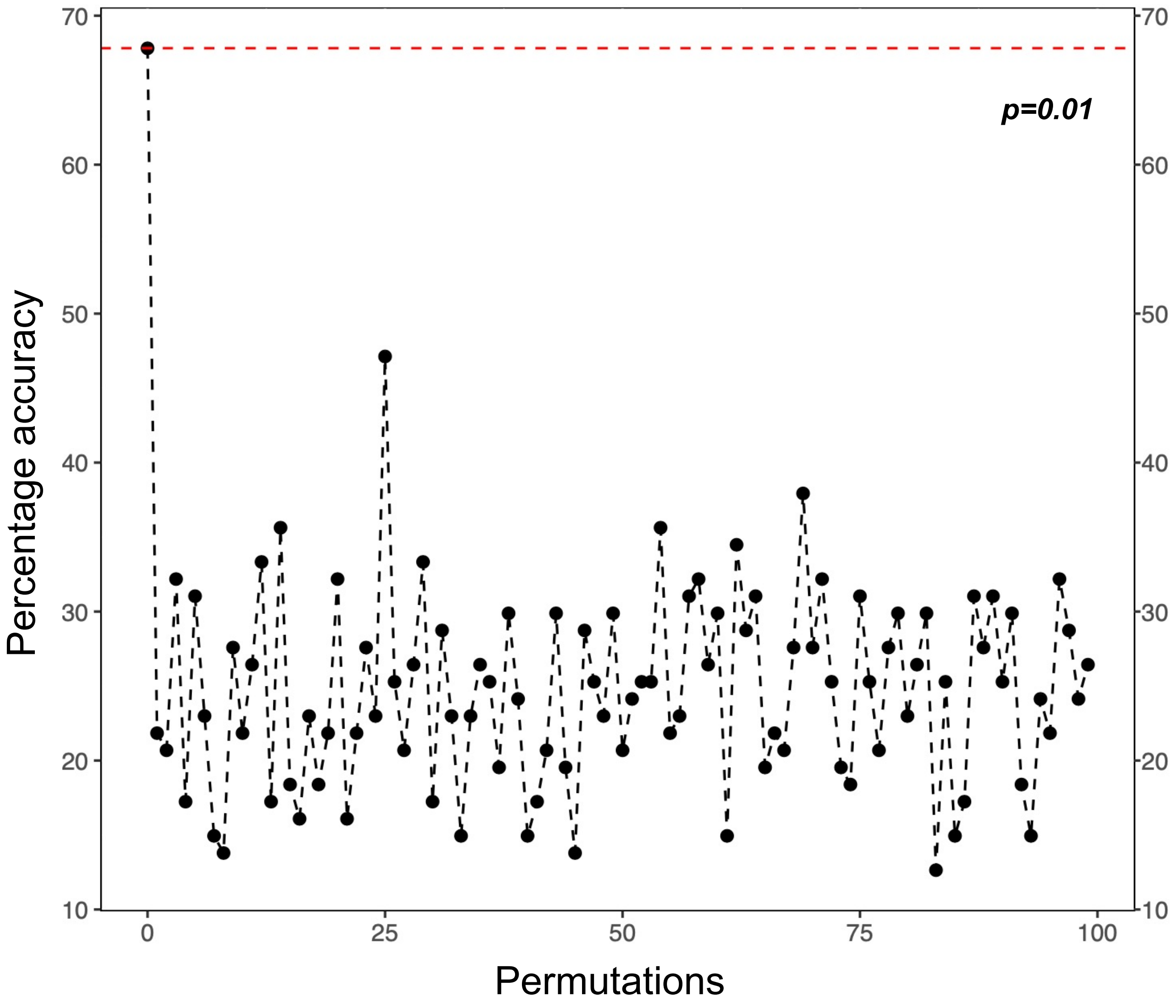

B. Theta

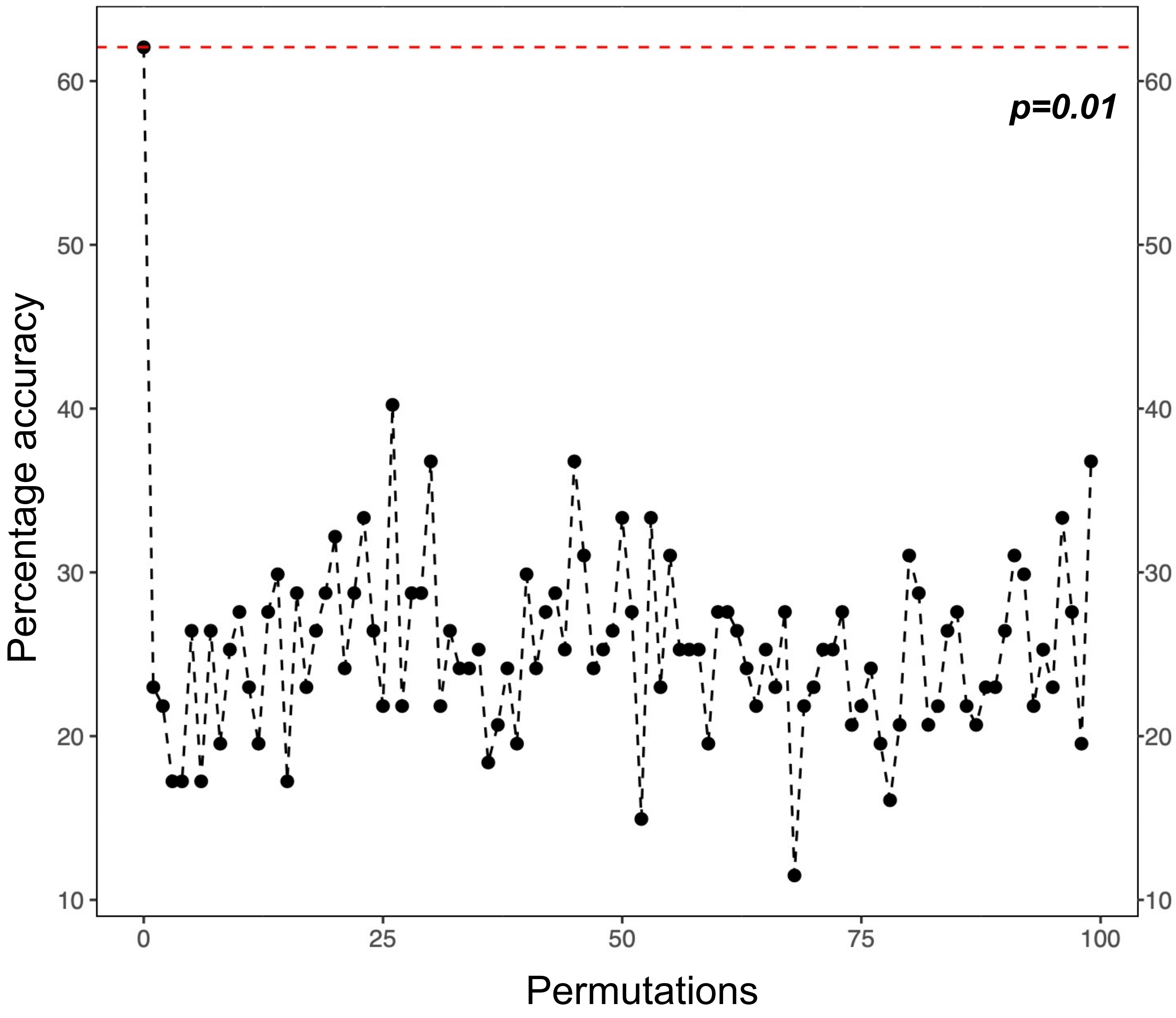

Fig. S9 – AEC: SVM permutation

C. Alpha

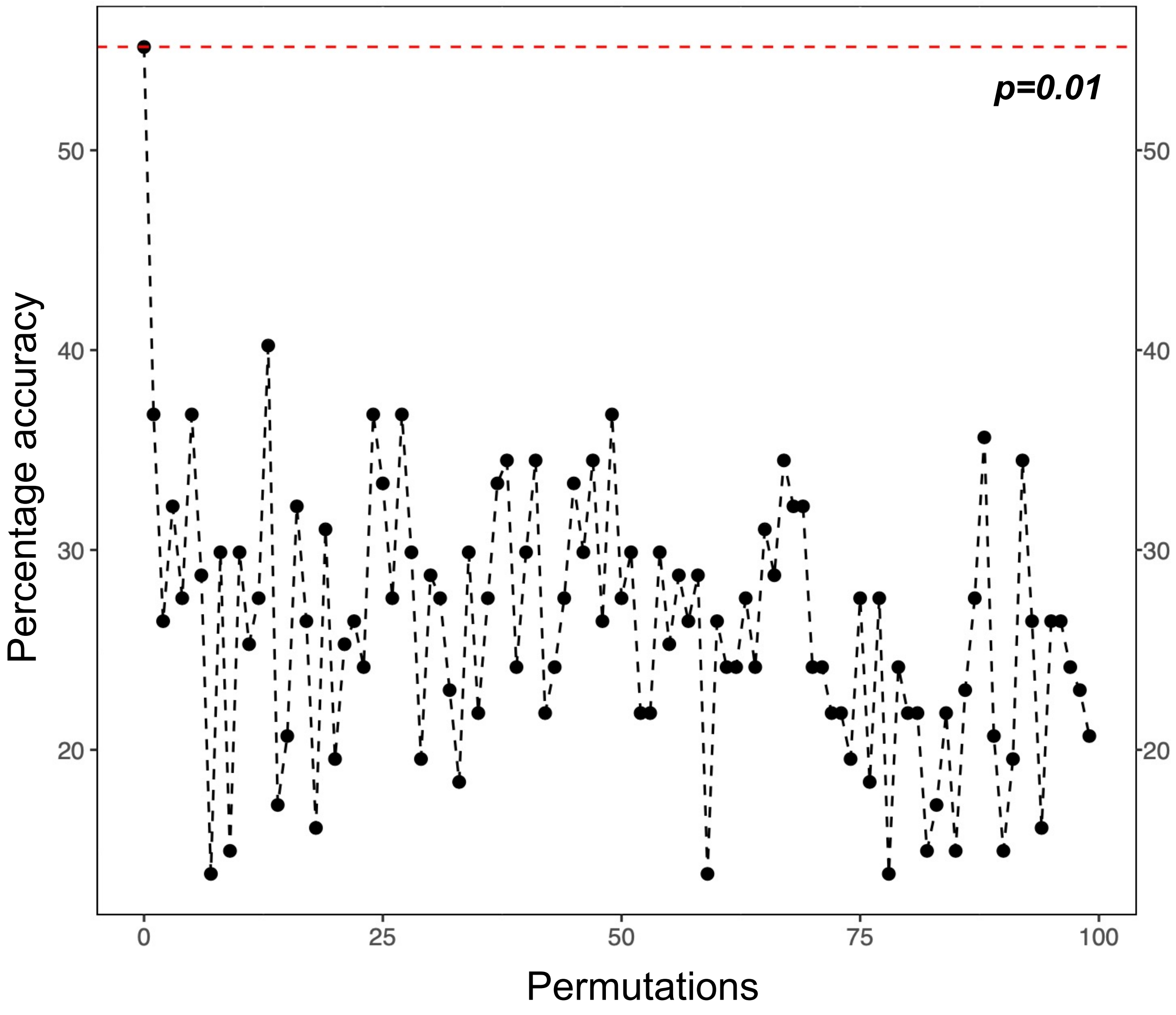

D. Beta

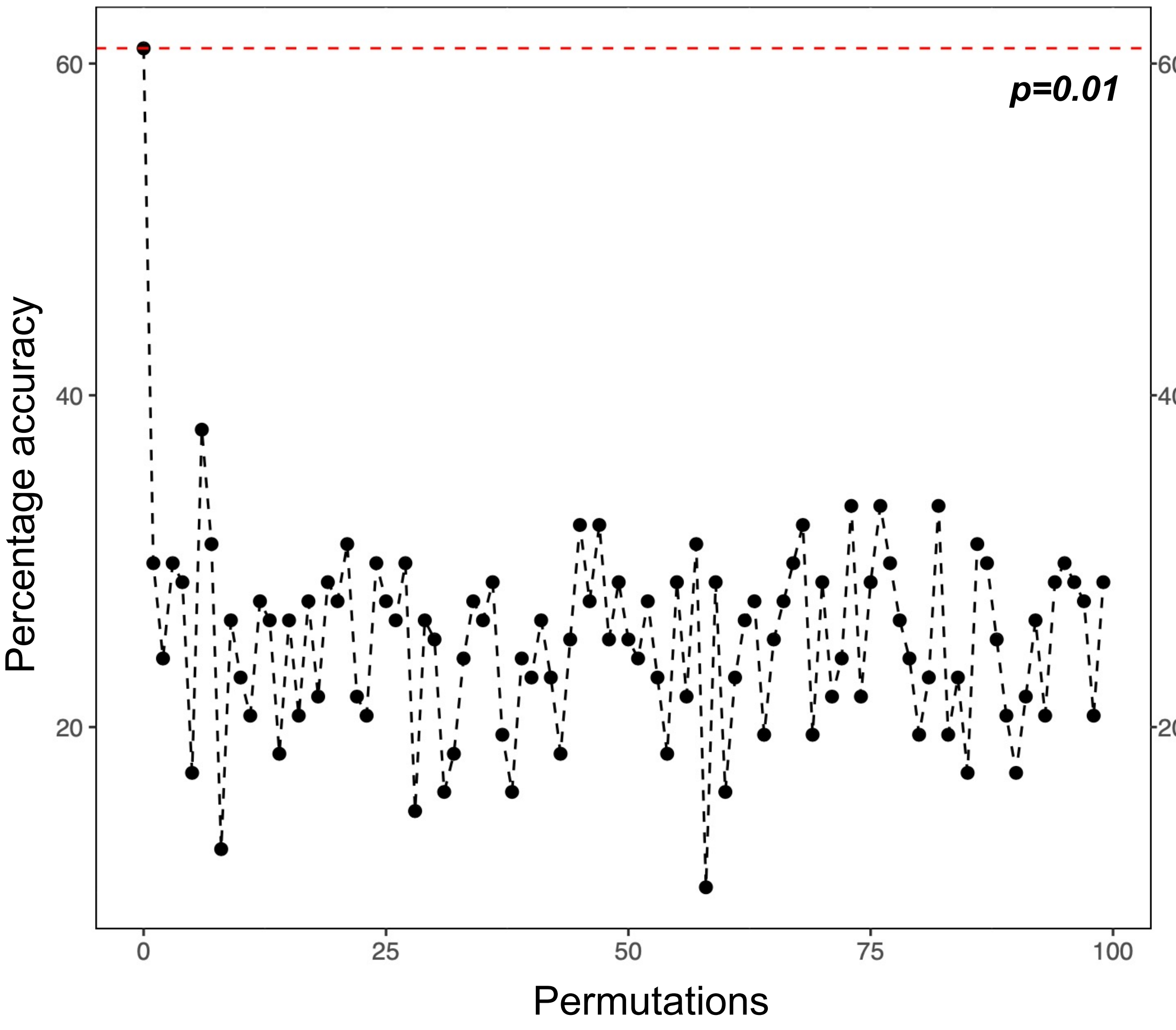

Fig. S9 – AEC: SVM permutation

E. Low gamma one

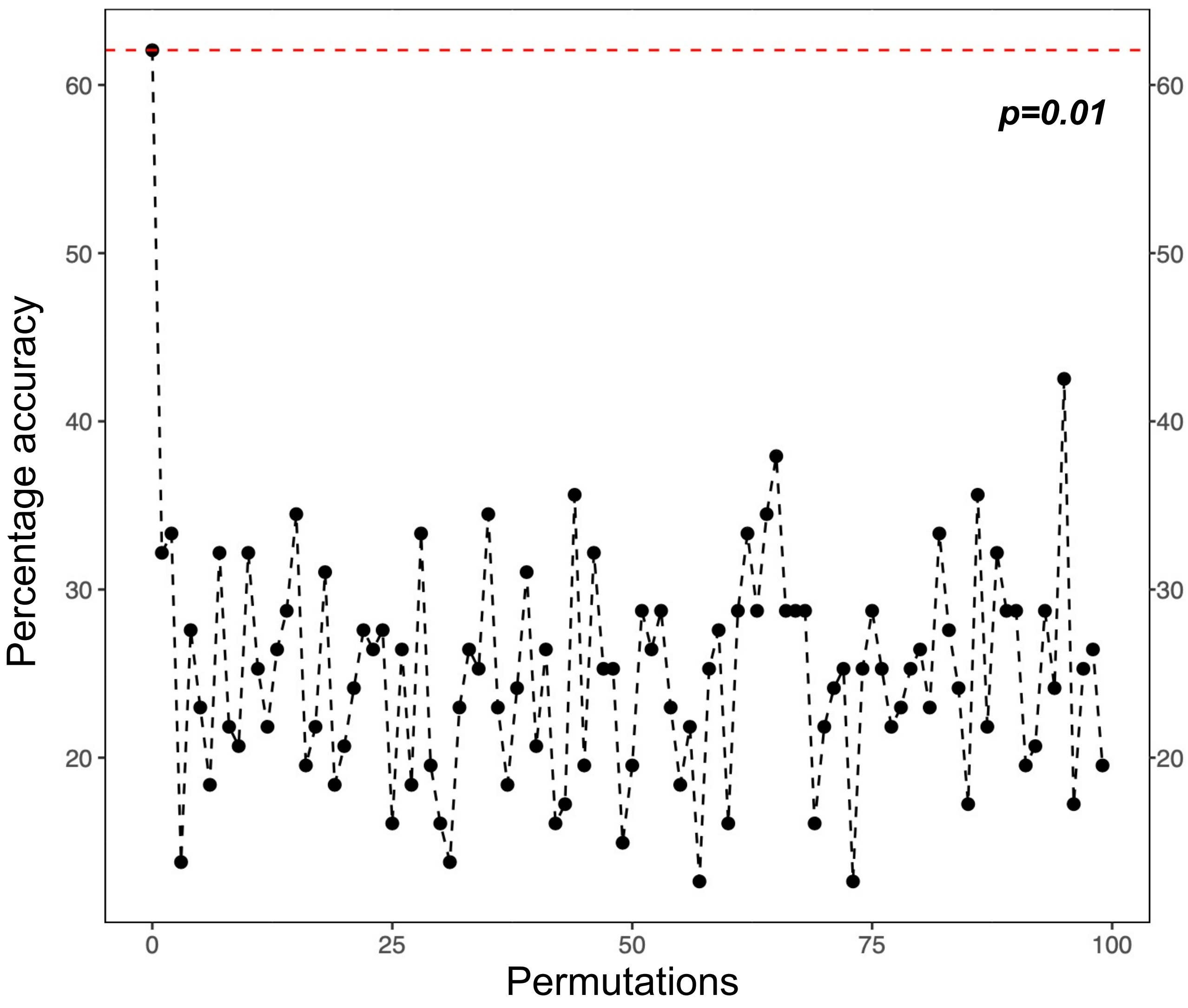

F. Low gamma two

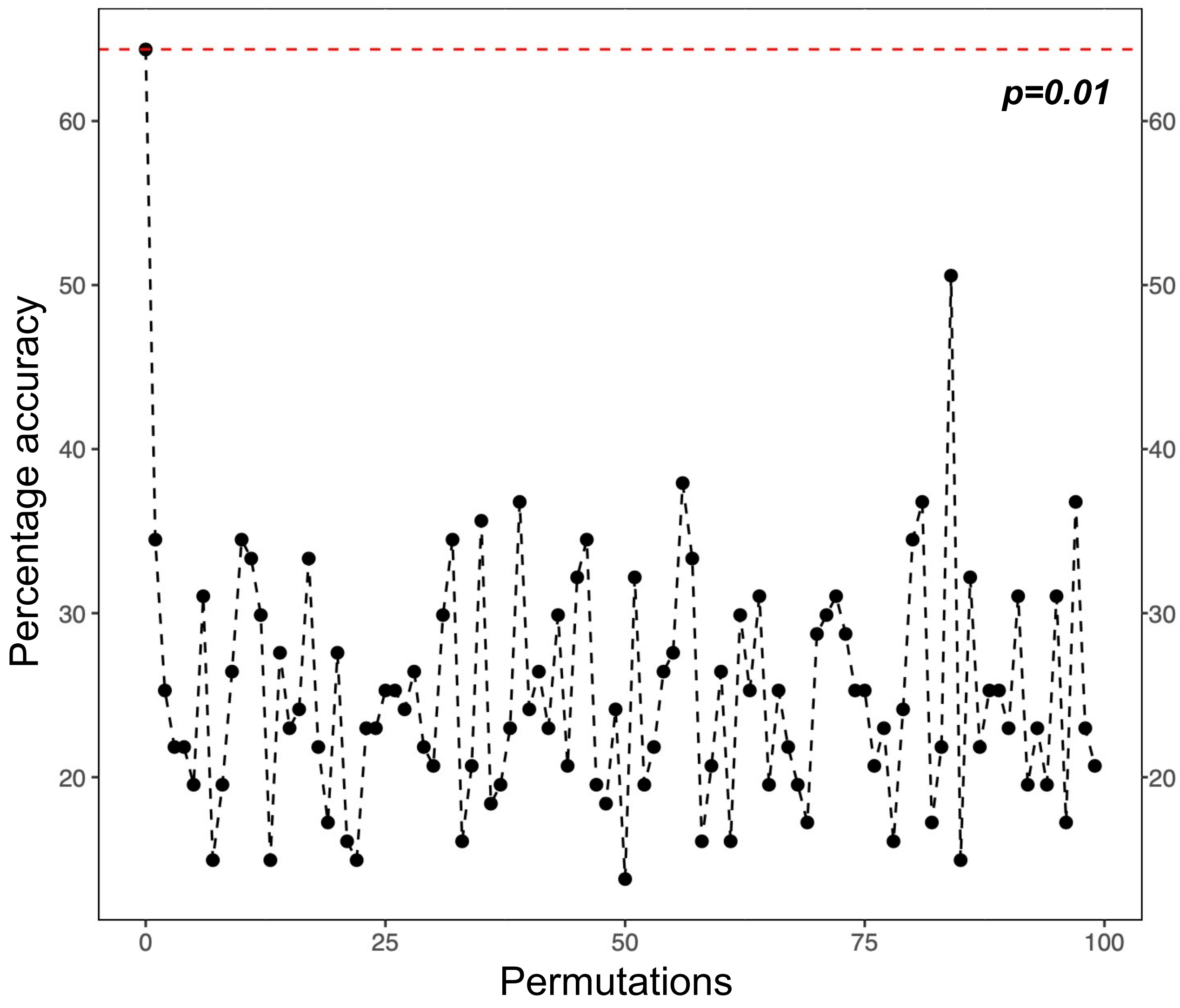

Fig. S9 – AEC: SVM permutation

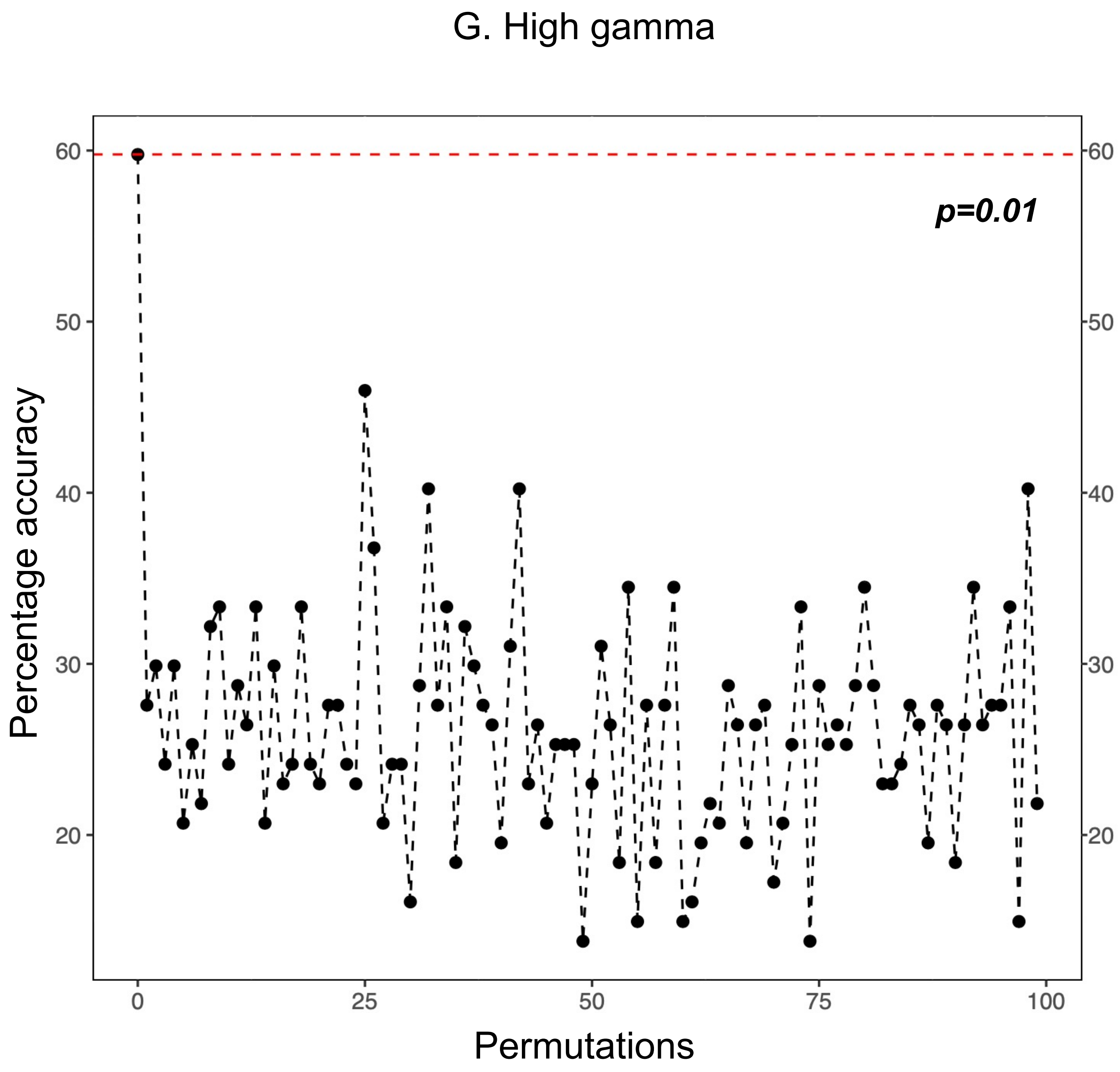

Fig. S10 – power: final ROC-AUC

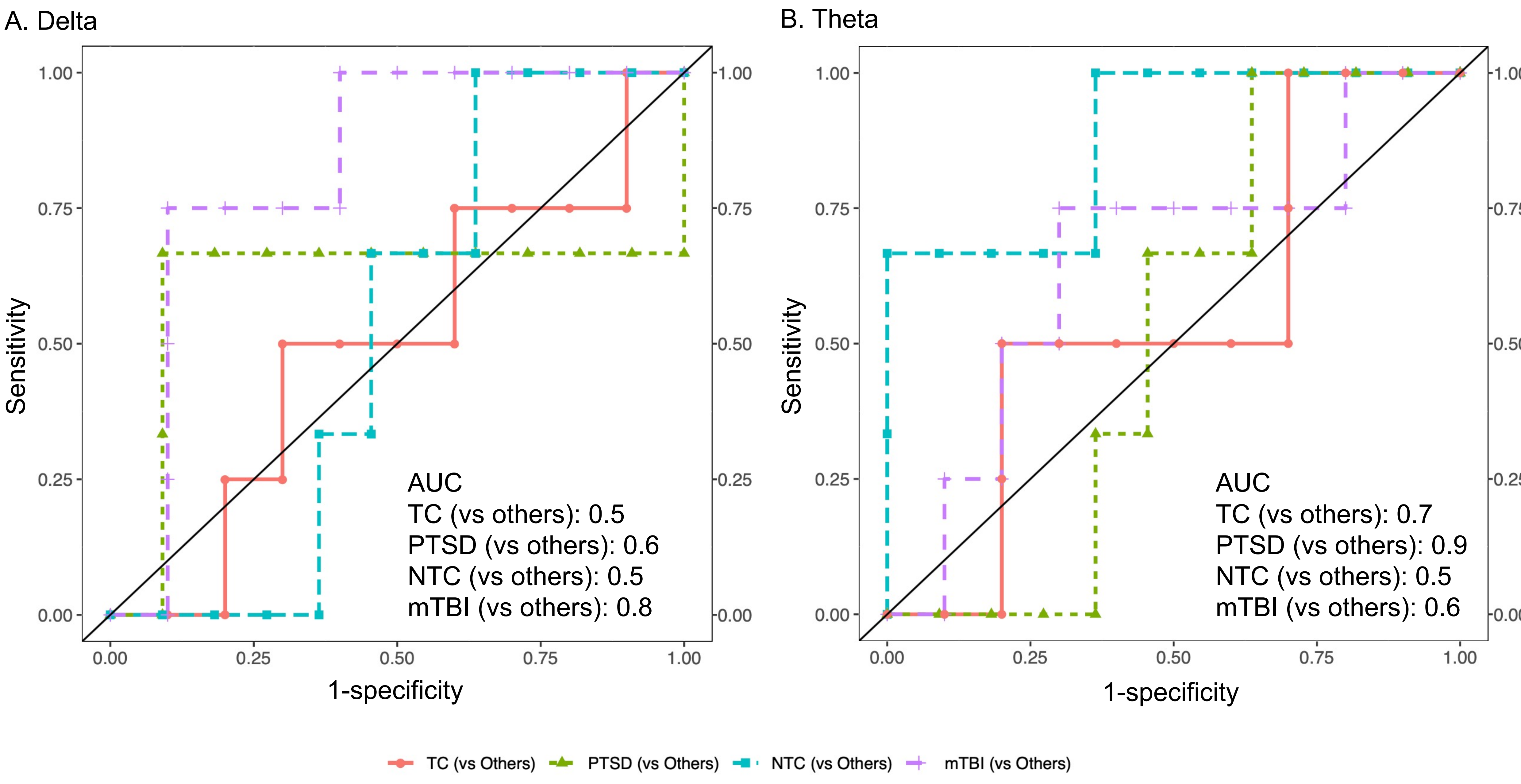

Fig. S10 – power: final ROC-AUC

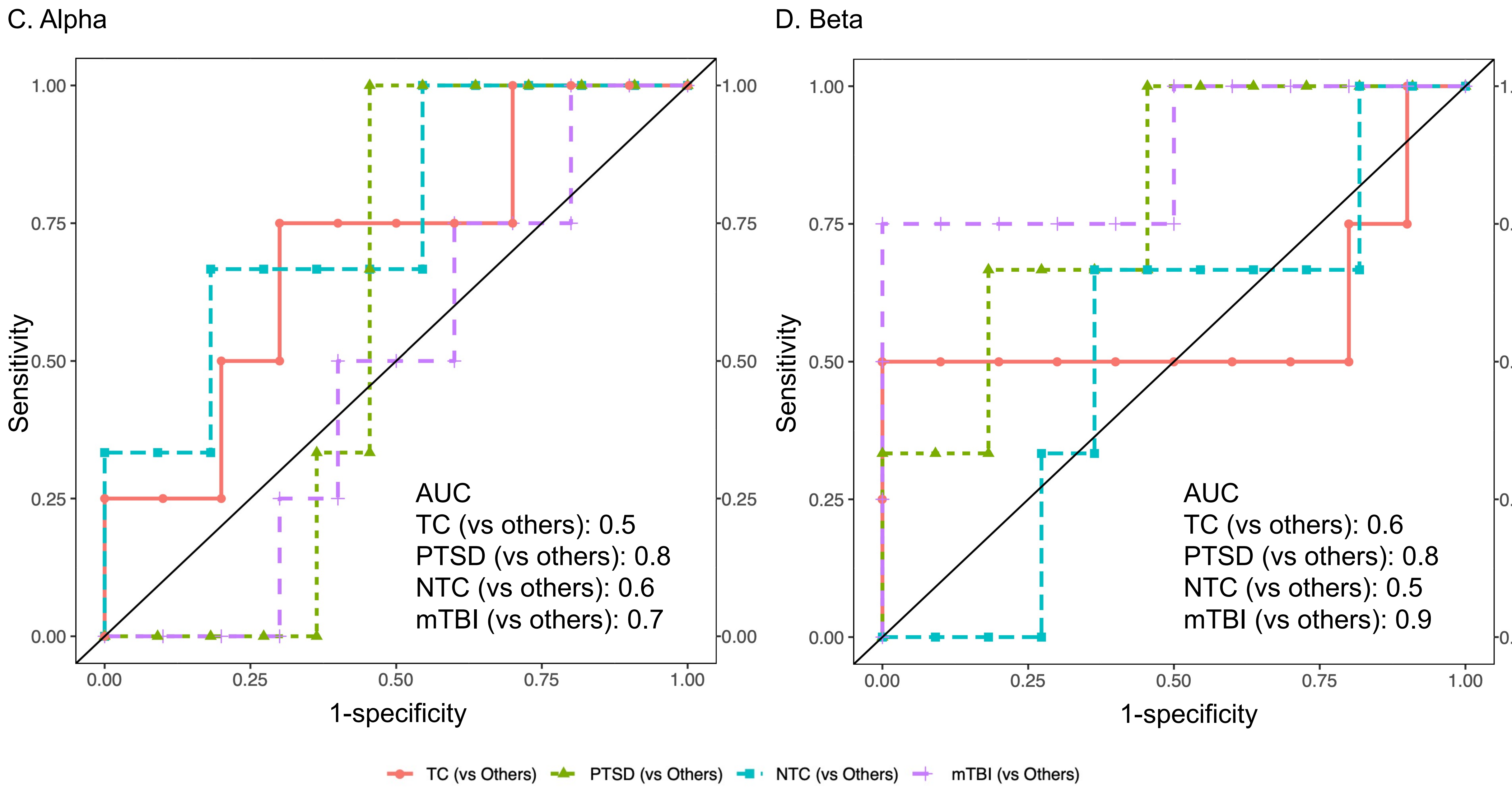

Fig. S10 – power: final ROC-AUC

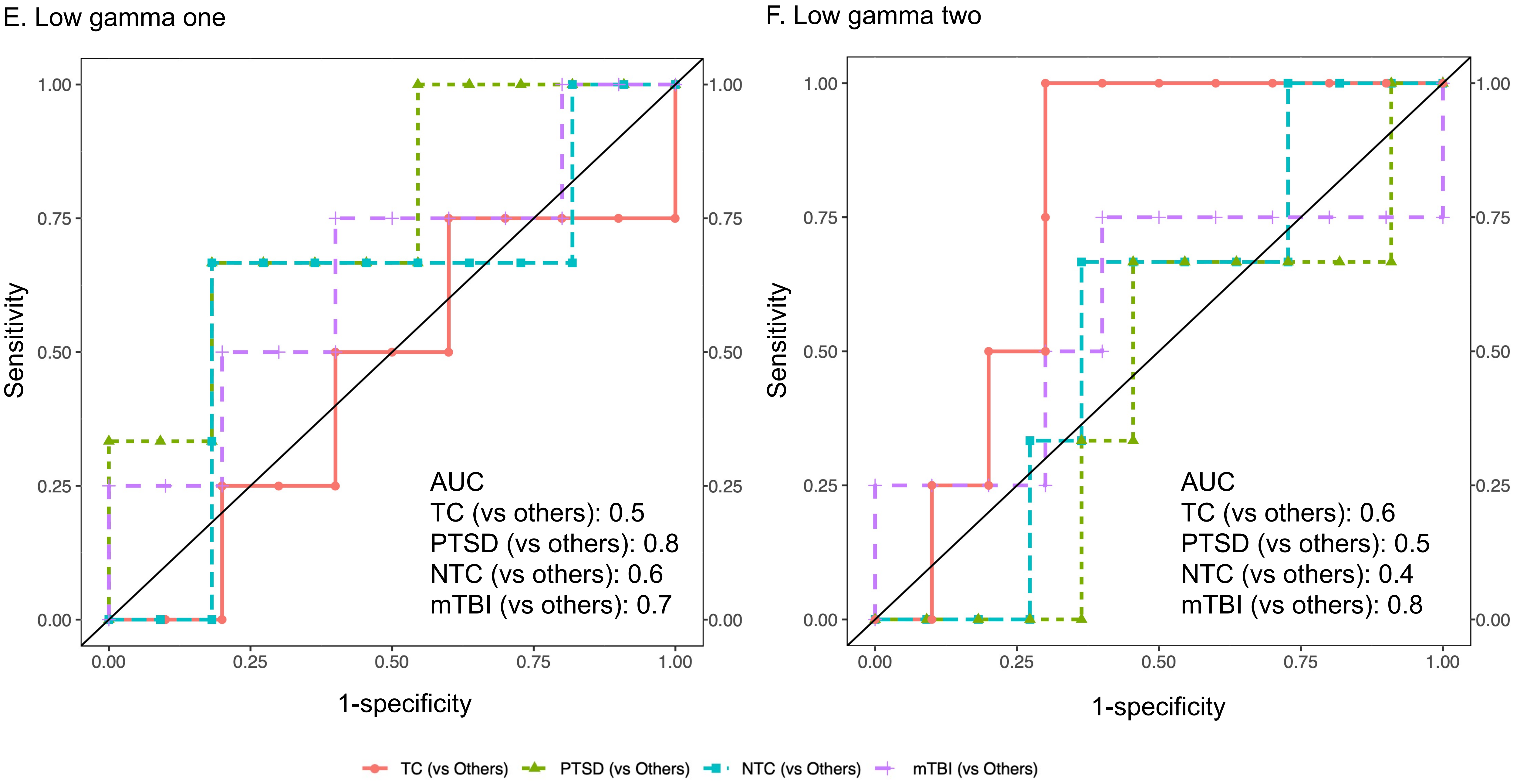

Fig. S10 – power: final ROC-AUC

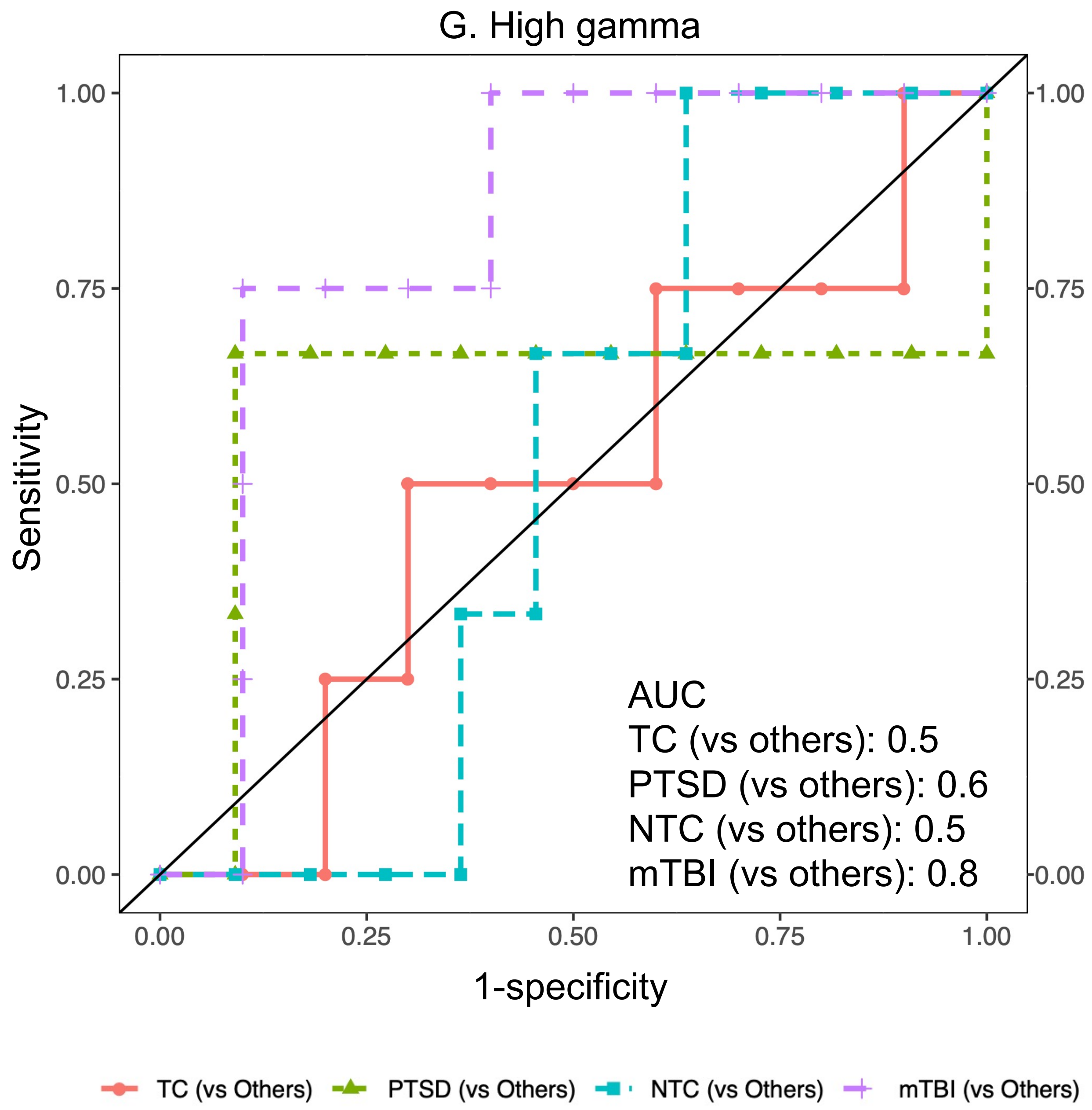

Fig. S11 – AEC: final ROC-AUC

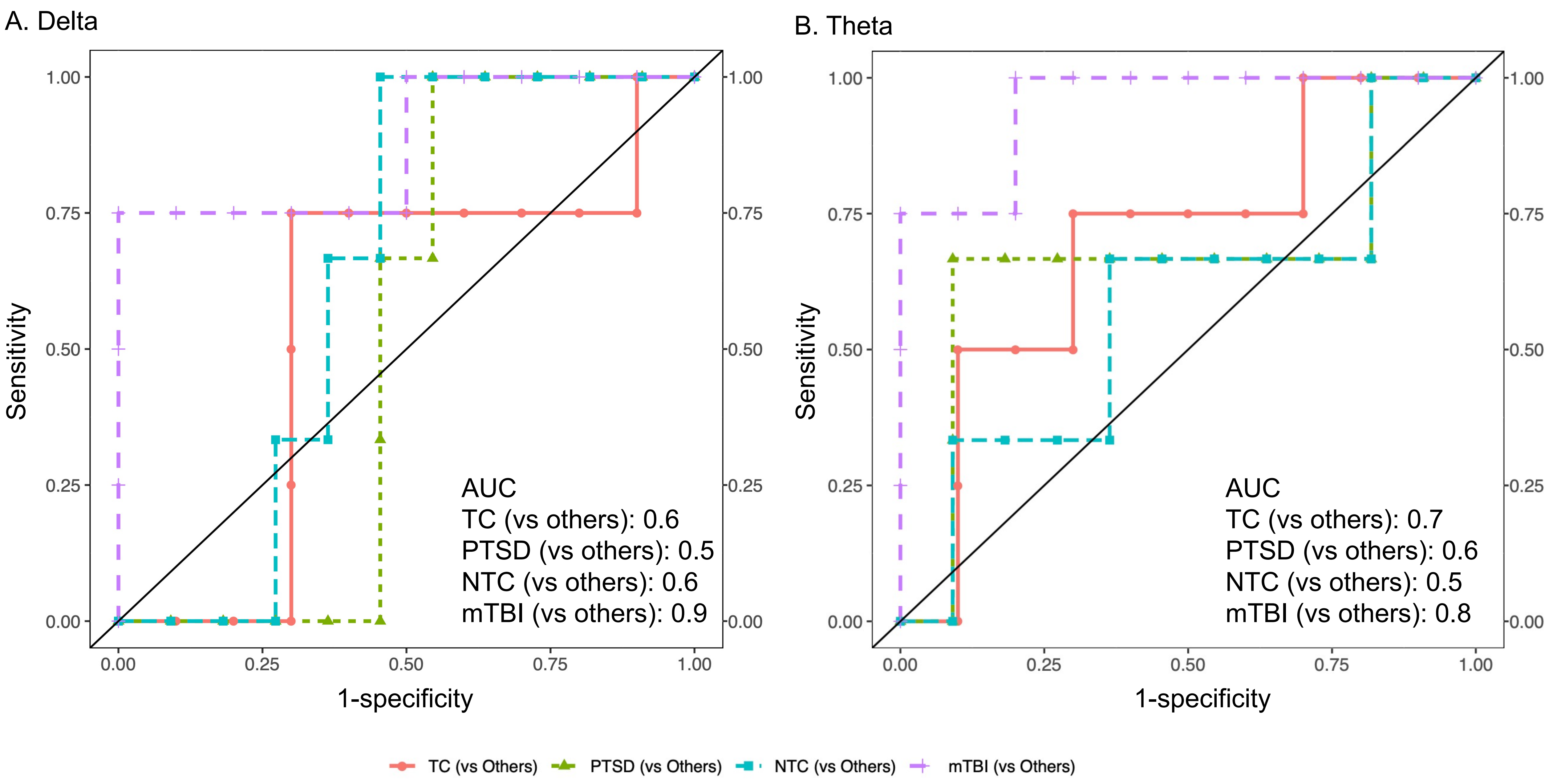

Fig. S11 – AEC: final ROC-AUC

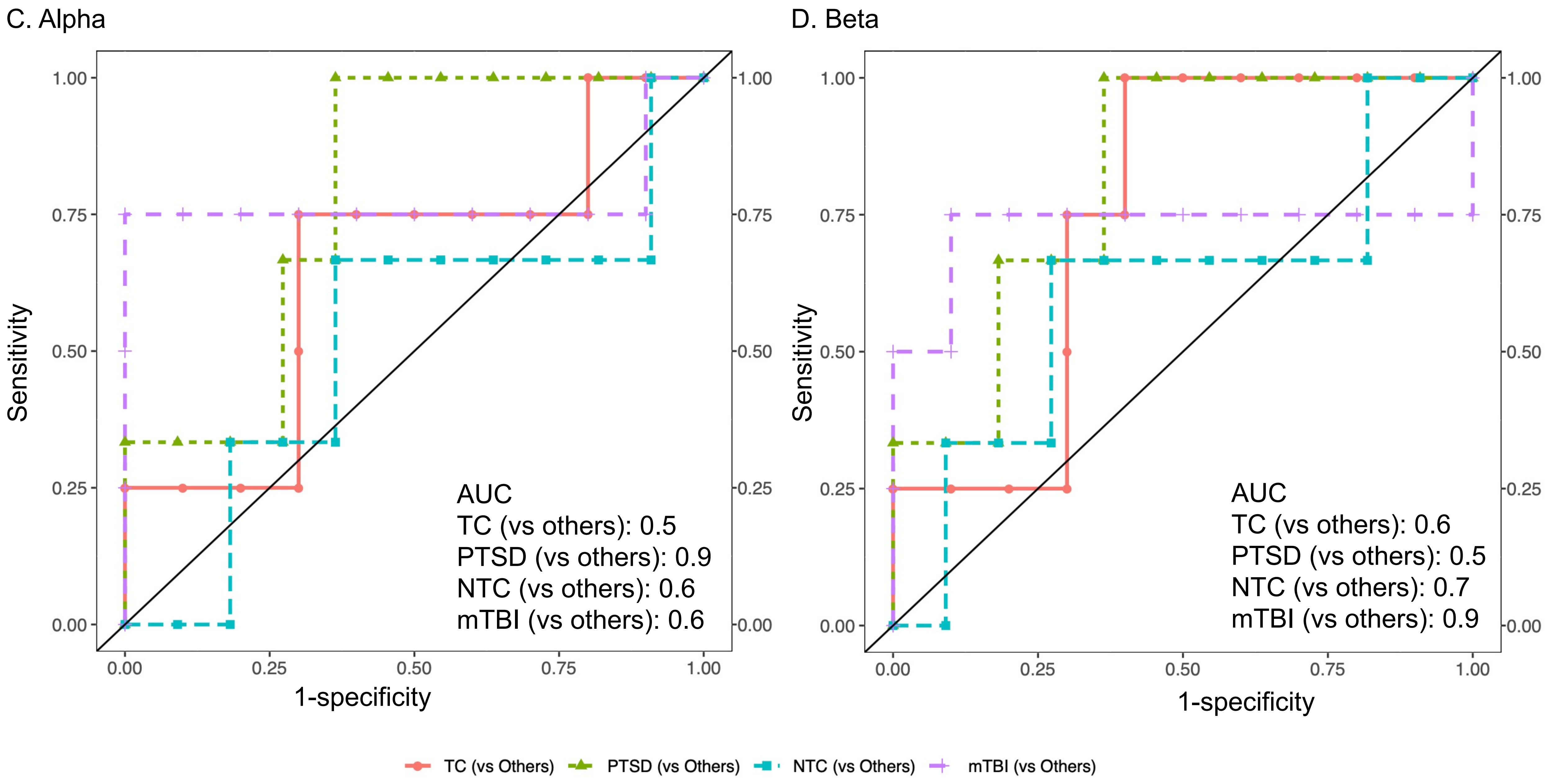

Fig. S11 – AEC: final ROC-AUC

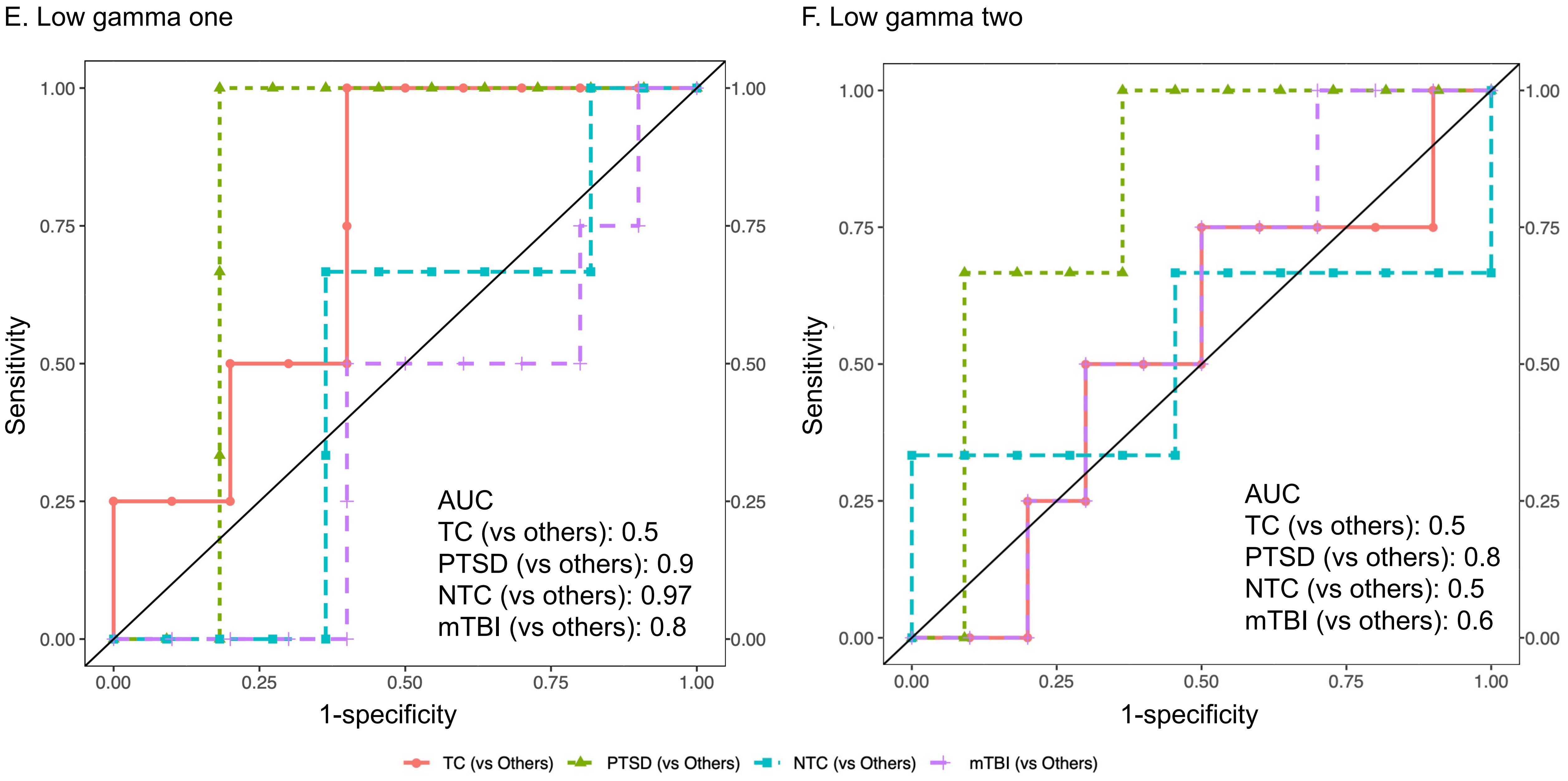

Fig. S11 – AEC: final ROC-AUC

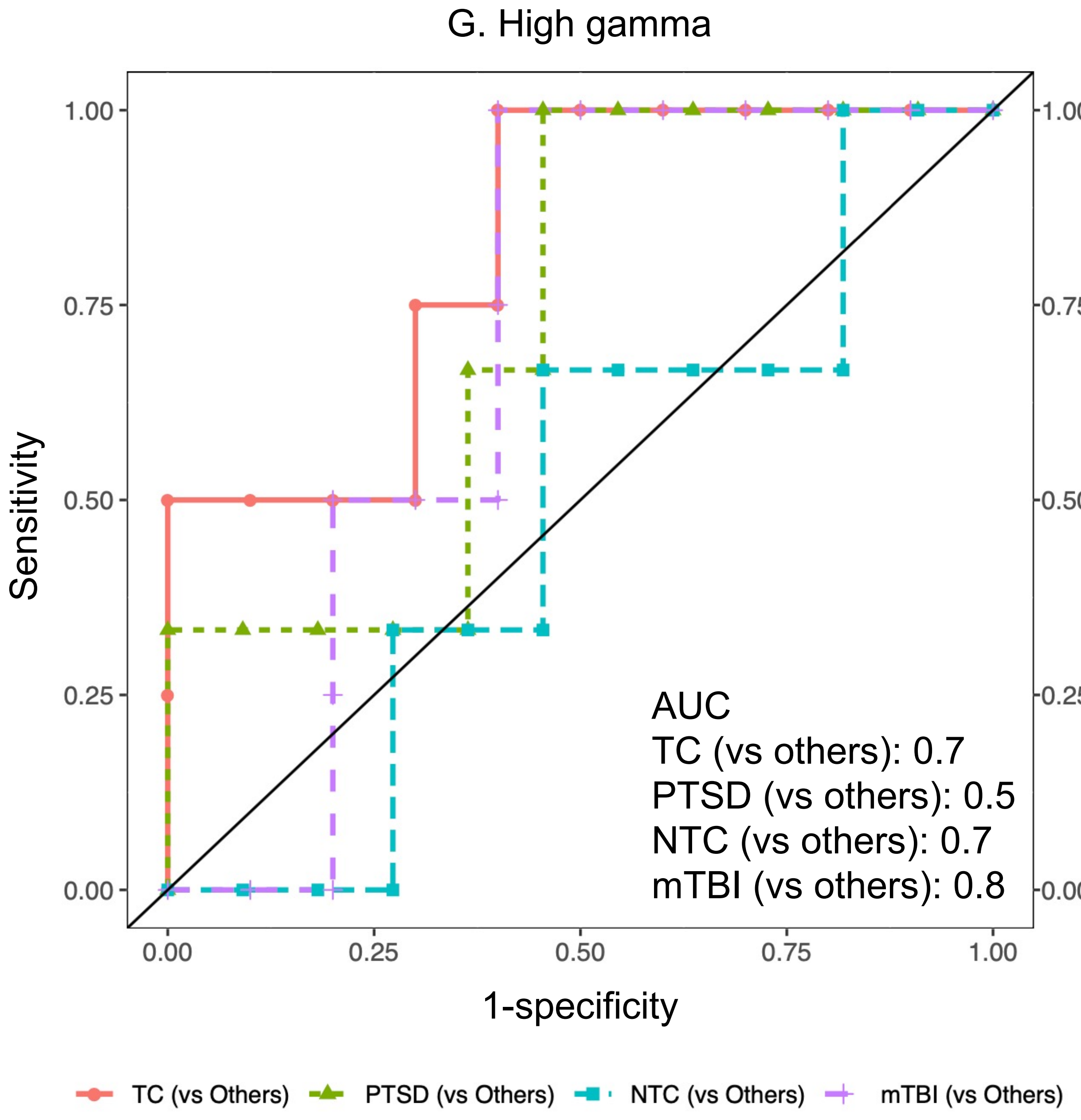

Supplement: Supplementary file 3 — Supplementary figures S1-S11 [file 41398_2021_1467_MOESM3_ESM.pdf]
