# Supplementary material for: Teasing apart trauma: neural oscillations differentiate individual cases of mild traumatic brain injury from post-traumatic stress disorder even when symptoms overlap
Source: Transl Psychiatry. 2021 Jun 4;11:345. doi: 10.1038/s41398-021-01467-8 (PMC8178364; doi:10.1038/s41398-021-01467-8)
Supplement: Supplementary file 6 — Table S3 [file 41398_2021_1467_MOESM6_ESM.docx]

**Table S3**. Consensus feature list from machine learning feature selection

A. Regional power

| Frequency | Selected regions | Region AAL code |
| --- | --- | --- |
| Delta | Right superior occipital gyrus | Occipital_Sup_R |
|  | Left Inferior frontal gyrus | Frontal_Inf_Oper_L |
|  | Right angular gyrus | Angular_R |
|  | Left cuneus | Cuneus_L |
|  | Left transverse temporal gyrus (Heschl’s gyrus) | Heschl_L |
|  | Left inferior parietal lobule | Parietal_Inf_L |
|  | Right paracentral lobule | Paracentral_Lobule_R |
|  | Right superior parietal gyrus | Parietal_Sup_R |
|  | Left gyrus rectus | Rectus_L |
|  | Left temporal pole: superior  temporal gyrus | Temporal_Pole_Sup_L |
|  | Left superior temporal gyrus | Temporal_Sup_L |
| Theta | Left amygdala | Amygdala_L |
|  | Right Rolandic operculum | Rolandic_Oper_R |
|  | Left superior parietal gyrus | Parietal_Sup_L |
|  | Left superior temporal gyrus | Temporal_Sup_L |
|  | Right superior frontal gyrus (medial) | Frontal_Sup_Medial_R |
|  | Left lenticular nucleus (pallidum) | Pallidum_L |
|  | Right cuneus | Cuneus_R |
|  | Right hippocampus | Hippocampus_R |
|  | Right insula | Insula_R |
|  | Right parahippocampal gyrus | ParaHippocampal_R |
|  | Right calcarine sulcus | Calcarine_R |
|  | Left anterior cingulate and  paracingulate gyri | Cingulum_Ant_L |
|  | Left Inferior frontal gyrus | Frontal_Inf_Oper_L |
|  | Left superior frontal gyrus (medial) | Frontal_Sup_Medial_L |
|  | Left superior frontal gyrus (orbital) | Frontal_Sup_Orb_L |
|  | Right transverse temporal gyrus (Heschl’s gyrus) | Heschl_R |
|  | Right putamen | Putamen_R |
| Alpha | Left superior parietal gyrus | Parietal_Sup_L |
|  | Left precuneus | Precuneus_L |
|  | Right insula | Insula_R |
|  | Right angular gyrus | Angular_R |
|  | Right calcarine sulcus | Calcarine_R |
|  | Right middle occipital gyrus | Occipital_Mid_R |
|  | Right precuneus | Precuneus_R |
|  | Right superior parietal gyrus | Parietal_Sup_R |
|  | Left caudate nucleus | Caudate_L |
|  | Left angular gyrus | Angular_L |
|  | Right lingual gyrus | Lingual_R |
|  | Right lenticular nucleus (pallidum) | Pallidum_R |
|  | Right putamen | Putamen_R |
|  | Left posterior cingulate gyrus | Cingulum_Post_L |
|  | Right superior occipital gyrus | Occipital_Sup_R |
|  | Left Rolandic operculum | Rolandic_Oper_L |
|  | Left middle temporal gyrus | Temporal_Mid_L |
| Beta | Right superior frontal gyrus (medial) | Frontal_Sup_Medial_R |
|  | Right temporal pole: middle temporal gyrus | Temporal_Pole_Mid_R |
|  | Left superior frontal gyrus (medial) | Frontal_Sup_Medial_L |
|  | Right middle occipital gyrus | Occipital_Mid_R |
|  | Left postcentral gyrus | Postcentral_L |
|  | Left superior frontal gyrus (orbital) | Frontal_Sup_Orb_L |
|  | Left amygdala | Amygdala_L |
|  | Right temporal pole: superior  temporal gyrus | Temporal_Pole_Sup_R |
|  | Left superior frontal gyrus (medial orbital) | Frontal_Med_Orb_L |
|  | Right parahippocampal gyrus | ParaHippocampal_R |
| Low gamma one | Left olfactory | Olfactory_L |
|  | Right calcarine sulcus | Calcarine_R |
|  | Right lingual gyrus | Lingual_R |
|  | Left Cuneus | Cuneus_L |
|  | Left supramarginal gyrus | SupraMarginal_L |
|  | Right temporal pole: superior  temporal gyrus | Temporal_Pole_Sup_R |
|  | Left precuneus | Precuneus_L |
|  | Right angular gyrus | Angular_R |
|  | Right inferior frontal gyrus (orbital part) | Frontal_Inf_Orb_R |
|  | Left superior frontal gyrus (medial) | Frontal_Sup_Medial_L |
|  | Right olfactory | Olfactory_R |
|  | Right postcentral gyrus | Postcentral_R |
|  | Right precuneus | Precuneus_R |
|  | Right gyrus rectus | Rectus_R |
|  | Right supplementary motor area | Supp_Motor_Area_R |
|  | Left temporal pole: middle temporal gyrus | Temporal_Pole_Mid_L |
| Low gamma two | Left precuneus | Precuneus_L |
|  | Left Cuneus | Cuneus_L |
|  | Left middle temporal gyrus | Temporal_Mid_L |
|  | Left inferior occipital gyrus | Occipital_Inf_L |
|  | Right posterior cingulate gyrus | Cingulum_Post_R |
|  | Right superior frontal gyrus (medial orbital) | Frontal_Med_Orb_R |
|  | Right precental gyrus | Precentral_R |
|  | Left posterior cingulate gyrus | Cingulum_Post_L |
|  | Right cuneus | Cuneus_R |
|  | Right superior occipital gyrus | Occipital_Sup_R |
|  | Right postcentral gyrus | Postcentral_R |
|  | Left calcarine sulcus | Calcarine_L |
|  | Left superior occipital gyrus | Occipital_Sup_L |
|  | Right putamen | Putamen_R |
| High gamma | Left angular gyrus | Angular_L |
|  | Right precuneus | Precuneus_R |
|  | Left superior temporal gyrus | Temporal_Sup_L |
|  | Right superior temporal gyrus | Temporal_Sup_R |
|  | Right middle frontal gyrus | Frontal_Mid_R |
|  | Left superior occipital gyrus | Occipital_Sup_L |
|  | Left precuneus | Precuneus_L |
|  | Left posterior cingulate gyrus | Cingulum_Post_L |
|  | Right posterior cingulate gyrus | Cingulum_Post_R |
|  | Right superior frontal gyrus (medial orbital) | Frontal_Med_Orb_R |
|  | Left temporal pole: middle temporal gyrus | Temporal_Pole_Mid_L |
|  | Right temporal pole: superior  temporal gyrus | Temporal_Pole_Sup_R |

B. functional connectivity (AEC)

| Frequency | Selected functional edges | Edges with region AAL code |
| --- | --- | --- |
| Delta | Superior frontal gyrus (orbital part)-to- | Frontal_Sup_Orb_R: Frontal_Mid_Orb_L |
|  | Right insula-to-left middle frontal gyrus (orbital part) | Insula_R: Cingulum_Mid_R |
|  | Left median cingulate and  paracingulate gyri-to-left angular gyrus | Cingulum_Mid_L: Angular_L |
|  | Right superior frontal gyrus-to-right angular gyrus | Frontal_Sup_R: Angular_R |
|  | Right inferior parietal lobule-to-Right angular gyrus | Parietal_Inf_R: Paracentral_Lobule_L |
|  | Left superior parietal gyrus-to-left caudate nucleus | Parietal_Sup_L: Caudate_L |
|  | Right inferior parietal lobule-to-left caudate nucleus | Parietal_Inf_R: Caudate_L |
|  | Right inferior frontal gyrus (orbital part)-to-right putamen | Frontal_Inf_Orb_R: Putamen_R |
|  | Right insula-to-left thalamus | Insula_R: Thalamus_L |
|  | Left median cingulate and  paracingulate gyri-to-right thalamus | Cingulum_Mid_L: Thalamus_R |
|  | Right postcentral gyrus-to-right thalamus | Postcentral_R: Thalamus_R |
|  | Left inferior parietal lobule-to-right transverse temporal gyrus (Heschl’s gyrus) | Parietal_Inf_L: Heschl_R |
|  | Left lenticular nucleus (pallidum)-to-right transverse temporal gyrus (Heschl’s gyrus) | Pallidum_L: Heschl_R |
|  | Right middle occipital gyrus-to-left temporal pole: middle temporal gyrus | Occipital_Mid_R: Temporal_Pole_Mid_L |
| Theta | Inferior frontal gyrus (opercular part)-to-right insula | Frontal_Inf_Oper_R: Insula_R |
|  | Left gyrus rectus-to-left hippocampus | Rectus_L: Hippocampus_L |
|  | Right parahippocampal gyrus-to-right angular gyrus | ParaHippocampal_R: Angular_R |
|  | Inferior frontal gyrus  (triangular part)-to-left precuneus | Frontal_Inf_Tri_L: Precuneus_L |
|  | Right inferior parietal lobule-to-left putamen | Parietal_Inf_R: Putamen_L |
|  | Right gyrus rectus-to-right thalamus | Rectus_R: Thalamus_R |
|  | Left superior occipital gyrus-to-left transverse temporal gyrus | Occipital_Sup_L: Heschl_L |
|  | Left inferior occipital gyrus-to-right transverse temporal gyrus (Heschl’s gyrus) | Occipital_Inf_L: Heschl_R |
|  | Left transverse temporal gyrus-to-right transverse temporal gyrus | Heschl_L: Heschl_R |
|  | Left supramarginal gyrus-to-left superior temporal gyrus | SupraMarginal_L: Temporal_Sup_L |
|  | Left precuneus-to-left middle temporal gyrus | Precuneus_L: Temporal_Mid_L |
| Alpha | Left superior frontal gyrus (medial orbital)-to-right superior frontal gyrus (medial orbital) | Frontal_Med_Orb_L: Frontal_Med_Orb_R |
|  | Left parahippocampal gyrus-to-right amygdala | ParaHippocampal_L: Amygdala_R |
|  | Right superior occipital gyrus-to-left precuneus | Occipital_Sup_R: Precuneus_L |
|  | Right precuneus-to-right paracentral lobule | Precuneus_R: Paracentral_Lobule_R |
|  | Left olfactory-to-right caudate nucleus | Olfactory_L: Caudate_R |
|  | Right cuneus-to-left temporal pole: superior  temporal gyrus | Cuneus_R: Temporal_Pole_Sup_L |
|  | Right transverse temporal gyrus (Heschl’s gyrus)-to-right temporal pole: superior  temporal gyrus | Heschl_R: Temporal_Pole_Sup_R |
| Beta | Left superior frontal gyrus (medial)-to-right gyrus recuts | Frontal_Sup_Medial_L: Rectus_R |
|  | Left parahippocampal gyrus-to-right calcarine sulcus | ParaHippocampal_L: Calcarine_R |
|  | Right precental gyrus -to-right lingual gyrus | Precentral_R: Lingual_R |
|  | Left median cingulate and  paracingulate gyri-to-left postcentral gyrus | Cingulum_Mid_L: Postcentral_L |
|  | Left postcentral gyrus-to-right postcentral gyrus | Postcentral_L: Postcentral_R |
|  | Left Cuneus-to-right superior parietal gyrus | Cuneus_L: Parietal_Sup_R |
|  | Left superior parietal gyrus-to-right precuneus | Parietal_Sup_L: Precuneus_R |
|  | Right precuneus-to-left paracentral lobule | Precuneus_R: Paracentral_Lobule_L |
|  | Left inferior parietal lobule-to-right paracentral lobule | Parietal_Inf_L: Paracentral_Lobule_R |
|  | -to-right superior temporal gyrus | Frontal_Inf_Tri_R: Temporal_Sup_R |
|  | Right parahippocampal gyrus-to-right temporal pole: superior  temporal gyrus | ParaHippocampal_R: Temporal_Pole_Sup_R |
| Low gamma one | Right middle frontal gyrus-to-left supplementary motor area | Frontal_Mid_R: Supp_Motor_Area_L |
|  | Right inferior frontal gyrus (orbital part)-to-left gyrus rectus | Frontal_Inf_Orb_R: Rectus_L |
|  | -to-right parahippocampal gyrus | Frontal_Inf_Tri_R: ParaHippocampal_R |
|  | Superior frontal gyrus (orbital part)-to-left calcarine sulcus | Frontal_Sup_Orb_R: Calcarine_L |
|  | Inferior frontal gyrus (opercular part)-to-left calcarine sulcus | Frontal_Inf_Oper_R: Calcarine_L |
|  | Right insula-to-left Cuneus | Insula_R: Cuneus_L |
|  | Right precentral gyrus-to-left postcentral gyrus | Precentral_R: Postcentral_L |
|  | -to-left supramarginal gyrus | Frontal_Med_Orb_R: SupraMarginal_L |
|  | Left superior occipital gyrus-to-right angular gyrus | Occipital_Sup_L: Angular_R |
|  | Right middle occipital gyrus-to-left paracentral lobule | Occipital_Mid_R: Paracentral_Lobule_L |
|  | Left superior frontal gyrus (medial orbital)-to-left thalamus | Frontal_Med_Orb_L: Thalamus_L |
|  | Right middle occipital gyrus-to-left transverse temporal gyrus | Occipital_Mid_R: Heschl_L |
|  | Right superior frontal gyrus (medial orbital)-to-right transverse temporal gyrus (Heschl’s gyrus) | Frontal_Med_Orb_R: Heschl_R |
|  | Right superior occipital gyrus to- | Occipital_Sup_R: Temporal_Pole_Sup_L |
|  | Right paracentral lobule-to-left temporal pole: superior  temporal gyrus | Paracentral_Lobule_R: Temporal_Pole_Mid_L |
| Low gamma two | Right middle frontal gyrus-to-left supplementary motor area | Frontal_Mid_R: Supp_Motor_Area_L |
|  | Left superior frontal gyrus (  dorsolateral)-to-right olfactory | Frontal_Sup_L: Olfactory_R |
|  | Left anterior cingulate and  paracingulate gyri-to-left median cingulate and  paracingulate gyri | Cingulum_Ant_L: Cingulum_Mid_L |
|  | Right precental gyrus -to-right hippocampus | Precentral_R: Hippocampus_R |
|  | Left superior frontal gyrus (medial)-to- right lingual gyrus | Frontal_Sup_Medial_L: Lingual_R |
|  | Right middle occipital gyrus-to-Left fusiform gyrus | Occipital_Mid_R: Fusiform_L |
|  | Right superior frontal gyrus (medial)-to- | Frontal_Sup_Medial_R: Postcentral_L |
|  | Right superior frontal gyrus (medial orbital)-to-left postcentral gyrus | Frontal_Med_Orb_R: Postcentral_L |
|  | Left middle occipital gyrus-to-left postcentral gyrus | Occipital_Mid_L: Postcentral_L |
|  | Inferior frontal gyrus  (triangular part)-to-left angular gyrus | Frontal_Inf_Tri_L: Angular_L |
|  | Left calcarine sulcus-to-right caudate nucleus | Calcarine_L: Caudate_R |
|  | Superior frontal gyrus (orbital part)-to-left lenticular nucleus (pallidum) | Frontal_Sup_Orb_R: Pallidum_L |
|  | Right cuneus-to-left temporal pole: superior  temporal gyrus | Cuneus_R: Temporal_Pole_Sup_L |
|  | Right middle occipital gyrus-to-left middle temporal gyrus | Occipital_Mid_R: Temporal_Mid_L |
|  | -to-left middle temporal gyrus | Occipital_Inf_R: Temporal_Mid_L |
|  | Right lingual gyrus-to- | Lingual_R: Temporal_Mid_R |
| High gamma | Left Rolandic operculum-to-left superior frontal gyrus (medial orbital) | Rolandic_Oper_L: Frontal_Med_Orb_L |
|  | Left hippocampus-to-left amygdala | Hippocampus_L: Amygdala_L |
|  | Left superior frontal gyrus (  dorsolateral)-to-Left middle occipital gyrus | Frontal_Sup_L: Occipital_Mid_L |
|  | Right superior frontal gyrus-to-left inferior occipital gyrus | Frontal_Sup_R: Occipital_Inf_L |
|  | Right supplementary motor area-to-left postcentral gyrus | Supp_Motor_Area_R: Postcentral_L |
|  | Left superior frontal gyrus (medial orbital)-to-right superior parietal gyrus | Frontal_Med_Orb_L: Parietal_Sup_R |
|  | Right precentral gyrus-to-left inferior parietal lobule | Precentral_R: Parietal_Inf_L |
|  | Left superior frontal gyrus (medial)-to-left inferior parietal lobule | Frontal_Sup_Medial_L: Parietal_Inf_L |
|  | -to-right angular gyrus | Precentral_L: Angular_R |
|  | Right insula-to-right angular gyrus | Insula_R: Angular_R |
|  | Right postcentral gyrus-to-Left putamen | Postcentral_R: Putamen_L |
|  | Right thalamus-to-right transverse temporal gyrus (Heschl’s gyrus) | Thalamus_R: Heschl_R |
