# Supplementary material for: Teasing apart trauma: neural oscillations differentiate individual cases of mild traumatic brain injury from post-traumatic stress disorder even when symptoms overlap
Source: Transl Psychiatry. 2021 Jun 4;11:345. doi: 10.1038/s41398-021-01467-8 (PMC8178364; doi:10.1038/s41398-021-01467-8)
Supplement: Supplementary file 7 — Table S4 [file 41398_2021_1467_MOESM7_ESM.docx]

**Table S4.** SVM modelling CV AUC

A. Regional power

| **Frequency** | **TC** | **PTSD** | **NTC** | **mTBI** |
| --- | --- | --- | --- | --- |
| Delta | 0.74±0.19 | 0.78±0.20 | 0.70±0.17 | 0.58±0.12 |
| Theta | 0.64±0.14 | 0.69±0.15 | 0.69±0.14 | 0.76±0.20 |
| Alpha | 0.72±0.11 | 0.73±0.17 | 0.68±0.15 | 0.65±0.13 |
| Beta | 0.68±0.13 | 0.64±0.09 | 0.74±0.21 | 0.67±0.14 |
| Low gamma one | 0.75±0.17 | 0.66±0.17 | 0.66±0.08 | 0.68±0.17 |
| Low gamma two | 0.69±0.11 | 0.71±0.15 | 0.75±0.17 | 0.60±0.14 |
| High gamma | 0.69±0.10 | 0.68±0.19 | 0.78±0.16 | 0.62±0.16 |

B. AEC

| **Frequency** | **TC** | **PTSD** | **NTC** | **mTBI** |
| --- | --- | --- | --- | --- |
| Delta | 0.64±0.13 | 0.68±0.19 | 0.76±0.18 | 0.73±0.18 |
| Theta | 0.61±0.09 | 0.69±0.24 | 0.71±0.15 | 0.70±0.19 |
| Alpha | 0.70±0.17 | 0.71±0.13 | 0.68±0.19 | 0.64±0.20 |
| Beta | 0.64±0.14 | 0.74±0.17 | 0.70±0.16 | 0.68±0.14 |
| Low gamma one | 0.62±0.18 | 0.66±0.19 | 0.64±0.11 | 0.68±0.15 |
| Low gamma two | 0.68±0.14 | 0.66±0.21 | 0.73±0.17 | 0.73±0.09 |
| High gamma | 0.66±0.20 | 0.65±0.18 | 0.70±0.16 | 0.68±0.12 |
